# N-heterocyclic carbene-stabilized metal nanoparticles within porous organic cages for catalytic application

**DOI:** 10.1093/nsr/nwac067

**Published:** 2022-04-05

**Authors:** Tong Liu, Sha Bai, Le Zhang, F Ekkehardt Hahn, Ying-Feng Han

**Affiliations:** Key Laboratory of Synthetic and Natural Functional Molecule of the Ministry of Education, Xi'an Key Laboratory of Functional Supramolecular Structure and Materials, College of Chemistry and Materials Science, Northwest University, Xi’an 710127, China; Key Laboratory of Synthetic and Natural Functional Molecule of the Ministry of Education, Xi'an Key Laboratory of Functional Supramolecular Structure and Materials, College of Chemistry and Materials Science, Northwest University, Xi’an 710127, China; Key Laboratory of Synthetic and Natural Functional Molecule of the Ministry of Education, Xi'an Key Laboratory of Functional Supramolecular Structure and Materials, College of Chemistry and Materials Science, Northwest University, Xi’an 710127, China; Key Laboratory of Synthetic and Natural Functional Molecule of the Ministry of Education, Xi'an Key Laboratory of Functional Supramolecular Structure and Materials, College of Chemistry and Materials Science, Northwest University, Xi’an 710127, China; Institut für Anorganicshe und Analytische Chemie, Westfälische Wilhelms-Universität Münster, Münster 48149, Germany; Key Laboratory of Synthetic and Natural Functional Molecule of the Ministry of Education, Xi'an Key Laboratory of Functional Supramolecular Structure and Materials, College of Chemistry and Materials Science, Northwest University, Xi’an 710127, China

**Keywords:** metal nanoparticle, porous organic cage, catalytic reaction, N-heterocyclic carbene

## Abstract

Tuning the surface-embellishing ligands of metal nanoparticles (NPs) is a powerful strategy to modulate their morphology and surface electronic and functional features, impacting their catalytic activity and selectivity. In this work, we report the design and synthesis of a polytriazolium organic cage PIC-**T**, capable of stabilizing PdNPs within its discrete cavity. The obtained material (denoted Pd@PCC-**T**) is highly durable and monodispersed with narrow particle-size distribution of 2.06 ± 0.02 nm, exhibiting excellent catalytic performance and recyclability in the Sonogashira coupling and tandem reaction to synthesize benzofuran derivatives. Further investigation indicates that the modulation of N-heterocyclic carbene sites embedded in the organic cage has an impact on NPs’ catalytic efficiency, thus providing a novel methodology to design superior NP catalysts.

## Teaser

The discrete cage structure and a subtle regulation of NHC sites inside the cavity facilitate the controllable formation of unique PdNPs with superior catalytic performances.

## INTRODUCTION

In recent years, porous organic cages with intrinsic inner cavities and tunable surfaces have emerged as a new class of microporous material with promising applications in recognition [1], molecular separation [2] and catalysis [3]. Differently to other porous materials such as metal-organic frameworks and zeolites [4,5], porous organic cages exhibit intriguing features, including structural tunability, thermal and chemical stability, and unique solution processability, making them prominent candidates for encapsulating diverse metal nanoparticles (MNPs) within their nanoporous cavities [6–13]. MNPs possess high surface area/volume ratios with many active catalytic sites, giving them exceptional catalytic activity compared with their metal counterparts [14]. Although they have been demonstrated to be critical heterogeneous catalysts for a wide array of reactions, MNPs still suffer from difficulties related to agglomeration and consequent loss of catalytic activity during catalytic processes [15]. In this regard, porous organic cages offer protecting shells for the encapsulated MNPs with minimum coverage and greater surface accessibility within their uniform but flexible cavities.

Over the past few decades, N-heterocyclic carbenes (NHCs) have established themselves as one of the most potent ligands in many catalytic systems [16–19], as well as metal-NHC-stabilized MNPs [20–27]. However, the development of discrete 3D NHC-based architectures to encapsulate MNPs with superior catalytic performance is still in its infancy [28]. Recently, our group developed an efficient supramolecular strategy named the metal-carbene template approach (MCTA) to synthesize polyimidazolium organic cages (PICs) [29–32]. Using this strategy, a new type of PIC material with different shapes and sizes can be readily tailored by the rational design of NHC precursors. PICs have good solubility and stability in most common solvents and have the potential to support MNPs [29]. More importantly, their *in-situ*-generated poly-NHC sites are essential in controlling the nucleation and growth of MNPs. The strong σ-donating ability of the NHCs further enhances the interactions between the shells (cages) and the MNPs [33], consequently leading to encapsulated MNPs within polycarbene cages (PCCs) with unique properties.

**Figure 1.**
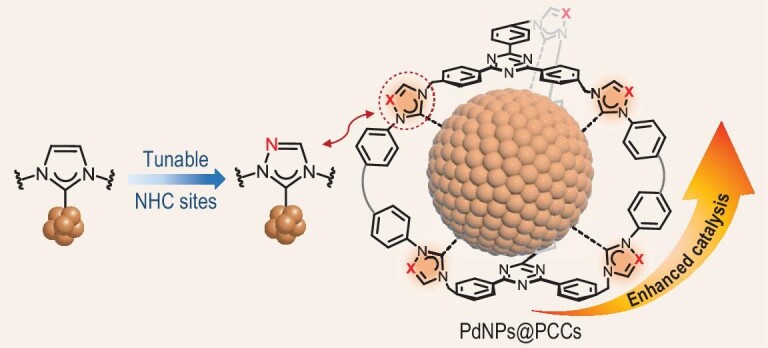
Modulation of the NHC sites embedded in Pd@PCC-**I** (X = C) and Pd@PCC-**T** (X = N) for tunable catalytic performance.

Compared to the imidazolium counterparts, replacing a carbon atom with an electronegative nitrogen atom can result in 1,2,4-triazolium moieties bearing different steric and electronic environments [34–38]. However, to the best of our knowledge, the preparation of MNPs stabilized by 1,2,4-triazolium-derived NHCs has rarely been explored [28]. Herein, we report the facile synthesis of a new polytriazolium cage named PIC-**T**, and its first use as a template for the size-controlled synthesis of PdNPs (Fig. 1). The well-defined cavity and *in-situ*-generated 1,2,4-triazolin-5-ylidenes facilitate the nucleation and stabilization of PdNPs with high dispersibility, accessibility in solution and high catalytic activities towards the coupling reactions. Remarkably, a subtle regulation of NHC sites (from imidazolidin-2-ylidene to 1,2,4-triazolin-5-ylidene) binding with PdNPs significantly boosts the catalytic activity toward the tandem reaction to synthesize benzofuran derivatives.

## RESULTS AND DISCUSSION

### Synthesis and characterization of poly-NHC-shell-stabilized PdNPs

**Figure 2.**
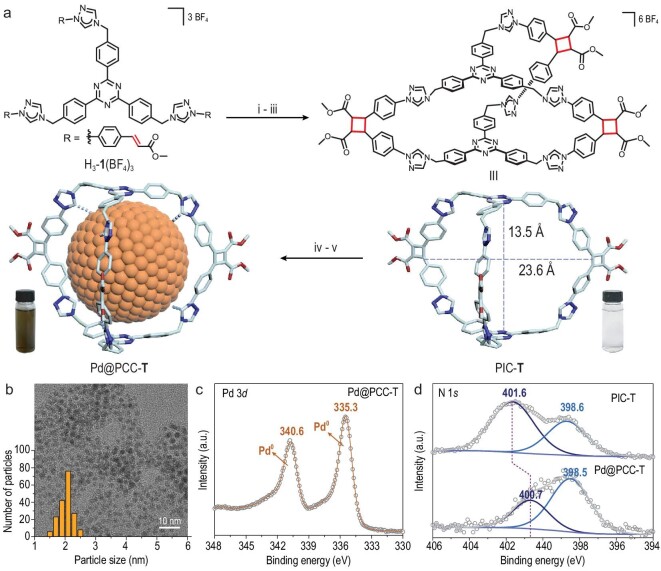
(a) The schematic synthesis of Pd@PCC-**T** by PIC-**T**. i: Ag_2_O, CH_3_CN, 65°C, 24 h; ii: *hv*, *λ* = 365 nm, 24 h; iii: NH_4_Cl, CH_3_OH, rt, 2 h and then anion exchange with NH_4_BF_4_ in CH_3_OH, rt, 2 h; iv: Pd(OAc)_2_, CH_3_CN, rt, 2 h; v: NaBH_4_, CH_3_CN and CH_3_OH, rt, 3 h. (b) TEM image (scale bar = 10 nm), size distribution and (c) Pd 3*d* XPS spectrum of Pd@PCC-**T**. (d) N 1*s* XPS spectrum of PIC-**T** and Pd@PCC-**T**.

The porous organic cage PIC-**T** was synthesized from the tris-triazolium salt H_3_-**1**(BF_4_)_3_ through the MCTA (Fig. S1). First, H_3_-**1**(BF_4_)_3_ decorated with three olefin groups on the triazolium N-wingtips reacted with Ag_2_O to generate a trinuclear hexacarbene complex [Ag_3_(**1**)_2_](BF_4_)_3_. Following photo-induced post-synthetic modification of the olefin groups, a metallosupramolecular cage was obtained via intramolecular [2+2] cycloadditions (Scheme S1). Finally, removing the Ag^+^ ions resulted in the desired polytriazolium organic cage PIC-**T** (Fig. S2), which was characterized by multinuclear nuclear magnetic resonance (NMR) analysis and electrospray ionization mass spectrometry (ESI-MS) spectrometry (see Supplementary Data for details). Density functional theory (DFT) calculations revealed that the size of the internal cage cavity is nearly identical to the reported one for the PIC-**I** functionalized with six imidazolium groups [31,32]. PIC-**T**, having six 1,2,4-triazolium groups inside its cavity, represents a good candidate for the palladium(II) salt complexation. We prepared the PdNPs via a two-step approach (Fig. 2a). First, a red-orange solution of PIC-**T** (1 equiv.) and Pd(OAc)_2_ (10 equiv.) in CH_3_CN was prepared. After stirring for 2 h, the reaction mixture was reduced by a methanolic solution of NaBH_4_, affording Pd@PCC-**T** along with a sharp color change to deep brown without any precipitation, suggesting the PdNPs formation via reduction, and stabilization by *in-situ*-generated PCCs. The ^1^H NMR analysis proved the anchoring between Pd centers and 1,2,4-triazolin-5-ylidenes and the formation of NHC species by the disappearance the characteristic proton of 1,2,4-triazolium as well as the broadening and shifting of the relevant protons of PIC-T (Fig. S3), as previously reported [8,30]. In addition, two-dimensional diffusion-ordered spectroscopy revealed that the as-synthesized Pd@PCC-**T** has a similar diffusion coefficient to the reported Au@PCC-**I** [32], which confirms the similar size and shape of Pd@PCC-**T** and Au@PCC-**I** (Fig. S3). It provides important evidence to support the idea that the formed PdNPs are entrapped within the cage. Finally, the Pd@PCC-**T** was isolated, after centrifugation, as a black powder. Remarkably, this material was well dispersed in methanol or ethanol and stable for six months without agglomeration or any significant color change under ambient conditions (Fig. S4).

The particle diameter and size distribution of Pd@PCC-**T** were analyzed by transmission electron microscopy (TEM) micrographs. The image presented well-dispersed PdNPs with a narrow size distribution of 2.06 ± 0.02 nm (Fig. 2b), matching well with the optimized cage structure (Fig. S5). This indicates that the cage template can offer a protecting shell for the size-controlled synthesis of PdNPs and the majority of PdNPs are probably anchored within the cage cavities [9]. The scanning electron microscopy (SEM) image also depicted a rough morphology consisting of granular particles of as-prepared Pd@PCC-**T** (Fig. S6); the palladium loading is estimated to be ∼22 wt%, determined by inductive coupled plasma mass spectrometry (ICP-MS) [6]. Additional energy dispersive X-ray elemental mapping analysis showed that the formed PdNPs were highly distributed inside the cage cavities of PCC-**T** (Fig. S7). The survey X-ray photoelectron spectroscopy (XPS) of Pd@PCC-**T** then confirmed the composition of Pd, C, N and O elements in the sample (Fig. S8).

Furthermore, a Pd *3d* XPS spectrum was performed to identify the chemical states of Pd in Pd@PCC-**T.** The binding energy values at 340.6 and 335.3 eV correspond well with the typical spin-orbit pairs of 3d_5/2_ and 3d_3/2_, indicative of its zero-oxidation state (Fig. 2c). To gain further insight into the electronic coupling between PCC-**T** and PdNPs, N1*s* XPS analysis was performed (Fig. 2d). The binding energy assigned to the carbene N shifted from 401.6 eV to 400.7 eV after forming Pd@PCC-**T**, definitely demonstrating the anchoring of PdNPs by the organic cage [39,40]. Moreover, high-resolution TEM (HRTEM) images revealed PdNPs with a *d*-spacing of 0.22 nm, corresponding to the (111) lattice planes of zero-valent Pd (Fig. S9) [9,41]. The powder X-ray diffraction (PXRD) pattern further disclosed that the crystal form of Pd@PCC-**T** is a face-centered cubic (fcc) structure (Fig. S10).

To evaluate the role of the cage scaffold in the synthesis of PdNPs, controlled experiments were performed. Under standard conditions, immediate precipitation caused by the aggregation of the PdNPs was observed in the absence of the PIC-**T** template (Fig. S11). In the second case, only aggregated amorphous Pd^0^ species were detected when the monodentate 1,2,4-triazol-5-ylidene-derived NHC ligands were used as templates (Fig. S12). These results further demonstrate the crucial role of the cavity confining effect in the nucleation, controlled synthesis and stabilization of PdNPs. In addition, Pd@PCC-**T** was stable even after exposure to 1-dodecanethiol (DDT, *c* = 5 mM in methanol) for 24 h, according to the TEM, XPS and PXRD analyses (Fig. S14). This synthetic strategy was further applied in preparing poly-imidazolium cage (PIC-**I**) stabilized NPs, Pd@PCC-**I**. This material, characterized in a similar manner by XPS, XRD, TEM and ICP-MS (Fig. S13), exhibited comparative particle sizes (2.06 ± 0.01 nm) and Pd loading (∼21 wt%) to Pd@PCC-**T**.

### Catalytic performance investigation into the Sonogashira reaction

To date, PdNPs have been demonstrated as superior catalysts in a variety of organic reactions [42]. For the purpose of evaluating the catalytic activity of newly formed Pd@PCC-**T**, as a proof-of-principle, the Sonogashira reaction between bromobenzene and phenylacetylene was selected as a model reaction. After optimizing a suite of reaction parameters (Table S1), the coupling reaction was carried out in the presence of Pd@PCC-**T** (0.5 mol% Pd) [9] with Et_3_N as a base in DMF at 40°C, affording compound **3a** with 99% yield within 6 h at ambient conditions and exhibiting superior activity compared with commonly used Pd catalysts (Table S2). Under standard conditions, a series of aryl bromides or iodides and phenylacetylene derivatives were investigated and the results were summarized in Table 1. Both electron-rich and electron-deficient phenylacetylenes were applicable, affording the desired coupling products **3b**–**3l** in excellent yields (88%–>99%). Note that sterically hindered aryl iodides were found to be more reactive than aryl bromides, delivering the corresponding coupling products (**3a**, **3c**, **3f**–**3h** and **3l**) not only with higher yields (96%–>99%) but also in a much shorter time (1 h vs. 6 h). The catalytic performance of Pd@PCC-**T** is superior to the majority of previously reported PdNP catalysts protected by classic organic supports, presumably due to the small particle size, available surface accessibility and the stability achieved via the well-confined organic cage [43,44].

**Table 1.** The substrate scope of Pd@PCC-**T** catalyzed Sonogashira reaction.^a^

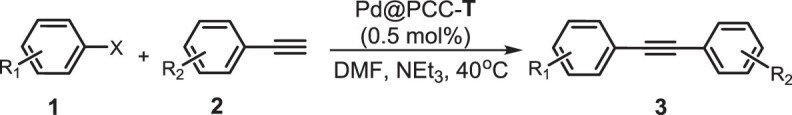						
Entry	X	R_1_	R_2_	Time (h)	Product	Yield (%)^b^
1	Br	H	H	6	**3a**	99
2	Br	H	4-CH_3_	6	**3b**	99
3	Br	H	4-OCH_3_	6	**3c**	>99
4	Br	H	4-F	6	**3d**	90
5	Br	H	4-Cl	6	**3e**	92
6	Br	H	4-CF_3_	6	**3f**	89
7	Br	H	4-C(CH_3_)_3_	6	**3g**	97
8	Br	H	4-pyridyl	6	**3h**	92
9	Br	H	3-F	6	**3i**	93
10	Br	H	3-OCH_3_	6	**3j**	96
11	Br	4-OCH_3_	4-OCH_3_	6	**3k**	95
12	Br	4-Cl	4-OCH_3_	6	**3l**	88
13	I	H	H	1	**3a**	>99
14	I	H	4-OCH_3_	1	**3c**	>99
15	I	H	4-CF_3_	1	**3f**	96
16	I	H	4-C(CH_3_)_3_	1	**3g**	>99
17	I	H	4-pyridyl	1	**3h**	97
18	I	4-Cl	4-OCH_3_	1	**3l**	98

a Reaction conditions: aryl halide (2.5 mmol), phenylacetylene (3.0 mmol), NEt_3_ (7.5 mmol), DMF (5.0 mL) and Pd@PCC-**T** (0.5 mol% Pd, 6.0 mg). ^b^Yield determined by gas chromatography (GC) analysis.

### Catalytic performance investigation into the tandem reaction

The benzofuran motif is a key structural unit in various biologically active natural products and pharmaceutical compounds, such as amurensin H, anigopreissin A, amiodarone and so on [45]. The synthesis of these benzofuran derivatives, therefore, has attracted a lot of attention. Among the different methodologies, domino Sonogashira alkynylation coupling has been established as a green approach with high atom economy. Encouraged by the above results, the Pd@PCC-**T** was further used to synthesize 2-arylbenzofuran derivatives, ubiquitous in regular prescription and potential new drugs [46]. Preliminary studies focused on the reaction between 2-iodophenol and phenylacetylene as benchmark substrates. After screening a couple of reaction parameters (Supplementary Data, Table S3), the reaction carried out in the presence of Pd@PCC-**T** (0.5 mol% Pd) with Cs_2_CO_3_ as a base provided the best result (**6a**, 98% yield) via a one-pot method (Table S3, entry 5).

**Figure 3.**
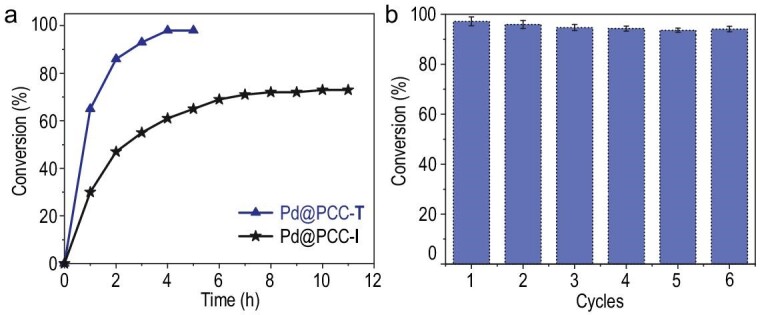
(a) Time course of the formation of 2-phenylbenzofuran by using Pd@PCC-**T** and Pd@PCC-**I** respectively. (b) Recyclability of the Pd@PCC-**T** catalyst in the tandem reaction.

Although the size-dependent catalytic activity of NPs has been intensively investigated, the functional sites modulation of organic cage supports to tailor the catalytic performance of encapsulated metal NPs has been seldom explored [47]. To investigate this issue, Pd@PCC-**I**, having a comparable particle size and Pd loading to Pd@PCC-**T** but different NHC sites, was employed as a catalyst (Table 2, entry 2). Surprisingly, the yield of the model tandem reaction significantly decreased from 98% to 61% under identical conditions. The time-dependent reaction profiles further reveal that Pd@PCC-**T** exhibits much better catalytic activity in the overall conversion and reaction rate (4 h, 98%), while Pd@PCC-**I** only affords 73% yield even after a lengthened reaction time of 10 h (Fig. 3a). Thus, the cage-stabilized PdNPs do greatly enhance the catalytic efficiency in this reaction, benefitting from subtle modulation of NHC sites. For comparison, several commonly used Pd catalysts were also tested under the established conditions. Strikingly, the yields decreased from 98% for Pd@PCC-**T** to 22%, 11% and 0% for PdCl_2_, Pd/C and [Ph_3_P]_2_PdCl_2_, respectively (Table 2, entries 3–5). Generally, adding a Cu(I) co-catalyst could accelerate the Sonogashira reaction as previously reported [48]. Indeed, combing a co-catalyst CuI with [Ph_3_P]_2_PdCl_2_ leads to a 28% yield in this system (Table 2, entry 6); however, this is still inferior. Despite a slight increase with Pd(OAc)_2_ (32%, Table 2, entry 7), the yield is much lower than the Pd@PCC-**I** catalyst (61%). In contrast, the better catalytic performance of Pd@PCCs highlights the critical role of cage confinement in enhancing catalytic efficiency in the tandem reaction. After the reaction completed, the residue solution exhibited no catalytic activity after separation of Pd@PCC-**T** from the reaction mixture, excluding the leaching of the homogeneous metal species. These findings indicate that modulation of the chemical environment (electronic effect, steric effect etc.) around PdNPs [49,50] via ligand modification at the atomic level can regulate the interaction between metal NPs and substrates for sequential catalysis, more or less.

**Table 2.** The tandem reaction for the synthesis of benzofuran derivatives catalyzed by different Pd catalysts.^a^

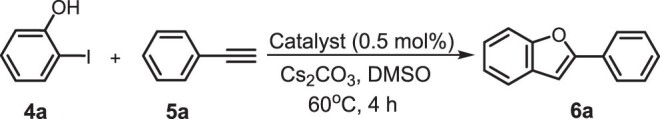			
Entry	Catalyst	Co-catalyst	Yield (%)^b^
1	Pd@PCC-**T**	/	98
2	Pd@PCC-**I**	/	61
3	PdCl_2_	/	22
4	Pd/C	/	11
5	[Ph_3_P]_2_PdCl_2_	/	0
6	[Ph_3_P]_2_PdCl_2_	CuI	28
7	Pd(OAc)_2_	/	32

a Reaction conditions: 2-iodophenol (2.5 mmol), phenylacetylene (3.0 mmol), Cs_2_CO_3_ (7.5 mmol), DMSO (6.0 mL) and Pd catalyst (0.5 mol% Pd). ^b^Yield determined by GC analysis.

**Figure 4.**
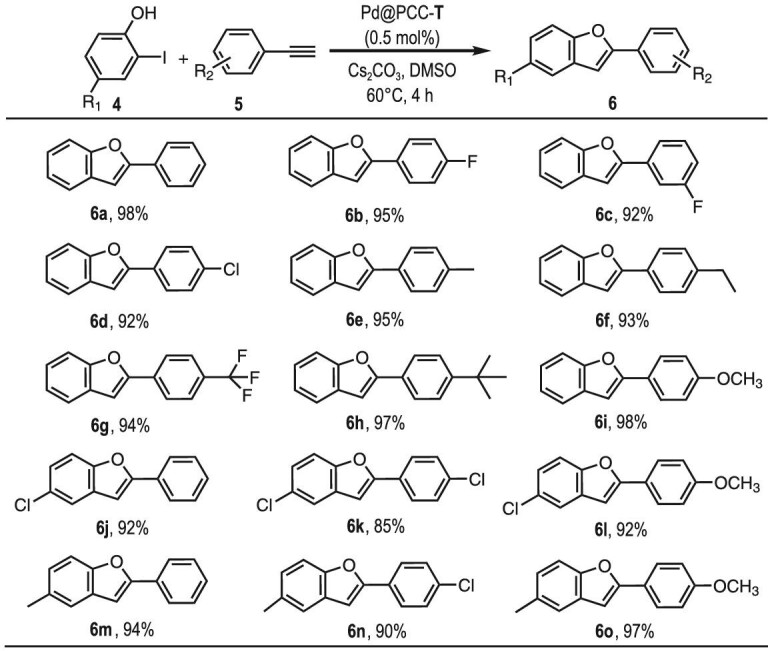
The substrate scope of the Pd@PCC-**T** catalyzed tandem reaction for the synthesis of benzofuran derivatives. Reaction conditions: 2-iodophenol (2.5 mmol), phenylacetylene (3.0 mmol), Cs_2_CO_3_ (7.5 mmol), DMSO (6.0 mL) and Pd@PCC-**T** (0.5 mol% Pd, 6.0 mg). Yield determined by GC analysis.

Pd@PCC-**T** also exhibited unique catalytic reactivity in synthesizing 2-arylbenzofuran derivatives bearing diverse functional groups (Fig. 4). The electron-withdrawing groups, that is, F (**6b** and **6c**), Cl (**6d**) and CF_3_ (**6g**), and electron-donating groups, such as Me (**6e**), Et (**6f**), *^t^*Bu (**6**h) and OMe (**6i**), gave the corresponding 2-arylbenzofuran with excellent yields (92%–98%). Furthermore, 2-iodophenol with different substituents (Cl and Me) were evaluated. It is interesting to note that 2-iodophenol containing an electron-donating group (Me; **6m**, **6n**, **6o**) gave yields (90%–97%) that were slightly higher than those containing an electron-withdrawing group (Cl; **6j**, **6k**, **6l**; 85%–92%), indicating that the electronic effect of the starting materials also slightly affects the yields.

### Catalyst recyclability

The reusability of Pd@PCC-**T** was further evaluated. In each case, Pd@PCC-**T** was recovered by centrifugation at the end of the reaction and reused directly, without any activation. Indeed, no significant loss of catalytic activity was observed even after six successive cycles, affording high conversions for both coupling and tandem reactions (Figs 3b and S15). At the end of the six catalytic cycles, the catalysts were analyzed by TEM, XPS and PXRD, and no apparent changes in the size, morphology and structure were detected (Figs S16 and S17). In addition, the Pd content was determined to be <300 ppb by ICP-MS analysis in the residual reaction solution after the sixth cycling experiments (see Supplementary Data for details). The small amount of Pd leaching further suggests the adequate stabilization of ultrafine PdNPs within the confined cage cavity and the strong interactions between NPs and 1,2,4-triazol-5-ylidene-based NHC sites to suppress the Pd leaching and enhance the catalyst stability.

## CONCLUSION

In summary, we presented our method to construct a soluble polytriazolium cage (PIC-**T**) with six 1,2,4-triazolium groups and a fascinating cavity capable of stabilizing PdNPs within its discrete cavities. Indeed, the strong interactions between Pd and *in-situ*-generated NHCs embedded into PIC-**T** can facilitate the nucleation and growth of PdNPs, while the well-confined cavity and stable aromatic backbone of PIC-T act as nano-vessels to protect the NPs from agglomeration. Consequently, the obtained Pd@PCC-**T** displays impressive chemical stability and excellent catalytic activity in Sonogashira coupling and the tandem reaction to synthesize benzofuran derivatives. The results confirm the crucial role of NHC sites for catalytic efficiency. We envision that MNPs@PCCs, integrating readily tunable NHC sites with a well-confined micro-environment, would be an ideal platform for advancing new outstanding heterogeneous catalysts.

## METHODS

### Synthesis of Pd@PCC-T and Pd@PCC-I

In a typical synthesis, PIC-**T** (20.7 mg, 0.008 mmol) was first dissolved in 10.0 mL CH_3_CN (colorless), and a solution of palladium acetate in CH_3_CN (18.0 mg, 0.08 mmol, red-orange, 3.0 mL) was then added with continuous stirring for 2 h. For the resulting mixture, a freshly prepared solution of NaBH_4_ (30.3 mg in 3.0 mL of methanol, 0.8 mmol) was then added dropwise and the solution color immediately changed from red-orange to deep brown. After vigorous stirring for 3 h, the mixture was evaporated to dryness to afford a dark solid after washing with water and methanol. Finally, the solid was dried under vacuum overnight to obtain Pd@PCC-**T** as a black solid powder. This method was extended to synthesize Pd@PCC-**I** by using PIC-**I** as a precursor.

### The general procedure of the catalytic Sonogashira reaction

Aryl halides (2.5 mmol), phenylacetylene derivatives (3.0 mmol), NEt_3_ (7.5 mmol, 758.9 mg), Pd@PCC-**T** (0.5 mol% Pd, 6.0 mg) and DMF (5.0 mL) were added into a 50 mL flask under air atmosphere and heated to 40°C. After completion of the reaction, distilled water was poured into the reaction mixture, which was then extracted by dichloromethane. The organic layer was then collected, washed with brine and dried over anhydrous MgSO_4_. The reaction yield was monitored by analyzing the sample with GC analysis. For examination of the reusability of Pd@PCC-**T**, the heterogeneous mixture was centrifugated after the reaction and the recovered catalyst was then reused for the subsequent reaction with fresh substrates and solvent.

### The general procedure of the catalytic tandem reaction for synthesizing 2-arylbenzofuran derivatives

A 50 mL flask was charged with Pd@PCC-**T** (0.5 mol%, 6.0 mg), 2-iodophenol derivatives (2.5 mmol), phenylacetylene derivatives (3.0 mmol), Cs_2_CO_3_ (7.5 mmol, 2.4 g) and DMSO (6.0 mL). After stirring the mixture under air atmosphere at 60^o^C for 4 h, the product was obtained through a similar process to the Sonogashira reaction and the reaction yield was then monitored by analyzing the sample with GC analysis. The reusability of Pd@PCC-**T** was continuously evaluated.

### Catalytic performance investigation into different Pd catalysts

A 50 mL Schlenk tube was charged with the Pd catalyst (0.5 mol%), 2-iodophenol (2.5 mmol), phenylacetylene (3.0 mmol), Cs_2_CO_3_ (7.5 mmol, 2.4 g) and DMSO (6.0 mL). After stirring the mixture under standard conditions, the reaction yield was monitored by analyzing the product with GC analysis.

## Supplementary Material

nwac067_Supplemental_File
