# Supplementary material for: N-heterocyclic carbene-stabilized metal nanoparticles within porous organic cages for catalytic application
Source: Natl Sci Rev. 2022 Apr 5;9(6):nwac067. doi: 10.1093/nsr/nwac067 (PMC9166563; doi:10.1093/nsr/nwac067)
Supplement: nwac067_Supplemental_File [file nwac067_supplemental_file.docx]

**Supporting Information**

**N-Heterocyclic Carbene-Stabilized Metal Nanoparticles within Porous Organic Cages for Catalytic Applications**

**Tong Liu**^1,†^**, Sha Bai**^1,†^**, Le Zhang**^1^**, F. Ekkehardt Hahn**^1,2^ **and Ying-Feng Han**^1,*^

^1^Key Laboratory of Synthetic and Natural Functional Molecule of the Ministry of Education, College of Chemistry and Materials Science, Northwest University, Xi’an 710127.

^2^Institut für Anorganicshe und Analytische Chemie Westfälische Wilhelms-Universität Münster Corrensstraße 30, Münster 48149.

*Corresponding author. E-mail: yfhan@nwu.edu.cn

^†^Equally contributed to this work.

**Table of Contents**

[1. General procedures S3](#_Toc92787622)

[2. Synthesis of PIC-**T** S4](#_Toc92787623)

[3. Catalytic properties of Pd@PCC-**T** S9](#_Toc92787624)

[4. Characterization of Pd@PCC-**T** and Pd@PCC-**I** S21](#_Toc92787625)

[5. Stability test of Pd@PCC-**T** S26](#_Toc92787626)

[6. Recyclability test of Pd@PCC-**T** S27](#_Toc92787627)

[7. Selected NMR and MS spectra for compounds S37](#_Toc92787628)

[8. References S75](#_Toc92787629)

# 1. General procedures

^1^H and ^13^C{^1^H} NMR spectra were recorded at 298 K on Bruker AVANCE III 400/600 spectrometers. Chemical shifts (*δ*) are expressed in ppm downfield from tetramethyl-silane using the residual protonated solvent as an internal standard. Mass spectra were achieved with Bruker microTOF-Q II mass spectrometer in the electrospray ionization (ESI) mode. Powder X-ray diffraction (PXRD) was performed with an X-ray diffractometer (D8 ADVANCE*, Bruker, German) with Cu Kα radiation (*λ* = 1.5418 Å). Field emission scanning electron microscopy (FE-SEM) was carried out on a Hitachi SU8010 instrument with an energy-dispersive X-ray spectrometer (EDS). Transmission electron microscopy (TEM) images were measured on Talos F200X (Thermo Scientific) operating at 200 kV, the samples were prepared by drop casting the colloidal solution on the carbon coated copper TEM grids (300 mesh) from Electron Microscopy Sciences. X-ray photoelectron spectroscopy (XPS) measurements were carried out on a PHI-5000 (Ulvac-Phi, Japan) multifunctional spectrometer using Al Kα radiation, the C1*s* peak was calibrated at 284.8 eV. Inductively coupled plasma mass spectrometry (ICP-MS) was performed on an Agilent 7900 instrument.

# 2. Synthesis of PIC-T

For the synthetic strategy of metal-carbene template approach (MCTA) used here, the template metals (pink) are silver(I) ions, the functional groups (red) are olefin units attached to the terminal ends of the trigonal tristriazolium ligand precursor **A** and the coordination sites (blue) are triazolium (in **A**) or (after triple C_NHC_-deprotonation) 1,2,4-triazolin-5-ylidene moieties (in **B**) attached to the central trigonal building block. Upon the formation of the cylinder-like trisilver-hexacarbene complex **B**, the six olefin pendants are arranged in three pairs, which react in photochemical [2+2] cycloaddition reactions upon post-synthetic irradiation to give the closed-cage complex **C**. In the final step, removal of the silver(I) metal templates yields polytriazolium cage (**D**).


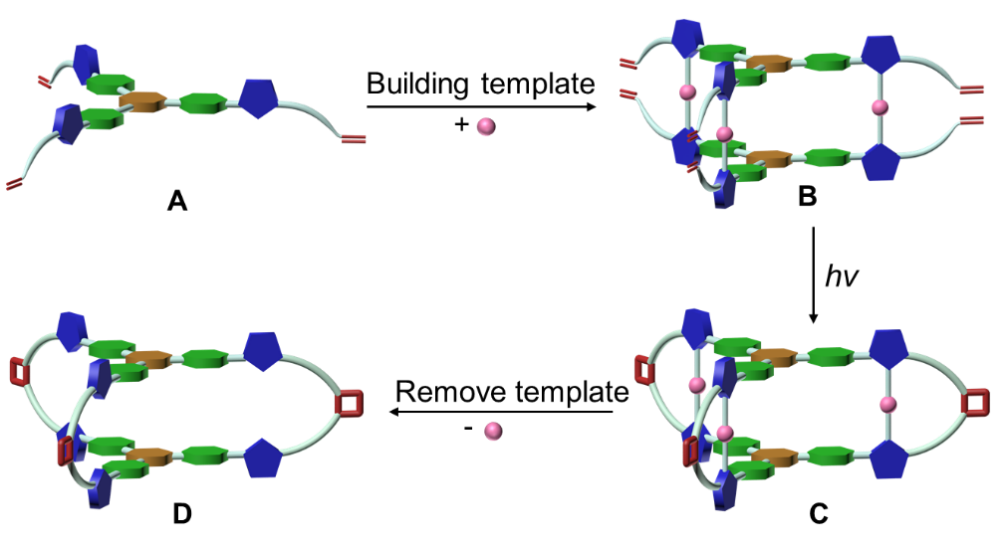


**Figure S1**. The illustration of the preparation of polytriazolium cage (PIC-**T**) by metal-carbene template approach (MCTA).

**Scheme S1.** General synthesis of polytriazolium cage PIC-**T**.

**Synthesis of H_3_-1(BF_4_)_3_**

A Schlenk flask was charged with methyl 3-(4-(1*H*-1,2,4-triazol-1-yl)phenyl)acrylate (687.7 mg, 3.0 mmol) and 2,4,6 tris(4-(bromomethyl)phenyl)-1,3,5-triazine (588.1 mg, 1.0 mmol). To this mixture was added DMF (10.0 mL), and the reaction mixture was heated to 110 °C for 24 h. During this time a white compound precipitated, which was filtered off, washed with diethyl ether, and dried *in vacuo* to give a white solid. The obtained solid was transferred to a bottle containing 80.0 mL methanol. Upon addition of a solution of NH_4_BF_4_ (346.0 mg, 3.3 mmol) in methanol (8.0 mL) to this solution, the white tetrafluoroborate salt H_3_-**1**(BF_4_)_3_ precipitated immediately. The precipitated solid was collected by filtration, washed with small portions of cold methanol and diethyl ether, and dried *in vacuo*. Yield: 1.166 g (0.9 mmol, 90%, two steps). ^1^H NMR (400 MHz, DMSO-*d*_6_): *δ* = 11.11 (s, 3H, H_i_), 9.81 (s, 3H, H_j_), 8.82 (d, *J* = 8.0 Hz, 6H, H_n_), 8.08 (d, *J* = 8.0 Hz, 6H, H_g_), 8.00 (d, *J* = 8.0 Hz, 6H, H_f_), 7.86 (d, *J* = 8.0 Hz, 6H, H_m_), 7.76 (d, *J* = 16.0 Hz, 6H, H_d_), 6.84 (d, *J* = 16.0 Hz, 3H, H_c_), 5.78 (s, 6H, H_k_), 3.77 (s, 9H, H_a_). ^13^C{^1^H} NMR (100 MHz, DMSO-*d*_6_): *δ* = 170.9 (C_p_), 166.4 (C_b_), 145.2 (C_i_ + C_j_), 142.6 (C_d_), 142.2 (C_h_), 138.1 (C_l_), 136.1 (C_e_), 135.9 (C_o_), 130.0 (C_f_), 129.6 (C_n_), 129.4 (C_m_), 121.1 (C_g_), 120.2 (C_c_), 51.7 (C_k_), 50.8 (C_a_). IR data (KBr pellet, cm^-1^): 1710 s (C=O), 1635 m (R'HC=CHR), 1570 s (C=C + C=N), 601 w (C-H in 1,2,4-trazolium units). HRMS (ESI, positive ions): *m/z* = 345.1344 (calcd. for [H_3_-**1**]^3+^ 345.1346) and *m/z* = 561.2034 (calcd. for [H_3_-**1**(BF_4_)]^2+^ 561.2041).

**Synthesis of [Ag_3_(1)_2_](BF_4_)_3_**

A sample of H_3_-**1**(BF_4_)_3_ (64.8 mg, 0.05 mmol) was dissolved in 40.0 mL of CH_3_CN, and to this solution was added Ag_2_O (34.7 mg, 0.15 mmol). The resulting suspension was heated to 65 °C for 24 h under exclusion of light. After cooling to ambient temperature, the obtained suspension was filtered slowly through Celite to obtain a clear solution. The filtrate was concentrated to 2.0 mL, and diethyl ether (20.0 mL) was added. This led to the precipitation of a white solid. The solid was collected by filtration, washed with diethyl ether, and dried *in vacuo*. Yield: 61.0 mg (0.023 mmol, 91%). ^1^H NMR (400 MHz, DMSO-*d_6_*): *δ* = 9.24 (s, 6H, H_j_), 8.21 (d, *J* = 8.2 Hz, 12H, H_n_), 7.98 (d, *J* = 8.4 Hz, 12H, H_g_), 7.76 (d, *J* = 8.4 Hz, 12H, H_f_), 7.65 (d, *J* = 16.0 Hz, 6H, H_d_), 7.37 (d, *J* = 8.2 Hz, 12H, H_m_), 6.65 (d, *J* = 16.0 Hz, 6H, H_c_), 5.72 (s, 12H, H_k_), 3.76 (s, 18H, H_a_). ^13^C{^1^H} NMR (100 MHz, DMSO-*d_6_*): *δ* = 181.3 (C_i_), 169.9 (C_p_), 166.3 (C_b_), 145.6 (C_j_), 142.7 (C_d_), 140.4 (C_l_), 140.1 (C_h_), 134.8 (C_e_), 134.7 (C_o_), 129.5 (C_f_), 128.8 (C_n_), 127.5 (C_m_), 123.2 (C_g_), 119.4 (C_c_), 51.8 (C_k_), 51.6 (C_a_). IR data (KBr pellet, cm^-1^): 1700 s (C=O), 1630 m (R'HC=CHR), 1570 s (C=C + C=N). HRMS (ESI, positive ions): *m/z* = 796.5014 (calcd. for [Ag_3_(**1**)_2_]^3+^ 796.4925).

**Synthesis of [Ag_3_(2)](BF_4_)_3_**

A CH_3_CN solution (30.0 mL) of [Ag_3_(**1**)_2_](BF_4_)_3_ (26.5 mg, 0.01 mmol) in 50 mL quartz tube was irradiated with a Philips mercury high-pressure lamp (*λ* = 365 nm ) at ambient temperature for 24 h. During this time the initially colorless solution turned brown. The conversion to [Ag_3_(**2**)](BF_4_)_3_ was quantitative. ^1^H NMR (400 MHz, DMSO-*d*_6_): *δ* = 9.14 (s, 6H, H_j_), 8.22 (d, *J* = 8.0 Hz, 12H, H_n_), 7.41-6.95 (m, 36H, H_f_ + H_m_ + H_g_) [Due to overlapping of other resonances, one of the benzene proton signals can not well be resolved], 5.68 (s, 12H, H_k_), 4.43 (d, *J* = 5.3 Hz, 6H, H_d_), 4.21 (d, *J* = 5.3 Hz, 6H, H_c_), 3.71 (s, 18H, H_a_). ^13^C{^1^H} NMR (100 MHz, DMSO-*d_6_*): *δ* = 182.1 (C_i_), 172.6 (C_b_), 170.1 (C_p_), 145.3 (C_j_), 140.7 (C_l_), 140.1 (C_e_), 137.8 (C_h_), 134.8 (C_o_), 128.8 (C_n_), 127.7 (C_f_), 124.5 (C_m_), 52.0 (C_a_), 51.2 (C_k_), 44.9 (C_d_), 40.2 (C_c_). IR data (KBr pellet, cm^-1^): 1720 s (C=O), 1570 s (C=C + C=N). HRMS (ESI, positive ions): *m/z* = 796.5119 (calcd. for [Ag_3_(**2**)]^3+^ 796.4925) and *m/z* = 1238.2002 (calcd. for [Ag_3_(**2**)(BF_4_)]^2+^ 1238.2406).

**Synthesis of PIC-T**

A sample of [Ag_3_(**2**)](BF_4_)_3_ (79.5 mg, 0.03 mmol) was dissolved in a mixture of CH_3_OH (40.0 mL) and to this solution was added NH_4_Cl (9.6 mg, 0.18 mmol). The white solid (AgCl) precipitated immediately. After stirred for 2 h, the resulting suspension was filtered through Celite to obtain a clear solution and the solvent was removed then to give a white solid. This white solid was subsequently dissolved in CH_3_OH (20.0 mL) and a solution of NH_4_BF_4_ (18.9 mg, 0.18 mmol) in methanol (3.0 mL) was added. Finally, the mixture was stirred at ambient temperature for 2 h. After this period, a white solid precipitated, which was isolated by filtration, washed with diethyl ether and dried in vacuo. Yield: 51.8 mg (0.02 mmol, 76%). ^1^H NMR (600 MHz, CD_3_CN): *δ* = 10.00 (s, 1H, H_i_), 8.86 (s, 1H, H_j_), 8.35 (d, *J* = 12.0 Hz, 2H, H_n_), 7.59 (d, *J* = 6.0 Hz, 2H, H_g_), 7.56 (d, *J* = 12.0 Hz, 2H, H_m_), 7.31 (d, *J* = 6.0 Hz, 2H, H_f_), 5.59 (s, 2H, H_k_), 4.57 (d, *J* = 6.0 Hz, 1H, H_d_), 4.09 (d, *J* = 6.0 Hz, 1H, H_c_), 3.74 (s, 3H, H_a_). ^13^C{^1^H} NMR (150 MHz, CD_3_CN) *δ* = 173.5 (C_b_), 171.4 (C_p_), 145.6 (C_j_), 142.8 (C_e_), 141.3 (C_i_), 137.9 (C_l_), 137.1 (C_o_), 134.2 (C_h_), 130.6 (C_f_), 130.4 (C_n_), 130.0 (C_m_), 121.4 (C_g_), 52.8 (C_a_), 52.3 (C_k_), 45.6 (C_d_), 42.6 (C_c_). IR data (KBr pellet, cm^-1^): 1700 s (C=O), 1570 s (C=C + C=N), 601 w (C-H in 1,2,4-trazolium units). HRMS (ESI, positive ions): *m/z* = 431.7600 (calcd. for [(PIC-**T)** - 5(BF_4_)]^5+^ 431.7630), *m/z* = 561.4525 (calcd. for [(PIC-**T**) - 4(BF_4_)]^4+^ 561.4549), *m/z* = 777.2676 (calcd. for [(PIC-**T**) - 3(BF_4_)]^3+^ 777.2730) and *m/z* = 1209.4016 (calcd. for [(PIC-**T**) - 2(BF_4_)]^2+^ 1209.4140).


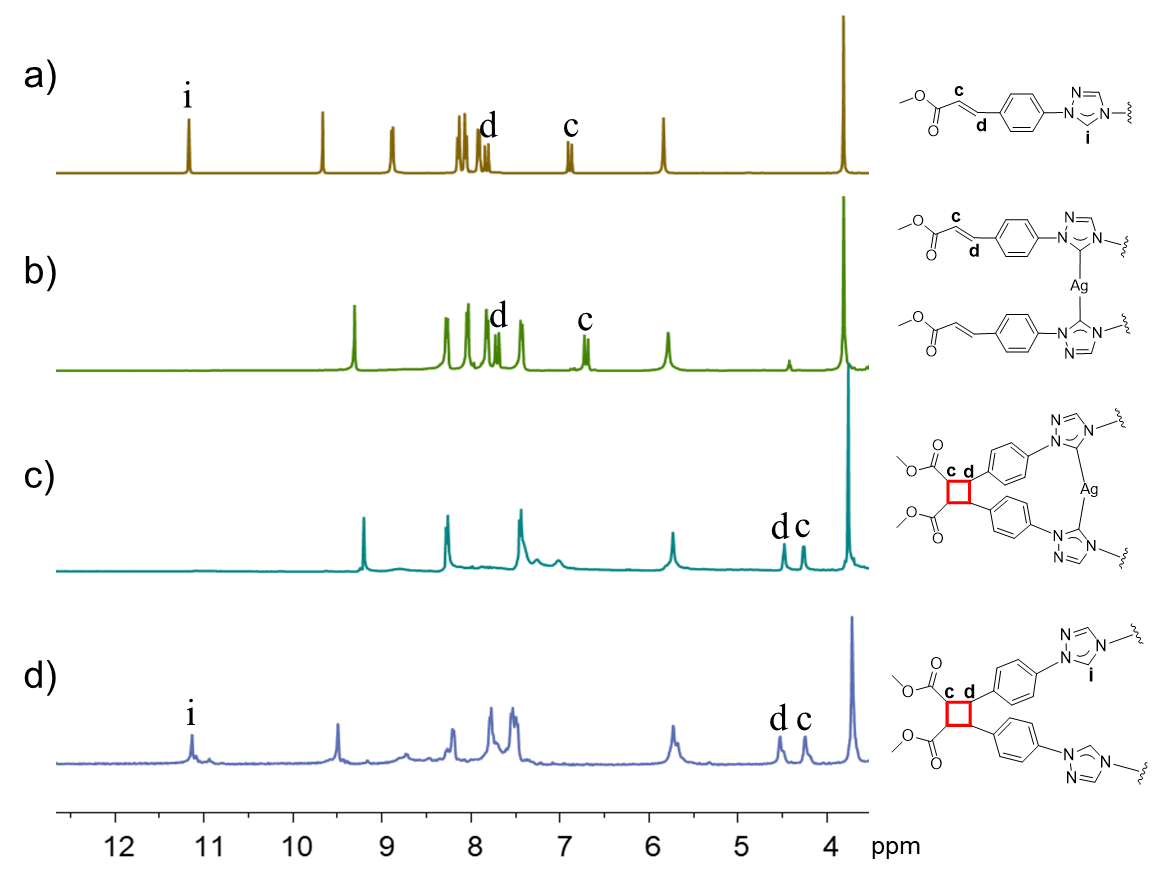


**Figure S2**. Sections of the ^1^H NMR spectra (400 MHz, 298 K, DMSO-*d*_6_): a) H_3_-**1**(BF_4_)_3_; b) [Ag_3_(**1**)_2_](BF_4_)_3_; c) [Ag_3_(**2**)](BF_4_)_3_ (after UV irradiation of [Ag_3_(**1**)_2_](BF_4_)_3_; d) PIC-**T**.

# 3. Catalytic properties of Pd@PCC-T

**3.1 General procedure for the Sonogashira reaction using Pd@PCC-T as catalyst**

In order to optimize reaction conditions, a variety of parameters such as solvent, base, temperature and the amount of catalyst in the catalytic Sonogashira reaction were measured. Firstly, bromobenzene (2.5 mmol, 392.5 mg), phenylacetylene (3.0 mmol, 306.4 mg), base (3 equiv., 7.5 mmol) and corresponding amount of Pd@PCC-**T** were added into a 50 mL flask under the air atmosphere, then the solvent (5.0 mL) was dropped into the mixture at corresponding reaction temperature. All the course of the reaction was monitored using TLC. After completion of the reaction, distilled water was poured into the reaction mixture which was then extracted by dichloromethane. The organic layer was then collected, washed with brine and dried over anhydrous MgSO_4_. The reaction yield was monitored by analyzing the sample with GC analysis.

**Table S1**. Reaction parameters optimization for the Sonogashira coupling^a^.

| Entry | n (mol %) | Temp. (^o^C) | Time (h) | Base | Solvent | Yield (%)^b^ |
| --- | --- | --- | --- | --- | --- | --- |
| 1 | 0.5 | 40 | 6 | K_2_CO_3_ | DMF | 89 |
| 2 | 0.5 | 40 | 6 | K_2_CO_3_ | DMSO | 79 |
| 3 | 0.5 | 40 | 6 | K_2_CO_3_ | CH_3_CN | 63 |
| 4 | 0.5 | 40 | 6 | K_2_CO_3_ | THF | trace |
| 5 | 0.5 | 40 | 6 | K_2_CO_3_ | H_2_O | trace |
| 6 | 0.5 | 40 | 6 | K_2_CO_3_ | CH_3_OH | 33 |
| 7 | 0.5 | 40 | 6 | NEt_3_ | DMF | 99 |
| 8 | 0.5 | 40 | 6 | K_3_PO_4_ | DMF | trace |
| 9 | 0.5 | 40 | 6 | *t*-BuOK | DMF | 55 |
| 10 | 0.5 | 40 | 6 | Cs_2_CO_3_ | DMF | 70 |
| 11 | 0.5 | 30 | 10 | NEt_3_ | DMF | 90 |
| 12 | 0.5 | 40 | 5 | NEt_3_ | DMF | 92 |
| 13 | 0.25 | 40 | 6 | NEt_3_ | DMF | 71 |
| 14^c^ | 0 | 40 | 6 | NEt_3_ | DMF | trace |

^a^ Reaction conditions: bromobenzene (2.5 mmol), phenylacetylene (3.0 mmol), base (7.5 mmol), solvent (5.0 mL) and Pd@PCC-**T** (n mol%). ^b^ Yield determined by GC analysis. ^c^ The PIC-**T** was directly used as the catalyst for a contrast experiment.

With the optimized reaction conditions in hand (NEt_3_, 0.5 mol% Pd@PCC-**T**, DMF, 40 °C), the feasibility of Pd@PCC-**T** was investigated by using wide varieties of electronical aryl halides with phenylacetylene derivatives. Similarly, aryl halides (2.5 mmol), phenylacetylene derivatives (3.0 mmol), NEt_3_ (7.5 mmol, 758.9 mg) and Pd@PCC-**T** (0.5 mol % Pd, 6.0 mg) were added into a 50.0 mL flask under the air atmosphere, then DMF (5.0 mL) was dropped into the mixture at 40 °C. After completion of the reaction, distilled water was poured into the reaction mixture which was then extracted by dichloromethane. The organic layer was then collected, washed with brine and dried over anhydrous MgSO_4_. The reaction yield was monitored by analyzing the sample with GC analysis. All the products were characterized by ^1^H NMR.

To evaluate the catalytic performance of the as-prepared Pd@PCC-**T** material, we employed a series of conventionally available catalysts in the Sonogashira coupling of bromobenzene and phenylacetylene for comparison. Under the same reaction conditions, PdCl_2_, Pd(OAc)_2_, Pd/C, and PdCl_2_(PPh_3_)_2_ only afforded trace product, and the combination of CuI and PdCl_2_(PPh_3_)_2_ gave 43% isolated yield. Furthermore, the Pd@PCC-**T** exhibits a comparative catalytic performance in comparison with a series of NHC-Pd(II) complexes and Pd nanoparticles, even under lower temperature and shorter time [1,2]. These results clearly illustrated the critical role of confined space in enhancing catalytic efficiency in the Sonogashira coupling.

**Table S2.** The Sonogashira coupling for the synthesis of catalyzed by different Pd catalysts^a^.

| Entry | Catalyst | Co-Catalyst | Yield (%)^b^ |
| --- | --- | --- | --- |
| 1 | Pd@PCC-**T** | / | 99 |
| 2 | PdCl_2_ | / | 11 |
| 3 | Pd(OAc)_2_ |  | 15 |
| 4 | Pd/C | / | trace |
| 5 | [Ph_3_P]_2_PdCl_2_ | / | trace |
| 6 | [Ph_3_P]_2_PdCl_2_ | CuI | 43 |

^a^ Reaction conditions: bromobenzene (2.5 mmol), phenylacetylene (3.0 mmol), NEt_3_ (7.5 mmol), DMF (5.0 mL) and Pd catalyst (0.5 mol% Pd). ^b^ Yield determined by GC analysis.

**NMR data for the coupling products**

**3a**. 1,2-diphenylethyne

Purification was performed by preparative thin layer chromatography plate using petroleum ether as eluent and finally gave white solid (isolated yield: 95%, 423.3 mg). ^1^H NMR (400 MHz, CDCl_3_): *δ* = 7.59-7.56 (m, 4H), 7.40-7.35 (m, 6H). ^13^C{^1^H} NMR (100 MHz, CDCl_3_): *δ* = 131.7, 128.5, 128.4, 123.4, 89.5 [3].

**3b**. 1-methyl-4-(phenylethynyl)benzene

Purification was performed by preparative thin layer chromatography plate using petroleum ether as eluent and finally gave white solid (isolated yield: 93%, 447.0 mg). ^1^H NMR (400 MHz, CDCl_3_): *δ* = 7.55 (d, *J* = 6.0 Hz, 2H), 7.45 (d, *J* = 8.0 Hz, 2H), 7.36-7.34 (m, 3H), 7.17 (d, *J* = 7.8 Hz, 2H), 2.38 (s, 3H). ^13^C{^1^H} NMR (100 MHz, CDCl_3_): *δ* = 138.5, 131.7, 131.6, 129.2, 128.4, 128.2, 123.6, 120.3, 89.7, 88.8, 21.6 [3].

**3c**. 1-methoxy-4-(phenylethynyl)benzene

Purification was performed by preparative thin layer chromatography plate using petroleum ether as eluent and finally gave white solid (isolated yield: 97%, 505.0 mg). ^1^H NMR (400 MHz, CDCl_3_): *δ* = 7.54-7.48 (m, 4H), 7.37-7.33 (m, 3H), 6.89 (d, *J* = 8.7 Hz, 2H), 3.83 (s, 3H). ^13^C{^1^H} NMR (100 MHz, CDCl_3_): *δ* = 159.7, 133.2, 131.6, 128.5, 128.1, 123.7, 115.5, 114.1, 89.6, 88.2, 55.4 [4].

**3d**. 1-fluoro-4-(phenylethynyl)benzene

Purification was performed by preparative thin layer chromatography plate using petroleum ether as eluent and gave colorless liquid (isolated yield: 82%, 402.3 mg). ^1^H NMR (400 MHz, CDCl_3_): *δ* = 7.54-7.49 (m, 4H), 7.37-7.34 (m, 3H), 7.05 (t, *J* = 8.3 Hz, 2H). ^13^C{^1^H} NMR (100 MHz, CDCl_3_): *δ* = 162.6 (d, *J*_C-F_ = 249.5 Hz), 133.6 (d, *J*_C-F_ = 8.3 Hz), 131.7, 128.5, 128.5, 123.2, 119.5 (d, *J*_C-F_ = 3.4 Hz), 115.8 (d, *J*_C-F_ = 22.1 Hz), 89.2, 88.4 [4].

**3e**. 1-chloro-4-(phenylethynyl)benzene

Purification was performed by preparative thin layer chromatography plate using petroleum ether as eluent and finally gave white solid (isolated yield: 87%, 462.6 mg). ^1^H NMR (400 MHz, CDCl_3_): *δ* = 7.54-7.51 (m, 2H), 7.46 (d, *J* = 8.0 Hz, 2H), 7.38-7.30 (m, 5H). ^13^C{^1^H} NMR (100 MHz, CDCl_3_): *δ* = 134.4, 132.9, 131.7, 128.8, 128.6, 128.5, 123.0, 121.9, 90.4, 88.4 [4].

**3f**. 1-(phenylethynyl)-4-(trifluoromethyl)benzene

Purification was performed by preparative thin layer chromatography plate using petroleum ether as eluent and finally gave white solid (isolated yield: 85%, 523.2 mg). ^1^H NMR (400 MHz, CDCl_3_): *δ* = 7.65-7.59 (m, 4H), 7.56-7.53 (m, 2H), 7.39-7.36 (m, 3H). ^13^C{^1^H} NMR (150 MHz, CDCl_3_): *δ* = 131.82, 131.77, 129.9 (q, *J*_C-F_ = 32.7 Hz), 128.8, 128.5, 127.1 (d, *J*_C-F_ = 1.6 Hz), 125.3 (q, *J*_C-F_ = 3.8 Hz), 124.0 (q, *J*_C-F_ = 272.1 Hz), 122.6, 91.8, 88.0 [3].

**3g**. 1-(tert-butyl)-4-(phenylethynyl)benzene

Purification was performed by preparative thin layer chromatography plate using petroleum ether as eluent and finally gave white solid (isolated yield: 93%, 544.8 mg). ^1^H NMR (400 MHz, CDCl_3_): *δ* = 7.54-7.51 (m, 2H), 7.48 (t, *J =* 6.0 Hz, 1H), 7.46 (t, *J =* 2.0 Hz, 1H), 7.39-7.31(m, 5H), 1.33 (s, 9H). ^13^C{^1^H} NMR (100 MHz, CDCl_3_): *δ* = 151.6, 131.7, 131.4, 128.4, 128.2, 125.5, 123.6, 120.3, 89.6, 88.8, 34.9, 31.3 [3].

**3h**. 4-(phenylethynyl)pyridine

Purification was performed by preparative thin layer chromatography plate using petroleum ether as eluent and finally gave white solid (isolated yield: 82%, 367.4 mg). ^1^H NMR (400 MHz, CDCl_3_): *δ* = 8.63 (d, *J* = 2.4 Hz, 2H), 7.57 (d, *J* = 4.4 Hz, 2H), 7.5 (d, *J* = 4.0 Hz, 2H), 7.43-7.38 (m, 3H). ^13^C{^1^H} NMR (100 MHz, CDCl_3_): *δ* = 149.5, 131.9, 131.8, 129.3, 128.5, 125.6, 122.0, 94.3, 86.6 [5].

**3i**. 1-fluoro-3-(phenylethynyl)benzene

Purification was performed by preparative thin layer chromatography plate using petroleum ether as eluent and finally gave white solid (isolated yield: 85%, 416.9 mg). ^1^H NMR (400 MHz, CDCl_3_): *δ* = 7.54-7.52 (m, 2H), 7.37-7.33 (m, 3H), 7.31-7.29 (m, 2H), 7.24-7.21 (m, 1H), 7.07-7.01 (m, 1H). ^13^C{^1^H} NMR (100 MHz, CDCl_3_): *δ* = 162.5 (d, *J*_C-F_ = 246.4 Hz), 131.8, 130.1 (d, *J*_C-F_ = 8.6 Hz), 128.7, 128.5, 127.6 (d, *J*_C-F_ = 2.5 Hz), 125.3 (d, *J*_C-F_ = 9.6 Hz), 122.9, 118.5 (d, *J*_C-F_ = 22.9 Hz), 115.7 (d, *J*_C-F_ = 21.2 Hz), 90.4, 88.2 [3].

**3j**. 1-methoxy-3-(phenylethynyl)benzene

Purification was performed by preparative thin layer chromatography plate using petroleum ether as eluent and finally gave yellowish liquid (isolated yield: 93%, 484.1 mg). ^1^H NMR (400 MHz, CDCl_3_): *δ* = 7.54-7.51 (m, 2H), 7.36-7.31 (m, 3H), 7.24-7.22 (m, 1H), 7.14-7.11 (m, 1H), 7.06-7.05 (m, 1H), 6.89-6.86 (m, 1H), 3.80 (s, 3H). ^13^C{^1^H} NMR (100 MHz, CDCl_3_): *δ* = 158.3, 130.6, 128.4, 127.30, 127.26, 123.2, 123.1, 122.1, 115.3, 113.9, 88.3, 88.1, 54.2 [3].

**3k**. 1,2-bis(4-methoxyphenyl)ethyne

Purification was performed by preparative thin layer chromatography plate using petroleum ether as eluent and finally gave white solid (isolated yield: 89%, 530.2 mg). ^1^H NMR (400 MHz, CDCl_3_): *δ* = 7.46 (d, *J* = 8.0 Hz, 4H), 6.85 (d, *J* = 8.0 Hz, 4H), 3.82 (s, 6H). ^13^C{^1^H} NMR (100 MHz, CDCl_3_): *δ* = 159.5, 133.0, 115.8, 114.0, 88.0, 55.4 [6].

**3l**. 1-chloro-4-((4-methoxyphenyl)ethynyl)benzene

Purification was performed by preparative thin layer chromatography plate using petroleum ether as eluent and finally gave yellow solid (isolated yield: 80%, 485.4 mg). ^1^H NMR (400 MHz, CDCl_3_): *δ* = 7.44 (d, *J* = 4.0 Hz, 2H), 7.30 (d, *J* = 4.0 Hz, 2H), 7.24 (t, *J* = 4.0 Hz, 1H), 7.10 (d, *J* = 4.0 Hz, 1H), 7.03 (s, 1H), 6.89 (d, *J* = 4.0 Hz, 1H), 3.81(s, 3H). ^13^C{^1^H} NMR (100 MHz, CDCl_3_): *δ* = 159.8, 133.9, 133.1, 132.6, 128.7, 122.1, 115.0, 114.1, 90.4, 87.0, 55.3 [6].

**3.2 General procedure for the tandem reaction using Pd@PCC-T / Pd@PCC-I as catalyst**

To obtain the optimize reaction conditions, a variety of parameters such as solvent, base, temperature and the amount of catalyst in the tandem reaction were also measured. In a 50 mL flask, 2-iodophenol (2.5 mmol, 485.6 mg), phenylacetylene (3.0 mmol, 306.4 mg), base (3 equiv., 7.5 mmol) and corresponding amount of Pd@PCC-**T** in solvent (6.0 mL) were stirred under the air atmosphere at the reaction temperature. The process was monitored by TLC. After completion of the reaction, distilled water was poured into the reaction mixture which was then extracted by dichloromethane. The organic layer was then collected, washed with brine and dried over anhydrous MgSO_4_. The reaction yield was monitored by analyzing the sample with GC analysis.

**Table S3**. Reaction parameters optimization for tandem catalysis^a^.

| Entry | n (mol %) | Temp. (^o^C) | Time (h) | Base | Solvent | Yield (%)^b^ |
| --- | --- | --- | --- | --- | --- | --- |
| 1 | 0.5 | 80 | 8 | Cs_2_CO_3_ | DMSO | 98 |
| 2 | 0.5 | 60 | 8 | Cs_2_CO_3_ | DMSO | 98 |
| 3 | 0.5 | 40 | 8 | Cs_2_CO_3_ | DMSO | 62 |
| 4 | 0.5 | 40 | 12 | Cs_2_CO_3_ | DMSO | 63 |
| 5 | 0.5 | 60 | 4 | Cs_2_CO_3_ | DMSO | 98 |
| 6 | 0.5 | 60 | 3 | Cs_2_CO_3_ | DMSO | 85 |
| 7 | 0.5 | 60 | 4 | Cs_2_CO_3_ | DMF | 77 |
| 8 | 0.5 | 60 | 4 | Cs_2_CO_3_ | CH_3_CN | 86 |
| 9 | 0.5 | 60 | 4 | Cs_2_CO_3_ | CH_3_OH | 64 |
| 10 | 0.5 | 60 | 4 | Cs_2_CO_3_ | THF | 30 |
| 11 | 0.5 | 60 | 4 | Cs_2_CO_3_ | DCM | 15 |
| 12 | 0.5 | 60 | 4 | K_2_CO_3_ | DMSO | 84 |
| 13 | 0.5 | 60 | 4 | *t*-BuOK | DMSO | 39 |
| 14 | 0.5 | 60 | 4 | NEt_3_ | DMSO | 61 |
| 15 | 0.5 | 60 | 4 | K_3_PO_4_ | DMSO | trace |
| 16 | 0.25 | 60 | 4 | Cs_2_CO_3_ | DMSO | 81 |
| 17^c^ | 0 | 60 | 4 | Cs_2_CO_3_ | DMSO | trace |

^a^ Reaction conditions: 2-iodophenol (2.5 mmol), phenylacetylene (3.0 mmol), base (7.5 mmol), solvent (6.0 mL) and Pd@PCC-**T** (n mol % Pd). ^b^ Yield determined by GC analysis. ^c^ The PIC-**T** was directly used as the catalyst for a contrast experiment.

**NMR data for benzofuran derivatives**

**6a**. 2-phenylbenzofuran

Purification was performed by preparative thin layer chromatography plate using petroleum ether as eluent and finally gave white solid (isolated yield: 94%, 456.4 mg). ^1^H NMR (400 MHz, CDCl_3_): *δ* = 7.88 (d, *J* = 7.1 Hz, 2H), 7.59 (d, *J* = 7.7 Hz, 1H), 7.53 (d, *J* = 7.9 Hz, 1H), 7.45 (t, *J* = 7.2 Hz, 1H), 7.35 (t, *J* = 8.0 Hz, 1H), 7.29 (t, *J* = 7.2 Hz, 1H), 7.23 (t, *J* = 7.2 Hz, 2H), 7.04 (s, 1H). ^13^C{^1^H} NMR (100 MHz, CDCl_3_): *δ* = 156.0, 155.0, 130.6, 129.3, 128.9, 128.7, 125.0, 124.4, 123.0, 121.0, 111.3, 101.4 [7].

**6b**. 2-(4-fluorophenyl)benzofuran

Purification was performed by preparative thin layer chromatography plate using petroleum ether as eluent and finally gave white solid (isolated yield: 90%, 477.5 mg). ^1^H NMR (400 MHz, CDCl_3_): *δ* = 7.84 (dd, *J* = 8.5, 5.3 Hz, 2H), 7.58 (d, *J* = 4.0 Hz, 1H), 7.51 (d, *J* = 4.0 Hz, 1H), 7.28 (t, *J* = 8.0 Hz 1H), 7.23 (t, *J* = 8.0 Hz, 1H), 7.14 (t, *J* = 8.0 Hz, 2H), 6.96 (s, 1H). ^13^C{^1^H} NMR (100 MHz, CDCl_3_): *δ* = 163.0 (d, *J*_C-F_ = 248.7 Hz), 155.1, 154.9, 129.3, 126.9 (d, *J*_C-F_ = 8.3 Hz), 126.9 126.8, 123.1, 121.0, 116.0 (*d*, *J*_C-F_ = 21.9 Hz), 111.2, 101.1 (d, *J*_C-F_ = 1.6 Hz) [7].

**6c**. 2-(3-fluorophenyl)benzofuran

Purification was performed by preparative thin layer chromatography plate using petroleum ether as eluent and finally gave white solid (isolated yield: 88%, 466.9 mg). ^1^H NMR (400 MHz, CDCl_3_) *δ* = 7.65-7.63 (m, 1H), 7.61-7.51 (m, 3H), 7.44-7.38 (m, 1H), 7.33-7.29 (m, 1H), 7.26-7.22 (m, 1H), 7.07-7.02 (m, 2H). ^13^C{^1^H} NMR (100 MHz, CDCl_3_): *δ* = 163.2 (d, *J*_C-F_ = 245.6 Hz), 155.0, 154.6, 132.6 (d, *J*_C-F_ = 8.7 Hz), 130.5 (d, *J*_C-F_ = 8.4 Hz), 129.0, 124.8, 123.2, 121.2, 120.7 (d, *J*_C-F_ = 2.4 Hz), 115.4 (d, *J*_C-F_ = 21.3 Hz), 111.9 (d, *J* _C-F_ = 23.4 Hz), 111.4, 102.4 [8].

**6d**. 2-(4-chlorophenyl)benzofuran

Purification was performed by preparative thin layer chromatography plate using petroleum ether as eluent and finally gave white solid (isolated yield: 87%, 497.4 mg). ^1^H NMR (400 MHz, CDCl_3_) *δ* = 7.81-7.78 (m, 2H), 7.60-7.57 (m, 1H), 7.53-7.50 (m, 1H),7.42 (dt, *J* = 11.2, 2.4 Hz, 2H), 7.32-7.28 (m, 1H), 7.26-7.22 (m, 1H), 7.02 (d, *J* = 4.0Hz, 1H). ^13^C{^1^H} NMR (100 MHz, CDCl_3_): *δ* = 155.0, 154.9, 134.4, 129.13, 129.06 (2C), 126.2, 124.7, 123.2, 121.1, 111.3, 101.9 [7].

**6e**. 2-(p-tolyl)benzofuran

Purification was performed by preparative thin layer chromatography plate using petroleum ether as eluent and finally gave white solid (isolated yield: 89%, 463.4 mg). ^1^H NMR (400 MHz, CDCl_3_) *δ* = 7.76 (d, *J* = 8.0 Hz, 2H), 7.58-7.55 (m, 1H), 7.52-7.49 (m, 1H), 7.29-7.26 (m, 2H), 7.25-7.21 (m, 2H), 6.97 (d, *J* = 0.8 Hz, 1H), 2.40 (s, 3H). ^13^C{^1^H} NMR (100 MHz, CDCl_3_): *δ* = 156.3, 154.8, 138.7, 129.6, 129.4, 127.8, 125.0, 124.1, 123.0, 120.8, 111.2, 100.7, 21.5 [7].

**6f**. 2-(4-ethylphenyl)benzofuran

Purification was performed by preparative thin layer chromatography plate using petroleum ether as eluent and finally gave white solid (isolated yield: 88%, 489.0 mg). ^1^H NMR (400 MHz, CDCl_3_) *δ* = 7.79 (dt, *J* = 8.4, 2.0 Hz, 2H), 7.58-7.56 (m, 1H), 7.46-7.43 (m, 1H), 7.30-7.27 (m, 2H), 7.25-7.16 (m, 2H), 6.98 (s, 1H), 2.70 (q, *J* = 8.0 Hz, 2H), 1.28 (t, *J* = 8.0 Hz, 3H). ^13^C{^1^H} NMR (100 MHz, CDCl_3_): *δ* = 156.3, 154.9, 145.1, 129.5, 129.4, 128.4, 128.1, 125.1, 122.9, 124.1, 120.8, 111.2, 100.7, 28.9, 15.6 [9].

**6g**. 2-(4-(trifluoromethyl)phenyl)benzofuran

Purification was performed by preparative thin layer chromatography plate using petroleum ether as eluent and finally gave white solid (isolated yield: 85%, 557.2 mg). ^1^H NMR (400 MHz, CDCl_3_) *δ* = 7.97 (d, *J* = 8.0 Hz, 2H), 7.7 (d, *J* = 8.0 Hz, 2H), 7.63-7.61 (m, 1H), 7.55 (dd, *J* = 8.0, 0.8 Hz, 1H), 7.36-7.31 (m, 1H), 7.28-7.26 (m, 1H), 7.14 (s, 1H). ^13^C{^1^H} NMR (150 MHz, CDCl_3_): *δ* =155.1, 154.2, 133.7, 130.1 (q, *J*_C-F_ = 21.6 Hz), 128.8, 125.8 (q, *J*_C-F_ = 2.6 Hz), 125.1, 125.0, 124.1 (q, *J*_C-F_ = 271.2 Hz), 123.3, 121.3, 111.4, 103.3 [10].

**6h**. 2-(4-(tert-butyl)phenyl)benzofuran

Purification was performed by preparative thin layer chromatography plate using petroleum ether as eluent and finally gave white solid (isolated yield: 90%, 563.2 mg). ^1^H NMR (400 MHz, CDCl_3_) *δ* = 7.80 (d, *J* = 8.0 Hz, 2H), 7.58-7.56 (m, 1H), 7.53-7.51 (m, 1H), 7.48 (d, *J* = 8.0 Hz, 2H), 7.29-7.27 (m, 1H), 7.25-7.20 (m, 1H), 6.98 (s, 1H), 1.36 (s, 9H). ^13^C{^1^H} NMR (100 MHz, CDCl_3_): *δ* = 156.3, 154.9, 151.9, 129.5, 127.8, 125.8, 124.8, 124.1, 122.9, 120.9, 111.2, 100.8, 34.9, 31.4 [11].

**6i**. 2-(4-methoxyphenyl)benzofuran

Purification was performed by preparative thin layer chromatography plate using petroleum ether as eluent and finally gave white solid (isolated yield: 95%, 532.6 mg). ^1^H NMR (400 MHz, CDCl_3_) *δ* = 7.80 (dt, *J* = 8.8, 2.8 Hz, 2H), 7.56-7.54 (m, 1H), 7.51-7.48 (m, 1H), 7.27-7.19 (m, 2H), 6.98 (dt, *J* = 8.8, 2.0 Hz, 2H), 6.89 (d, *J* = 0.8 Hz, 1H), 3.86 (s, 3H). ^13^C{^1^H} NMR (100 MHz, CDCl_3_): *δ* = 160.1, 156.1, 154.8, 129.6, 126.5, 123.8, 123.4, 122.9, 120.7, 114.3, 111.1, 99.8, 55.5 [7].

**6j**. 5-chloro-2-phenylbenzofuran

Purification was performed by preparative thin layer chromatography plate using petroleum ether as eluent and finally gave white solid (isolated yield: 88%, 503.1 mg). ^1^H NMR (400 MHz, CDCl_3_) *δ* = 7.85 (dt, *J* = 7.2, 1.2 Hz, 2H), 7.55 (d, *J* = 2.4 Hz, 1H), 7.48-7.42 (m, 3H), 7.40-7.36 (m, 1H), 7.24 (dd, *J* = 8.8, 2.4 Hz, 1H), 6.97 (d, *J* = 0.8Hz, 1H). ^13^C{^1^H} NMR (100 MHz, CDCl_3_): *δ* = 157.5, 153.3, 130.7, 130.0, 129.1, 129.0, 128.6, 125.1, 124.5, 120.5, 112.2, 100.9 [7].

**6k**. 5-chloro-2-(4-chlorophenyl)benzofuran

Purification was performed by preparative thin layer chromatography plate using petroleum ether as eluent and finally gave white solid (isolated yield: 80%, 526.2 mg). ^1^H NMR (400 MHz, CDCl_3_) *δ* = 7.78 (dt, *J* = 8.4, 2.4Hz, 2H), 7.55 (d, *J* = 2.0 Hz, 1H), 7.43 (dt, *J* = 8.8, 2.4 Hz, 3H), 7.25-7.23 (m, 1H), 6.95 (d, *J* = 0.8 Hz, 1H). ^13^C{^1^H} NMR (100 MHz, CDCl_3_): *δ* = 156.3, 153.3, 134.9, 130.5, 129.2, 128.7, 128.5, 126.3, 124.8, 120.6, 112.2, 101.3 [12].

**6l**. 5-chloro-2-(4-methoxyphenyl)benzofuran

Purification was performed by preparative thin layer chromatography plate using petroleum ether as eluent and finally gave white solid (isolated yield: 85%, 549.7 mg). ^1^H NMR (400 MHz, CDCl_3_) *δ* = 7.78 (dt, *J* =8.8, 3.2 Hz, 2H), 7.51 (d, *J* = 2 Hz, 1H), 7.40 (d, *J* = 8.4 Hz, 1H), 7.20 (dd, *J* = 8.8, 2.0 Hz, 1H), 6.98 (dt, *J* = 8.8, 2.8 Hz, 2H), 6.83 (d, *J* = 4Hz, 1H), 3.87 (s, 3H). ^13^C{^1^H} NMR (100 MHz, CDCl_3_): *δ* = 160.4, 157.6, 153.0, 131.0, 128.4, 126.7, 123.9, 122.9, 120.2, 114.4, 112.0, 99.3, 55.5 [12]

**6m**. 5-methyl-2-phenylbenzofuran

Purification was performed by preparative thin layer chromatography plate using petroleum ether as eluent and finally gave white solid (isolated yield: 88%, 458.2 mg). ^1^H NMR (400 MHz, CDCl_3_) *δ* = 7.85 (dt, *J* = 8.4, 1.6 Hz, 2H), 7.46-7.39 (m, 3H), 7.37-7.32 (m, 2H), 7.09 (dd, *J* = 8.0, 1.2 Hz, 1H), 6.96 (d, *J* = 1.2 Hz, 1H), 2.45 (s, 3H). ^13^C{^1^H} NMR (100 MHz, CDCl_3_): *δ* = 156.1, 153.4, 132.4, 130.7, 129.4, 128.9, 128.5, 125.6, 124.9, 120.8, 110.8, 101.2, 21.5 [8].

**6n**. 2-(4-chlorophenyl)-5-methylbenzofuran

Purification was performed by preparative thin layer chromatography plate using petroleum ether as eluent and finally gave white solid (isolated yield: 82%, 497.5 mg). ^1^H NMR (400 MHz, CDCl_3_) *δ* = 7.78 (dt, *J* = 8.4, 2.4 Hz, 2H), 7.41 (dt, *J* = 8.8, 2.4 Hz, 2H), 7.39-7.31 (m, 2H), 7.10 (dd, *J* = 8.4, 1.2 Hz, 1H), 6.94 (d, *J* = 0.8 Hz, 1H), 2.45 (s, 3H). ^13^C{^1^H} NMR (100 MHz, CDCl_3_): *δ* = 155.0, 153.4, 134.3, 132.7, 129.29, 129.26, 129.1, 126.2, 126.0, 120.9, 110.8, 101.7, 21.5 [10].

**6o**. 2-(4-chlorophenyl)-5-methylbenzofuran

Purification was performed by preparative thin layer chromatography plate using petroleum ether as eluent and finally gave white solid (isolated yield: 91%, 542.1 mg). ^1^H NMR (400 MHz, CDCl_3_): *δ* = 7.79 (td, *J* = 2.4, 9.2 Hz, 2H), 7.38 (d, *J* = 8.4 Hz, 1H), 7.34 (s, 1H), 7.06 (dd, *J* = 1.2, 7.2 Hz, 1H), 6.98 (td, *J*= 2.0, 6.8 Hz, 2H), 6.82 (s, 1 H), 3.86 (s, 3H), 2.44 (s, 3 H). ^13^C{^1^H} NMR (100 MHz, CDCl_3_): *δ* = 160.0, 156.3, 153.3, 132.3, 129.7, 126.5, 125.1, 123.6, 120.6, 114.4, 110.6, 99.6, 77.2, 55.5, 21.5 [13].

**Table S4.** TON and TOF of tandem reaction for the synthesis of benzofuran derivatives.

| Products | TON | TOF (h^-1^) |
| --- | --- | --- |
| **6a** | 19600 | 4900 |
| **6b** | 19000 | 4750 |
| **6c** | 18400 | 4600 |
| **6d** | 18400 | 4600 |
| **6e** | 19000 | 4750 |
| **6f** | 18600 | 4650 |
| **6g** | 18800 | 4700 |
| **6h** | 19400 | 4850 |
| **6i** | 19600 | 4900 |
| **6j** | 18400 | 4600 |
| **6k** | 17000 | 4250 |
| **6l** | 18400 | 4600 |
| **6m** | 18800 | 4700 |
| **6n** | 18000 | 4500 |
| **6o** | 19400 | 4850 |

# 4. Characterization of Pd@PCC-T and Pd@PCC-I


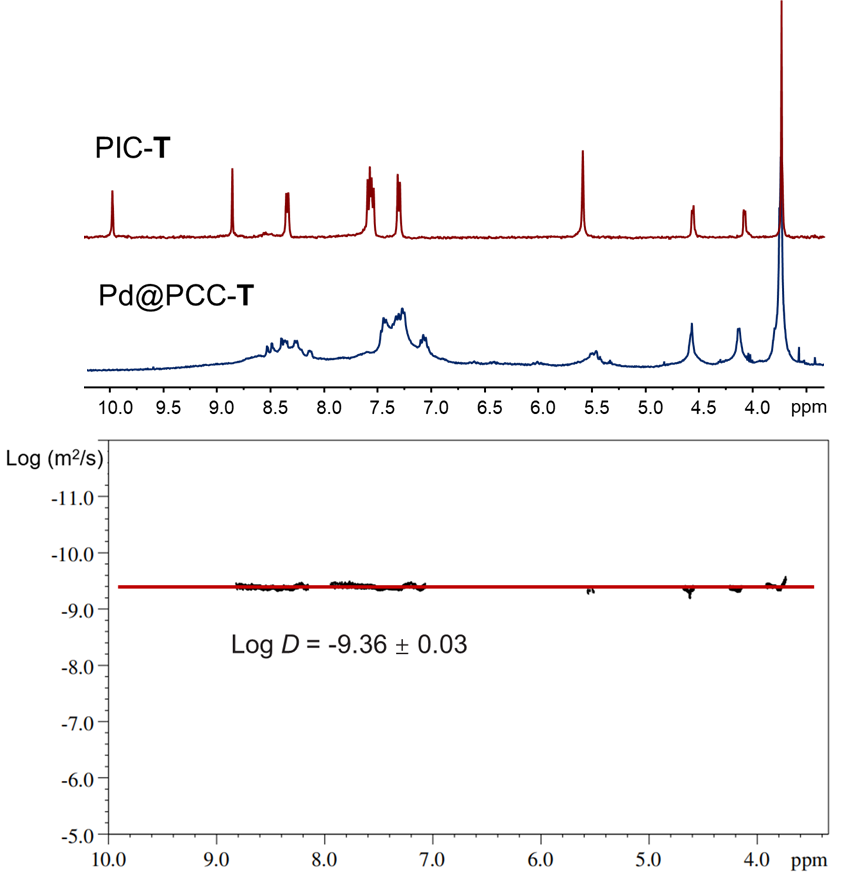


**Figure S3.** ^1^H NMR spectra (400 MHz, 298 K, CD_3_CN) of Pd@PCC-**T** and PIC-**T** and DOSY spectrum of Pd@PCC-**T**. ***Note***: The as-synthesized Pd@PCC-**T** has similar diffusion coefficient to the starting cage (Log *D* = -9.36 ± 0.01) and the reported Au@PCC-**I** (Log *D* = -9.38 ± 0.02), which confirms the similar size and shape of Pd@PCC-**T** and the starting cage or the reported Au@PCC-**I**. It offers important evidence to support that the formed PdNPs are entrapped within the cage rather than stabilized by two or more cages, or the functional groups of the cage.


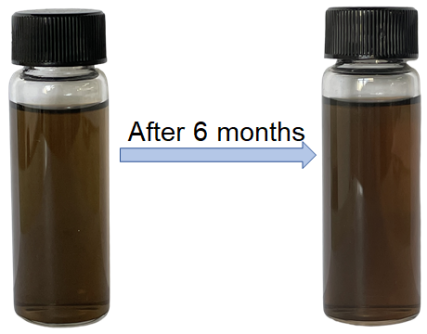


**Figure S4**. The photographs for Pd@PCC-**T** of fresh sample (left) and after 6 months (right).


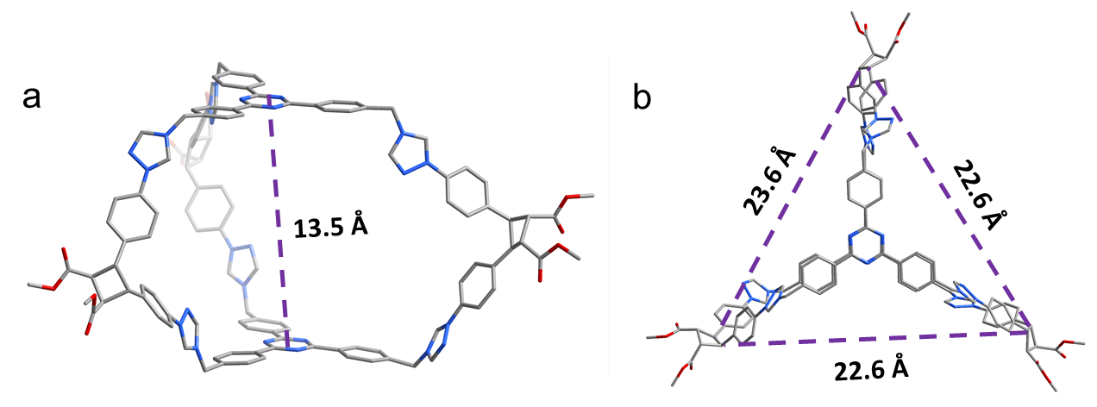


**Figure S5**. a) The side and b) top view of the molecular dimensions of Pd@PCC-**T**. The calculations of (PIC-**T**) was carried out with the Gaussian 09 Package [14]. Geometry optimization was performed with B3LYP [15] and 6-31G basis set [16]. The method of size calculation of Pd NPs was referred previous work [17]. DFT based theoretical calculations convinced that the inner cavity lengths were 2.26, 2.26, 2.36 nm and the top and bottom panels of the cage are approximately parallel in which the distance between the core of two triazine rings is 13.5 Å in the lowest energy conformer of PIC-**T**.


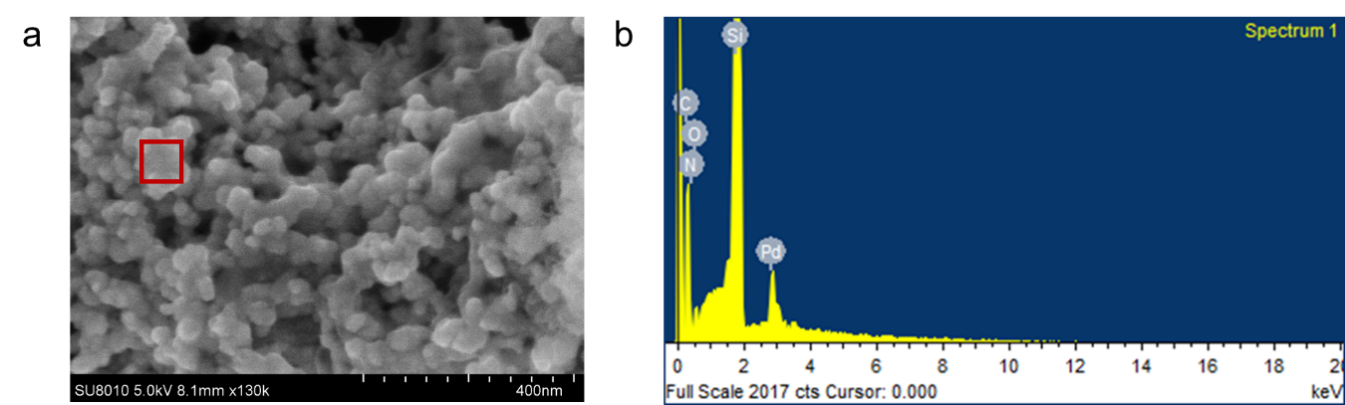


**Figure S6**. a) The SEM image and b) Energy-dispersive X-ray spectroscopic (EDS) pattern of Pd@PCC-**T**.

**Note: Detail experiment procedure for ICP-MS analysis.**

The process of determining the Pd loading of Pd@PCC-**T** by ICP-MS is as follows: 5 mg as-prepared Pd@PCC-**T** has first dissolved with nitric acid in Savillex perfluoroalkoxy polymer (PFA) vials and placed capped on a hot plate at 80 °C overnight to ensure complete dissolution. A series of standard solutions were prepared by diluting Pd(OAc)_2_ in deionized for different mass concentrations. After dissolution, the solution was filtered to remove any undissolved materials. The filtrate was diluted to 1μg**^.^**mL^−1^ with distilled water then tested upon ICP-MS to acquire the mass percentage of Palladium in 5 mg Pd@PCC-**T** [18].


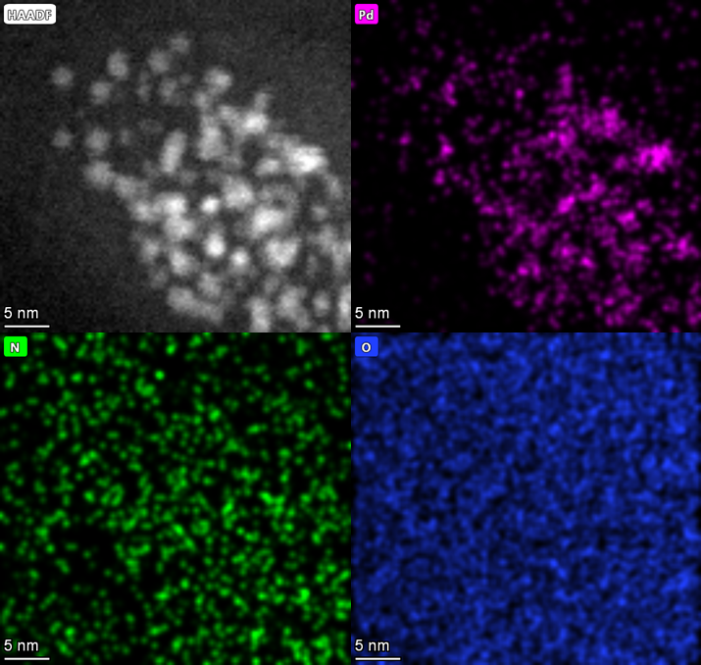


**Figure S7**. Energy dispersive X-ray elemental mapping of as-prepared Pd@PCC-**T**.


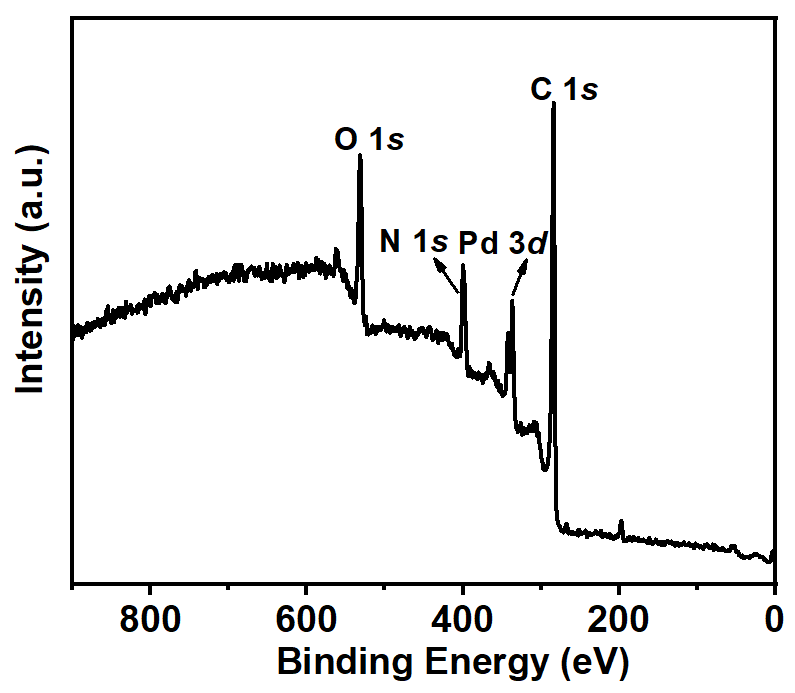


**Figure S8**. The survey XPS spectrum of Pd@PCC-**T**.


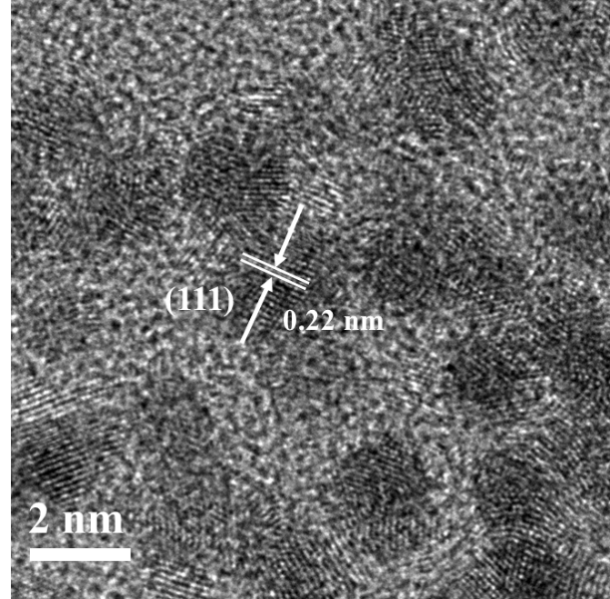


**Figure S9**. The High-resolution TEM (HRTEM) image of Pd@PCC-**T**.


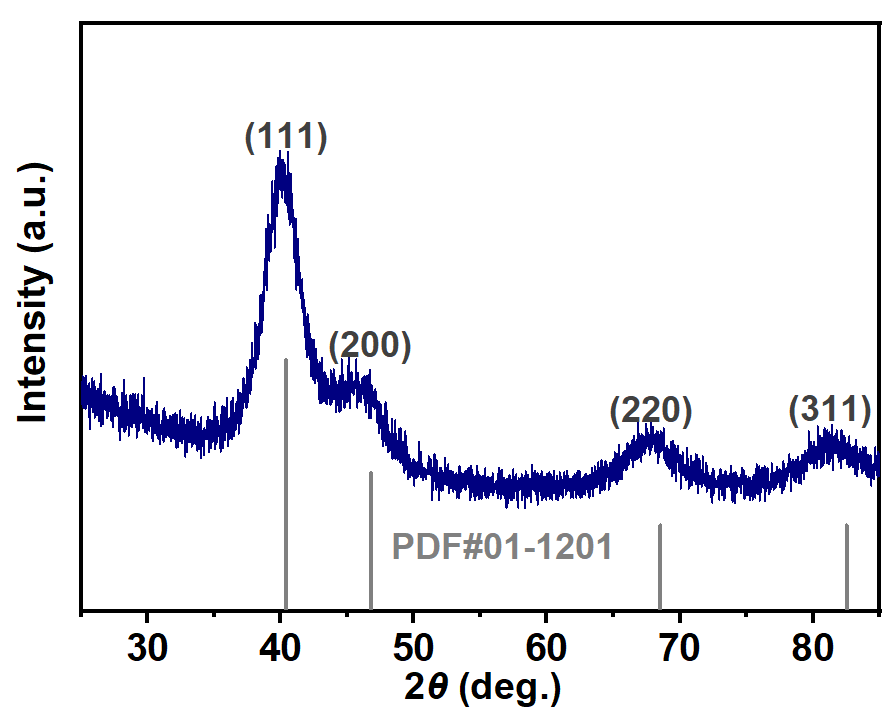


**Figure S10**. PXRD pattern of Pd@PCC-**T**.


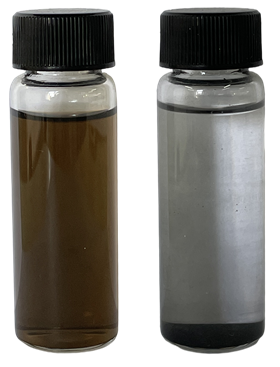


**Figure S11**. Observable optical changes of as synthesized Pd NPs (left) in presence of PIC-**T** and in absence of PIC-**T** (right).

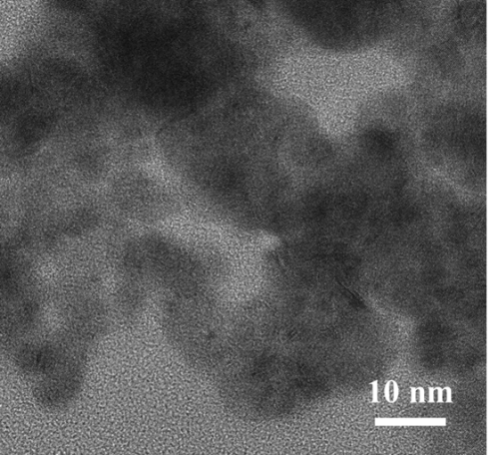


**Figure S12**. 1,2,4-triazolium-based NHC (1,4-dibutyl-1*H*-1,2,4-triazol-4-ium bromide) was chosen to protect PdNPs via the same process. The monodentate NHC (2.1 mg, 0.008 mmol) was dissolved with Pd(OAc)_2_ (2.9 mg, 0.013mmol) in 20.0 mL CH_3_CN stirring for 2 h. Then NaBH_4_ (4.9 mg in 3.0 mL of methanol, 0.13mmol) was added to solvent in order to get the reduction product. Under similar conditions to those used to form Pd@PCC-**T**, the immediate and complete precipitation of aggregated black powder was observed. TEM images showed that the finally product formed aggregated amorphous Pd(0) instead of nanoparticles, further demonstrated the crucial role of the cavity confining effect in the nucleation, controlled synthesis, and stabilization of PdNPs.


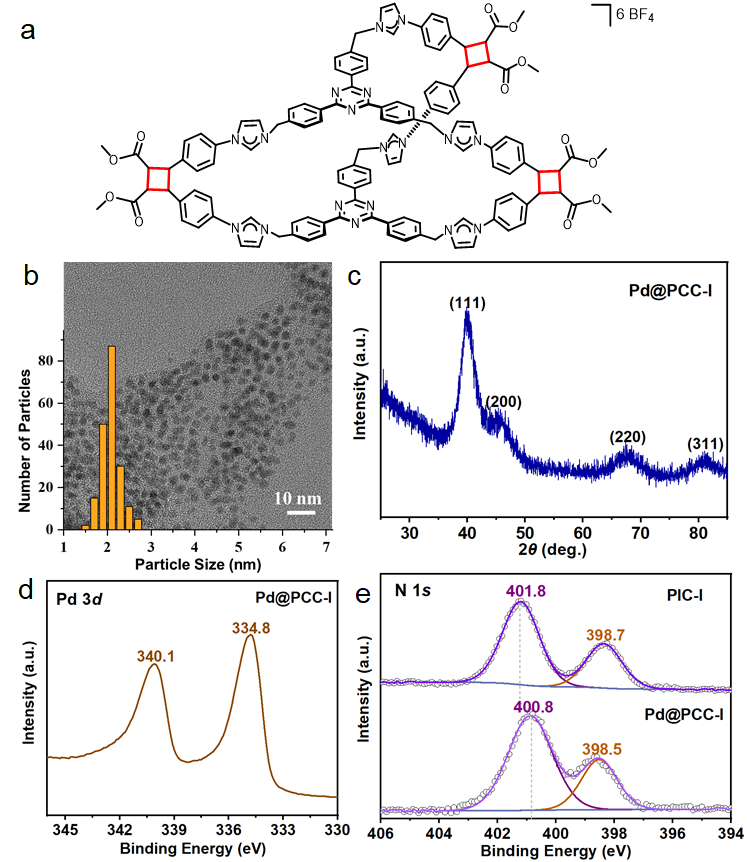


**Figure S13**. a) The chemical formula of PIC. b) TEM images confirmed the grain diameter at 2.06 ± 0.01 nm of Pd@PCC-**I**, which were similar to the size of Pd@PCC-**T**. c) The PXRD patterns of Pd@PCC-**I** displayed similar Pd fcc structures and the palladium loading was about 21 wt% which was further evaluated by ICP-MS. d) XPS Pd 3*d* proved the Pd(II) was completely reduced and e) XPS N 1*s* then proved the carbene bond formed successfully on the metal surface.

# 5. Stability test of Pd@PCC-T

To estimate the thiols etching stability of palladium nanoparticles coated with PIC-**T**, a solution of Pd@PCC-**T** in acetonitrile (3.0 mL) was treated with 5 mM dodecanethiol in methanol (100 mM, 150 μL) at ambient temperature for 24 h. Later, the sample was washed with ethanol and then dried under vacuum overnight. TEM image, XPS measurement and PXRD pattern of Pd@PCC-**T** were studied after the thiol treatment by removing solvents in vacuo. The grain diameter of Pd@PCC-**T** maintained ~2.0 nm and well dispersed (Figure S13a). Besides, nanoparticles formed from NHC shell revealed no change by N 1*s* signal and PXRD analysis (Figure S13b, c), possibly owing to the stabilization provided by the NHC-M bonds and the good confinement of PdNPs within the discrete cage.


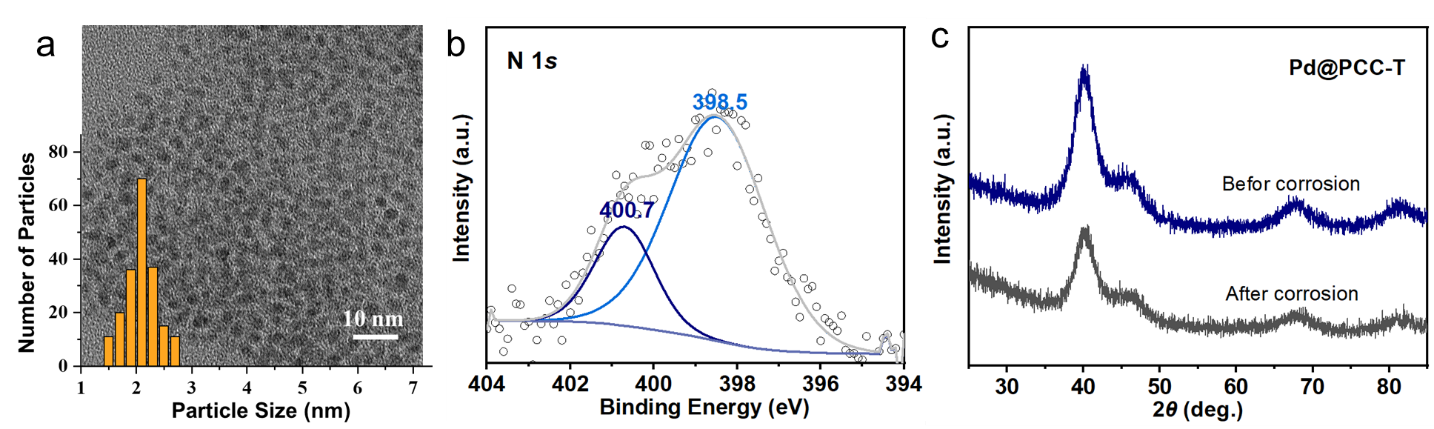


**Figure S14**. a) TEM, particle size distribution diagram, b) N 1*s* XPS spectrometry and c) PXRD of Pd@PCC-**T** after treatment with 5 mM 1-dodecanethiol (DDT) for 24 h.

# 6. Recyclability test of Pd@PCC-T

For practical purposes, the reusability of the catalyst Pd@PCC-**T** in both two reactions were investigated. After reaction, the Pd@PCC-**T** in the model reaction could be readily collected by centrifugation and reused directly in the second run following washed with suitable solvent successively. To our delight, Pd@PCC-**T** could be cyclic utilization for six successive cycles not only in Sonogashira couplings but also contained tandem reaction without obvious loss of its catalytic activity (Figure 3b, Figure S15). ICP-MS determined that the palladium loading is estimated to be ~19 wt% after six runs for tandem reaction. However, the loss of metal content may be due to the mass gain during the subsequent cycles ascribed to the adsorption of substances from the catalyst reactions [19]. The composition of the reaction mixture after the removal of the Pd@PCC-**T** was further analyzed by ICP-MS, only traces of Pd species (less than 300 ppb) was observed in the solution, which confirmed it may not be due to desorption or leaching of the Pd@PCC-**T** [20]. The grain size still kept their spherical morphology with ~2.0 nm besides Pd 3*d* and N 1*s* XPS spectrum of the recovered Pd@PCC-**T** basically remain unchanged after six runs (Figure S16-S17). The slight agglomeration of Pd@PCC-**T** after recyclability test was due to the reactant which adhered to the surface of catalyst during the catalytic process. These results clearly indicate the excellent stability and reusability of the newly heterogeneous Pd@PCC-**T**.


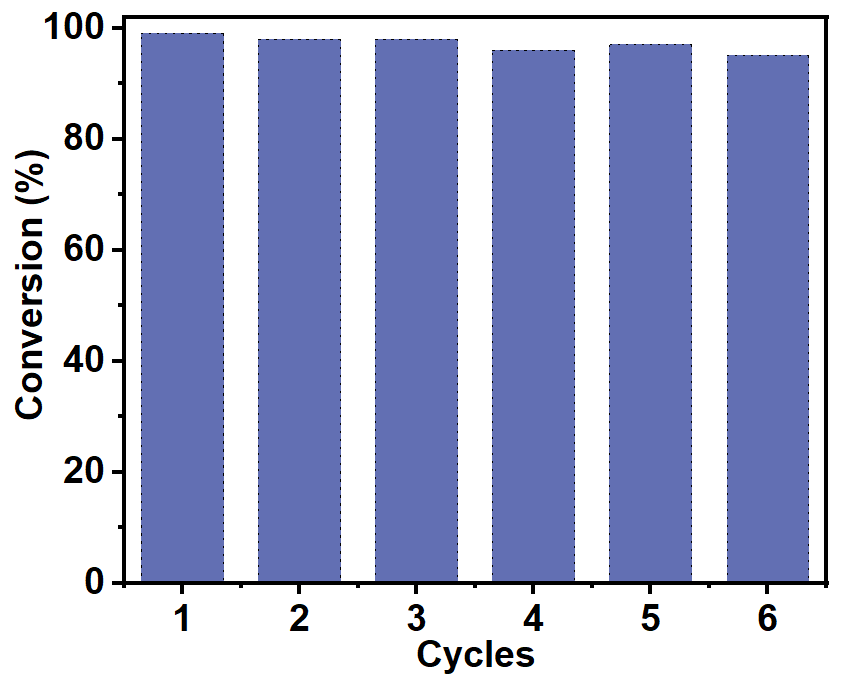


**Figure S15**. Recyclability of the Pd@PCC-**T** catalyst in Sonogashira coupling reaction.


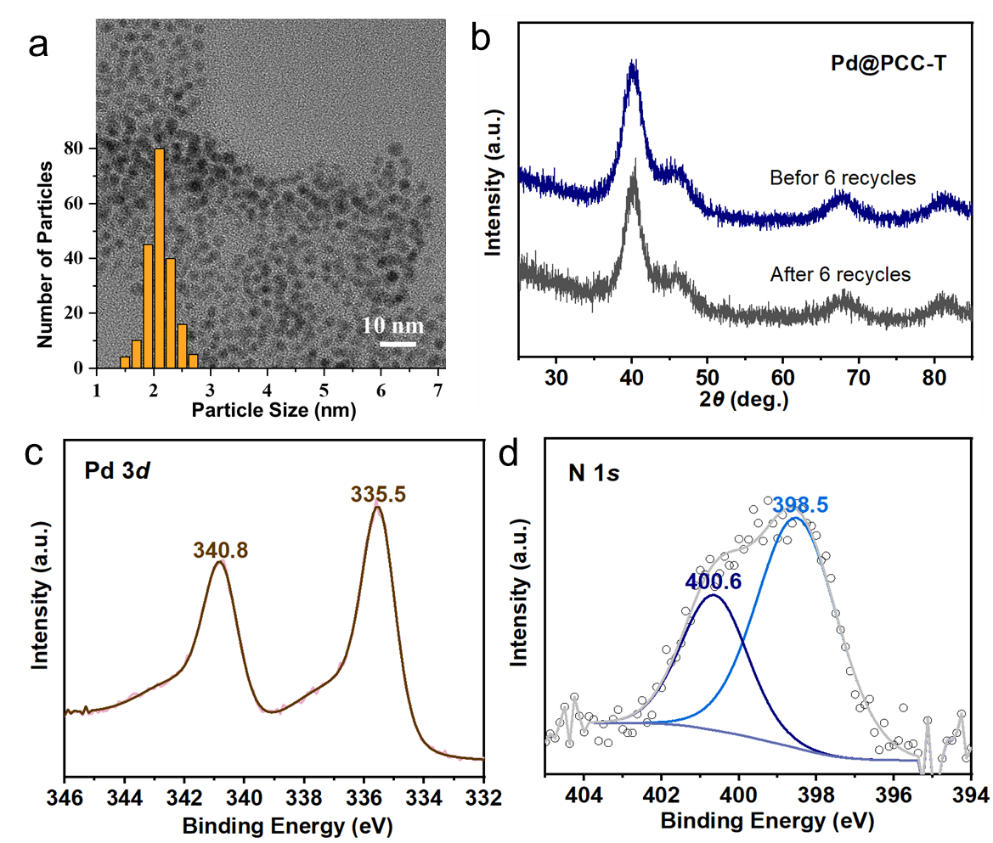


**Figure S16**. a) TEM, particle size distribution diagram, b) PXRD, c) XPS Pd 3*d* and d) N1*s* of Pd@PCC-**T** after Sonogashira coupling.


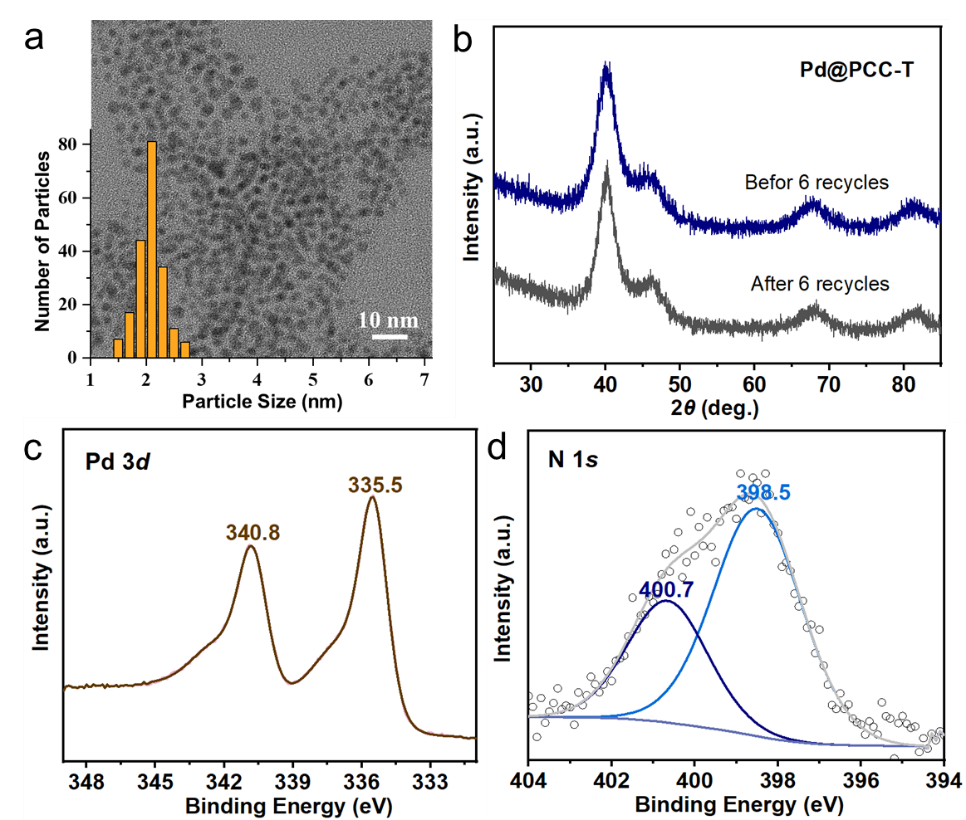


**Figure S17**. a) TEM, particle size distribution diagram, b) PXRD, c) XPS Pd 3*d* and d) N1*s* of Pd@PCC-**T** after tandem reaction.

**Note:** To determine the catalytic activity of the catalyst, a hot filtration test was further carried out with reaction between 2-iodophenol and phenylacetylene under standard conditions. After separation of the catalyst, the reaction was continued for another hour, no increase in the yield of the product was detected and the Pd content in the reaction solution is very limited, excluding the leaching of homogeneous metal species. Notably, without MNPs, PIC-**T** displays no catalytic activity, even the Pd@PCC-**I** catalyst showed obviously lower catalytic activity than that of Pd@PCC-**T**. These observations indicate that the dispersed Pd@PCC-**T** is the real catalyst in the process.

**Table S5.** The coordinates of the final optimized molecules for PIC-**T**.

| Center Number | Atomic  Number | Atomic  Type | Coordinates (Angstroms) Number | | |
| --- | --- | --- | --- | --- | --- |
|  |  |  | X | Y | Z |
| 1 | 6 | 0 | -0.134348 | -2.59733 | -6.866985 |
| 2 | 6 | 0 | 1.845046 | -4.602052 | -6.858809 |
| 3 | 6 | 0 | 5.547411 | -3.795231 | 7.190769 |
| 4 | 7 | 0 | 3.533069 | -5.887952 | -5.525944 |
| 5 | 7 | 0 | 7.500185 | -5.100472 | 6.244856 |
| 6 | 7 | 0 | 4.916423 | -6.584662 | -3.983349 |
| 7 | 7 | 0 | 8.320264 | -5.977579 | 4.420374 |
| 8 | 6 | 0 | 6.049939 | -7.197789 | -3.332623 |
| 9 | 6 | 0 | 8.524301 | -6.855272 | 3.290851 |
| 10 | 6 | 0 | 8.2291 | -8.431298 | -2.076767 |
| 11 | 6 | 0 | 9.090643 | -8.543097 | 1.118156 |
| 12 | 6 | 0 | 11.663494 | -9.030567 | -2.785055 |
| 13 | 6 | 0 | 11.839325 | -10.730405 | -0.198425 |
| 14 | 8 | 0 | 12.948172 | -9.221352 | -2.488287 |
| 15 | 8 | 0 | 11.188581 | -9.060501 | -3.903921 |
| 16 | 8 | 0 | 11.351058 | -11.539473 | -1.161313 |
| 17 | 8 | 0 | 12.69841 | -11.04046 | 0.59078 |
| 18 | 6 | 0 | 8.403422 | -7.68973 | -3.257662 |
| 19 | 1 | 0 | 9.386654 | -7.644873 | -3.714722 |
| 20 | 6 | 0 | 7.326316 | -7.062749 | -3.880319 |
| 21 | 1 | 0 | 7.488553 | -6.473839 | -4.778683 |
| 22 | 6 | 0 | 6.936668 | -8.529489 | -1.537693 |
| 23 | 1 | 0 | 6.769065 | -9.11348 | -0.637914 |
| 24 | 6 | 0 | 5.844734 | -7.929393 | -2.16125 |
| 25 | 1 | 0 | 4.844579 | -8.066495 | -1.760864 |
| 26 | 6 | 0 | 3.010093 | -5.504869 | -4.306564 |
| 27 | 1 | 0 | 2.087219 | -4.95359 | -4.235125 |
| 28 | 6 | 0 | 8.656394 | -4.404959 | 5.940069 |
| 29 | 1 | 0 | 9.031425 | -3.612109 | 6.568512 |
| 30 | 6 | 0 | 8.792221 | -6.30145 | 2.037561 |
| 31 | 1 | 0 | 8.786116 | -5.224157 | 1.899356 |
| 32 | 6 | 0 | 9.071827 | -7.145528 | 0.964551 |
| 33 | 1 | 0 | 9.288286 | -6.699185 | -0.00028 |
| 34 | 6 | 0 | 8.773449 | -9.065117 | 2.384515 |
| 35 | 1 | 0 | 8.785834 | -10.140342 | 2.537802 |
| 36 | 6 | 0 | 8.502932 | -8.238037 | 3.472827 |
| 37 | 1 | 0 | 8.32811 | -8.668517 | 4.454563 |
| 38 | 6 | 0 | 2.203916 | -3.247616 | -6.929901 |
| 39 | 1 | 0 | 3.251404 | -2.965147 | -7.00232 |
| 40 | 6 | 0 | 1.226659 | -2.256578 | -6.932618 |
| 41 | 1 | 0 | 1.506166 | -1.212054 | -7.004227 |
| 42 | 6 | 0 | -0.493329 | -3.95159 | -6.803095 |
| 43 | 1 | 0 | -1.54267 | -4.219859 | -6.7715 |
| 44 | 6 | 0 | 0.486764 | -4.942064 | -6.797631 |
| 45 | 1 | 0 | 0.189434 | -5.987373 | -6.766164 |
| 46 | 6 | 0 | 4.867042 | -1.468039 | 7.069291 |
| 47 | 1 | 0 | 5.116848 | -0.413886 | 7.095365 |
| 48 | 6 | 0 | 5.865292 | -2.429439 | 7.194952 |
| 49 | 1 | 0 | 6.894412 | -2.105692 | 7.330091 |
| 50 | 6 | 0 | 3.202706 | -3.216055 | 6.940537 |
| 51 | 1 | 0 | 2.164514 | -3.517215 | 6.863803 |
| 52 | 6 | 0 | 4.206494 | -4.175671 | 7.053335 |
| 53 | 1 | 0 | 3.935389 | -5.228633 | 7.070245 |
| 54 | 6 | 0 | 2.898884 | -5.686182 | -6.868933 |
| 55 | 1 | 0 | 2.466445 | -6.644892 | -7.167806 |
| 56 | 1 | 0 | 3.710304 | -5.445382 | -7.560746 |
| 57 | 6 | 0 | 6.615641 | -4.848266 | 7.417818 |
| 58 | 1 | 0 | 7.28265 | -4.565779 | 8.236652 |
| 59 | 1 | 0 | 6.166976 | -5.8083 | 7.682117 |
| 60 | 6 | 0 | 9.383043 | -9.206974 | -1.488473 |
| 61 | 1 | 0 | 9.376367 | -10.190574 | -1.972865 |
| 62 | 6 | 0 | 10.846998 | -8.697145 | -1.549637 |
| 63 | 1 | 0 | 10.901538 | -7.608225 | -1.421143 |
| 64 | 6 | 0 | 9.565269 | -9.499346 | 0.047624 |
| 65 | 1 | 0 | 9.247576 | -10.50865 | 0.320145 |
| 66 | 6 | 0 | 11.132826 | -9.384693 | -0.181656 |
| 67 | 1 | 0 | 11.654761 | -8.767693 | 0.549482 |
| 68 | 6 | 0 | 13.834904 | -9.505121 | -3.601009 |
| 69 | 1 | 0 | 14.821234 | -9.626587 | -3.156704 |
| 70 | 1 | 0 | 13.517687 | -10.419506 | -4.10596 |
| 71 | 1 | 0 | 13.824045 | -8.673183 | -4.307925 |
| 72 | 6 | 0 | 11.967777 | -12.849159 | -1.249374 |
| 73 | 1 | 0 | 11.461629 | -13.353062 | -2.071565 |
| 74 | 1 | 0 | 13.035124 | -12.744998 | -1.453185 |
| 75 | 1 | 0 | 11.829858 | -13.393094 | -0.312871 |
| 76 | 6 | 0 | -1.181523 | -1.539256 | -6.887959 |
| 77 | 6 | 0 | -3.375829 | -0.942465 | -6.908712 |
| 78 | 6 | 0 | -1.759984 | 0.655962 | -6.979728 |
| 79 | 6 | 0 | 3.520707 | -1.850215 | 6.952714 |
| 80 | 6 | 0 | 2.446251 | -0.822944 | 6.894498 |
| 81 | 6 | 0 | 1.807637 | 1.35904 | 6.81384 |
| 82 | 6 | 0 | 0.238712 | -0.282326 | 6.915429 |
| 83 | 6 | 0 | -4.813623 | -1.320368 | -6.901593 |
| 84 | 6 | 0 | -5.196328 | -2.67224 | -6.907989 |
| 85 | 6 | 0 | -5.809825 | -0.333896 | -6.918555 |
| 86 | 6 | 0 | -6.541323 | -3.024685 | -6.94658 |
| 87 | 1 | 0 | -4.430205 | -3.438573 | -6.910109 |
| 88 | 6 | 0 | -7.156627 | -0.690951 | -6.949416 |
| 89 | 1 | 0 | -5.521177 | 0.710539 | -6.924966 |
| 90 | 6 | 0 | -7.537947 | -2.038046 | -6.970175 |
| 91 | 1 | 0 | -6.810855 | -4.077358 | -6.984568 |
| 92 | 1 | 0 | -7.912777 | 0.089267 | -6.990034 |
| 93 | 6 | 0 | -1.368746 | 2.089777 | -7.05905 |
| 94 | 6 | 0 | -0.020391 | 2.468044 | -6.949943 |
| 95 | 6 | 0 | -2.337996 | 3.080961 | -7.268789 |
| 96 | 6 | 0 | 0.346307 | 3.806763 | -7.040938 |
| 97 | 1 | 0 | 0.732434 | 1.70159 | -6.807937 |
| 98 | 6 | 0 | -1.966775 | 4.420846 | -7.369678 |
| 99 | 1 | 0 | -3.377107 | 2.791934 | -7.37349 |
| 100 | 6 | 0 | -0.622726 | 4.79846 | -7.251785 |
| 101 | 1 | 0 | 1.396775 | 4.078405 | -6.968293 |
| 102 | 1 | 0 | -2.728145 | 5.171465 | -7.568313 |
| 103 | 6 | 0 | -1.188179 | -0.699183 | 7.004959 |
| 104 | 6 | 0 | -1.519639 | -2.028185 | 7.305116 |
| 105 | 6 | 0 | -2.220374 | 0.237505 | 6.832787 |
| 106 | 6 | 0 | -2.853327 | -2.409037 | 7.442994 |
| 107 | 1 | 0 | -0.725581 | -2.749627 | 7.457828 |
| 108 | 6 | 0 | -3.551664 | -0.147992 | 6.958325 |
| 109 | 1 | 0 | -1.965931 | 1.269011 | 6.618362 |
| 110 | 6 | 0 | -3.883431 | -1.474742 | 7.269708 |
| 111 | 1 | 0 | -3.089095 | -3.434333 | 7.718619 |
| 112 | 1 | 0 | -4.336151 | 0.595478 | 6.838725 |
| 113 | 6 | 0 | 2.163115 | 2.803084 | 6.801151 |
| 114 | 6 | 0 | 1.165934 | 3.785405 | 6.922588 |
| 115 | 6 | 0 | 3.50359 | 3.205908 | 6.723312 |
| 116 | 6 | 0 | 1.506002 | 5.132387 | 6.996446 |
| 117 | 1 | 0 | 0.129121 | 3.47856 | 6.995484 |
| 118 | 6 | 0 | 3.839548 | 4.557056 | 6.786085 |
| 119 | 1 | 0 | 4.278846 | 2.453876 | 6.635705 |
| 120 | 6 | 0 | 2.848521 | 5.534517 | 6.937842 |
| 121 | 1 | 0 | 0.716944 | 5.868089 | 7.131368 |
| 122 | 1 | 0 | 4.887111 | 4.846103 | 6.752656 |
| 123 | 6 | 0 | -0.225347 | 6.253678 | -7.378851 |
| 124 | 1 | 0 | 0.690685 | 6.371138 | -7.963094 |
| 125 | 1 | 0 | -1.011958 | 6.836854 | -7.86536 |
| 126 | 6 | 0 | 3.252807 | 6.981737 | 7.151985 |
| 127 | 1 | 0 | 2.865361 | 7.359745 | 8.102421 |
| 128 | 1 | 0 | 4.340166 | 7.07749 | 7.190176 |
| 129 | 7 | 0 | 0.040144 | 6.89269 | -6.055135 |
| 130 | 6 | 0 | -0.791494 | 6.834245 | -4.952928 |
| 131 | 1 | 0 | -1.703064 | 6.258261 | -4.960064 |
| 132 | 7 | 0 | 0.968845 | 8.092774 | -4.483396 |
| 133 | 7 | 0 | 2.773195 | 7.937983 | 6.117645 |
| 134 | 6 | 0 | 1.615231 | 8.692139 | 6.186921 |
| 135 | 1 | 0 | 0.931659 | 8.617072 | 7.018534 |
| 136 | 7 | 0 | 2.720008 | 9.207164 | 4.340213 |
| 137 | 6 | 0 | 1.915434 | 8.914635 | -3.768607 |
| 138 | 6 | 0 | 3.280898 | 8.656635 | -3.88605 |
| 139 | 6 | 0 | 1.451378 | 9.956072 | -2.961719 |
| 140 | 6 | 0 | 4.183647 | 9.472272 | -3.207262 |
| 141 | 1 | 0 | 3.641928 | 7.826114 | -4.48586 |
| 142 | 6 | 0 | 2.370192 | 10.747527 | -2.276108 |
| 143 | 1 | 0 | 0.389722 | 10.174618 | -2.894887 |
| 144 | 6 | 0 | 3.755878 | 10.536341 | -2.394977 |
| 145 | 1 | 0 | 5.247042 | 9.280636 | -3.317993 |
| 146 | 1 | 0 | 1.992082 | 11.559736 | -1.664001 |
| 147 | 6 | 0 | 3.130898 | 9.879253 | 3.127857 |
| 148 | 6 | 0 | 3.564801 | 9.138189 | 2.028619 |
| 149 | 6 | 0 | 3.121253 | 11.27478 | 3.095987 |
| 150 | 6 | 0 | 4.020615 | 9.812696 | 0.894502 |
| 151 | 1 | 0 | 3.543495 | 8.052042 | 2.050971 |
| 152 | 6 | 0 | 3.573697 | 11.931345 | 1.95542 |
| 153 | 1 | 0 | 2.8148 | 11.84761 | 3.966399 |
| 154 | 6 | 0 | 4.046023 | 11.21482 | 0.841075 |
| 155 | 1 | 0 | 4.376961 | 9.231836 | 0.049576 |
| 156 | 1 | 0 | 3.636667 | 13.015108 | 1.951728 |
| 157 | 6 | 0 | 4.698855 | 11.962704 | -0.298191 |
| 158 | 1 | 0 | 5.737811 | 12.146914 | 0.001051 |
| 159 | 6 | 0 | 4.797433 | 11.443283 | -1.782928 |
| 160 | 1 | 0 | 5.784118 | 11.02564 | -1.99549 |
| 161 | 6 | 0 | 4.672261 | 12.949642 | -2.250077 |
| 162 | 1 | 0 | 3.978382 | 13.137786 | -3.069828 |
| 163 | 6 | 0 | 4.157162 | 13.320139 | -0.824207 |
| 164 | 1 | 0 | 3.060325 | 13.352449 | -0.824187 |
| 165 | 6 | 0 | 6.040984 | 13.532988 | -2.562984 |
| 166 | 6 | 0 | 4.638247 | 14.585784 | -0.1335 |
| 167 | 8 | 0 | 7.000054 | 13.394946 | -1.828135 |
| 168 | 8 | 0 | 4.827288 | 14.657628 | 1.064548 |
| 169 | 8 | 0 | 6.052353 | 14.173925 | -3.731377 |
| 170 | 8 | 0 | 4.772105 | 15.595121 | -0.993568 |
| 171 | 6 | 0 | 7.310234 | 14.789801 | -4.105658 |
| 172 | 1 | 0 | 8.091269 | 14.030952 | -4.186701 |
| 173 | 1 | 0 | 7.123084 | 15.262301 | -5.068528 |
| 174 | 1 | 0 | 7.599223 | 15.530737 | -3.357875 |
| 175 | 6 | 0 | -9.003532 | -2.407161 | -7.10193 |
| 176 | 1 | 0 | -9.164516 | -3.098741 | -7.933169 |
| 177 | 1 | 0 | -9.613545 | -1.522123 | -7.296706 |
| 178 | 6 | 0 | -5.326164 | -1.889475 | 7.462557 |
| 179 | 1 | 0 | -5.900749 | -1.112393 | 7.972555 |
| 180 | 1 | 0 | -5.394351 | -2.804631 | 8.057303 |
| 181 | 7 | 0 | -9.581549 | -3.078386 | -5.905093 |
| 182 | 6 | 0 | -9.740606 | -4.442329 | -5.739954 |
| 183 | 1 | 0 | -9.439745 | -5.150105 | -6.497066 |
| 184 | 7 | 0 | -6.035997 | -2.152695 | 6.173492 |
| 185 | 6 | 0 | -5.534921 | -2.876615 | 5.108283 |
| 186 | 1 | 0 | -4.525897 | -3.256594 | 5.106813 |
| 187 | 7 | 0 | -10.52548 | -3.388374 | -3.95955 |
| 188 | 7 | 0 | -7.619579 | -2.270431 | 4.672906 |
| 189 | 6 | 0 | -11.175429 | -3.123378 | -2.697062 |
| 190 | 6 | 0 | -12.368026 | -3.785507 | -2.398659 |
| 191 | 6 | 0 | -10.62702 | -2.198629 | -1.808608 |
| 192 | 6 | 0 | -13.019374 | -3.505178 | -1.201711 |
| 193 | 1 | 0 | -12.812571 | -4.476756 | -3.108606 |
| 194 | 6 | 0 | -11.298741 | -1.920973 | -0.616248 |
| 195 | 1 | 0 | -9.68451 | -1.707197 | -2.03401 |
| 196 | 6 | 0 | -12.504037 | -2.561819 | -0.293573 |
| 197 | 1 | 0 | -13.977691 | -3.970634 | -0.991534 |
| 198 | 1 | 0 | -10.875954 | -1.18627 | 0.061412 |
| 199 | 6 | 0 | -8.881761 | -2.080386 | 3.998522 |
| 200 | 6 | 0 | -9.477308 | -0.819536 | 3.992075 |
| 201 | 6 | 0 | -9.492295 | -3.164083 | 3.362756 |
| 202 | 6 | 0 | -10.712573 | -0.659305 | 3.368386 |
| 203 | 1 | 0 | -8.986644 | 0.030901 | 4.456639 |
| 204 | 6 | 0 | -10.720718 | -2.979361 | 2.731849 |
| 205 | 1 | 0 | -9.038784 | -4.150451 | 3.392183 |
| 206 | 6 | 0 | -11.36825 | -1.730437 | 2.737603 |
| 207 | 1 | 0 | -11.184268 | 0.318852 | 3.386246 |
| 208 | 1 | 0 | -11.189738 | -3.835472 | 2.257664 |
| 209 | 6 | 0 | -14.054554 | -3.250153 | 1.781172 |
| 210 | 1 | 0 | -13.460485 | -4.167874 | 1.879131 |
| 211 | 6 | 0 | -13.838281 | -2.360347 | 3.041593 |
| 212 | 1 | 0 | -13.43344 | -2.877353 | 3.911573 |
| 213 | 6 | 0 | -13.332028 | -2.183892 | 0.913823 |
| 214 | 1 | 0 | -14.112373 | -1.504186 | 0.551935 |
| 215 | 6 | 0 | -12.772821 | -1.512931 | 2.225442 |
| 216 | 1 | 0 | -12.977816 | -0.439899 | 2.24835 |
| 217 | 6 | 0 | 5.203504 | 16.863409 | -0.432977 |
| 218 | 1 | 0 | 6.200294 | 16.755879 | -0.001173 |
| 219 | 1 | 0 | 5.213074 | 17.558025 | -1.271174 |
| 220 | 1 | 0 | 4.501179 | 17.189355 | 0.336301 |
| 221 | 6 | 0 | -15.454004 | -3.672977 | 1.36875 |
| 222 | 6 | 0 | -15.006714 | -1.524973 | 3.537292 |
| 223 | 8 | 0 | -15.239128 | -1.333111 | 4.706759 |
| 224 | 8 | 0 | -15.835282 | -3.691872 | 0.215186 |
| 225 | 8 | 0 | -15.702357 | -0.985169 | 2.516298 |
| 226 | 8 | 0 | -16.168995 | -4.065143 | 2.422796 |
| 227 | 6 | 0 | -17.51207 | -4.543705 | 2.15077 |
| 228 | 1 | 0 | -17.924549 | -4.805982 | 3.123433 |
| 229 | 1 | 0 | -18.09972 | -3.756163 | 1.675309 |
| 230 | 1 | 0 | -17.470767 | -5.416156 | 1.495785 |
| 231 | 6 | 0 | -16.840419 | -0.169774 | 2.893148 |
| 232 | 1 | 0 | -16.509333 | 0.686658 | 3.48409 |
| 233 | 1 | 0 | -17.28845 | 0.153171 | 1.954551 |
| 234 | 1 | 0 | -17.545898 | -0.762016 | 3.478846 |
| 235 | 6 | 0 | -10.068953 | -2.462676 | -4.818908 |
| 236 | 6 | 0 | 1.091498 | 7.665614 | -5.752138 |
| 237 | 6 | 0 | 4.68128 | -6.546693 | -5.305742 |
| 238 | 6 | 0 | 3.426297 | 8.272145 | 4.995603 |
| 239 | 1 | 0 | 4.388831 | 7.893913 | 4.688135 |
| 240 | 1 | 0 | 1.892252 | 7.933618 | -6.42458 |
| 241 | 6 | 0 | -7.299141 | -1.805143 | 5.892607 |
| 242 | 1 | 0 | -7.968008 | -1.269451 | 6.54937 |
| 243 | 1 | 0 | -10.130716 | -1.395691 | -4.67258 |
| 244 | 6 | 0 | 7.317787 | -6.044504 | 5.311702 |
| 245 | 1 | 0 | 5.302778 | -7.002382 | -6.061948 |
| 246 | 1 | 0 | 6.49869 | -6.745772 | 5.274268 |
| 247 | 7 | 0 | -2.464866 | -1.927509 | -6.865506 |
| 248 | 7 | 0 | -3.068236 | 0.363076 | -6.965676 |
| 249 | 7 | 0 | -0.780076 | -0.259863 | -6.932751 |
| 250 | 7 | 0 | 1.172385 | -1.244652 | 6.937183 |
| 251 | 7 | 0 | 2.809853 | 0.466447 | 6.8275 |
| 252 | 7 | 0 | 0.505926 | 1.03 | 6.842061 |
| 253 | 6 | 0 | -6.520563 | -2.949736 | 4.170228 |
| 254 | 1 | 0 | -6.535014 | -3.398486 | 3.190173 |
| 255 | 6 | 0 | 1.578932 | 9.483104 | 5.077284 |
| 256 | 1 | 0 | 0.8535 | 10.210055 | 4.747581 |
| 257 | 6 | 0 | 9.171144 | -4.950952 | 4.802508 |
| 258 | 1 | 0 | 10.079315 | -4.737601 | 4.260824 |
| 259 | 6 | 0 | -10.327892 | -4.638619 | -4.525574 |
| 260 | 1 | 0 | -10.61673 | -5.544603 | -4.016548 |
| 261 | 6 | 0 | -0.213776 | 7.581158 | -3.970943 |
| 262 | 1 | 0 | -0.52077 | 7.776446 | -2.956195 |
| 263 | 6 | 0 | 3.8739 | -5.934168 | -3.343935 |
| 264 | 1 | 0 | 3.857647 | -5.813806 | -2.273266 |

# 7. Selected NMR and MS spectra for new compounds


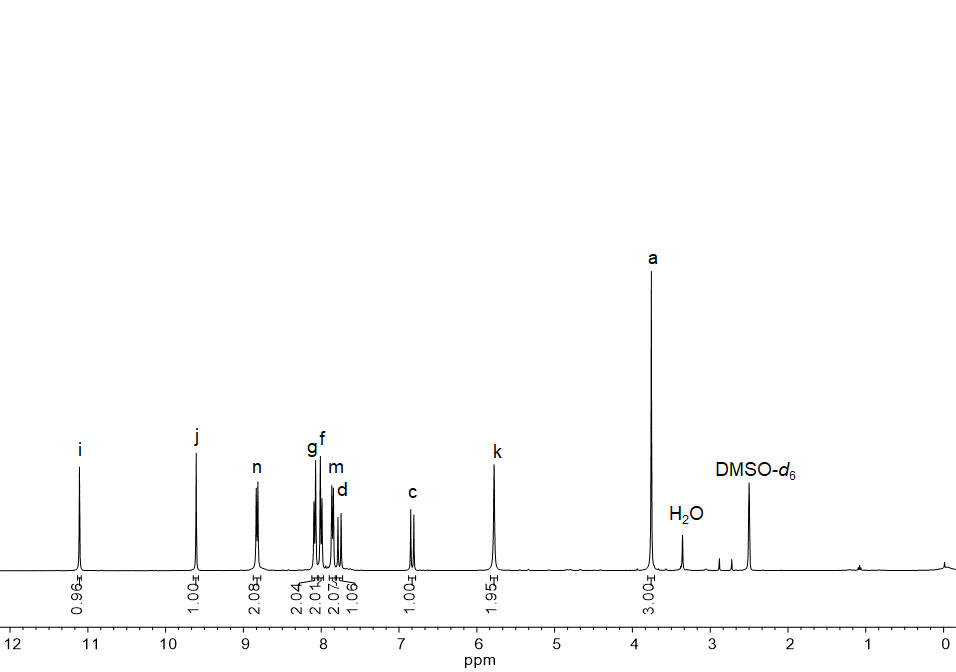


**Figure S18**. ^1^H NMR spectrum (400 MHz, 298 K, DMSO-*d*_6_) of H_3_-**1**(BF_4_)_3_.

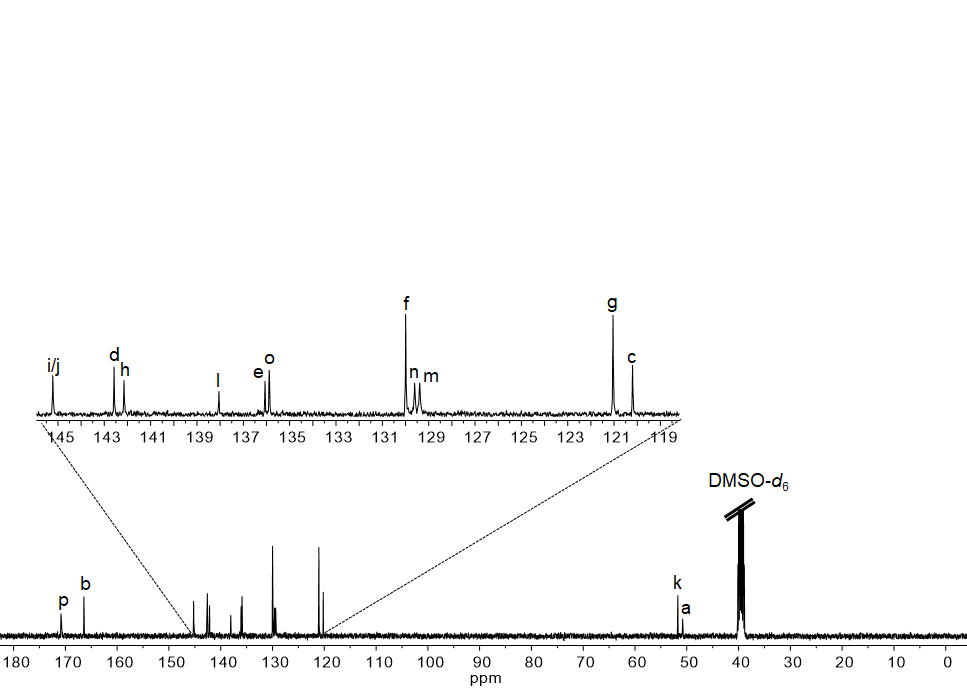


**Figure S19**. ^13^C{^1^H} NMR spectrum (100 MHz, 298 K, DMSO-*d*_6_) of H_3_-**1**(BF_4_)_3_.


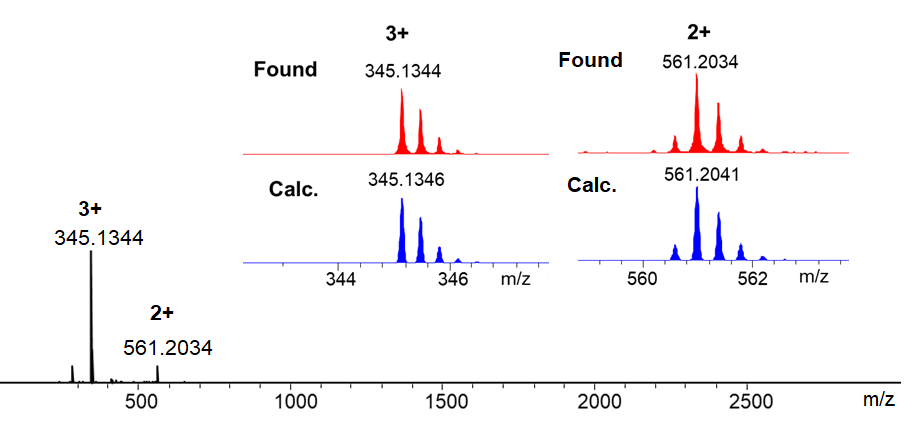


**Figure S20**. HR-ESI mass spectrum (positive ions) of H_3_-**1**(BF_4_)_3_.


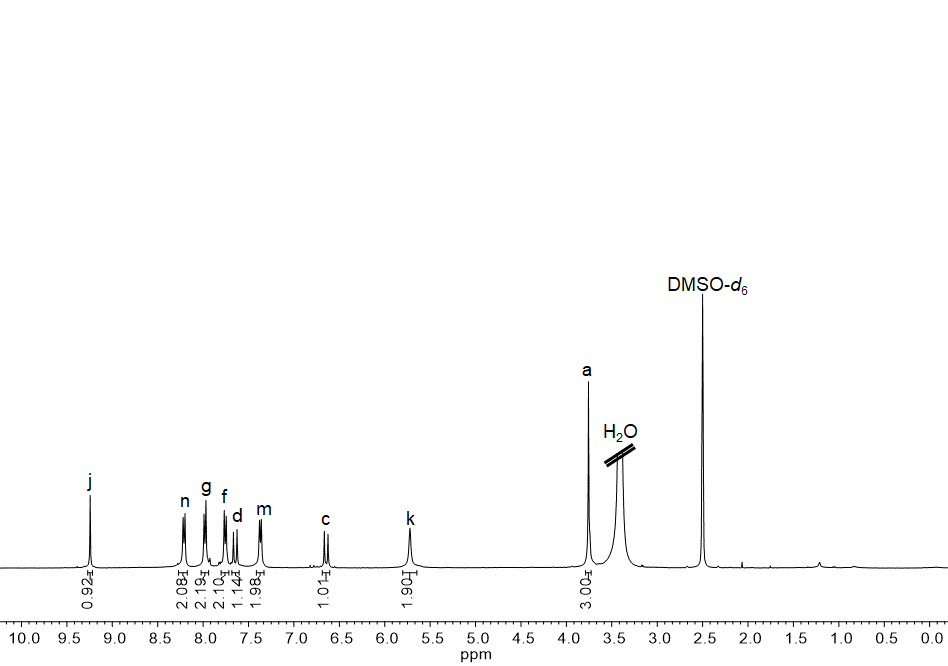


**Figure S21**. ^1^H NMR spectrum (400 MHz, 298 K, DMSO-*d*_6_) of [Ag_3_(**1**)_2_](BF_4_)_3_.


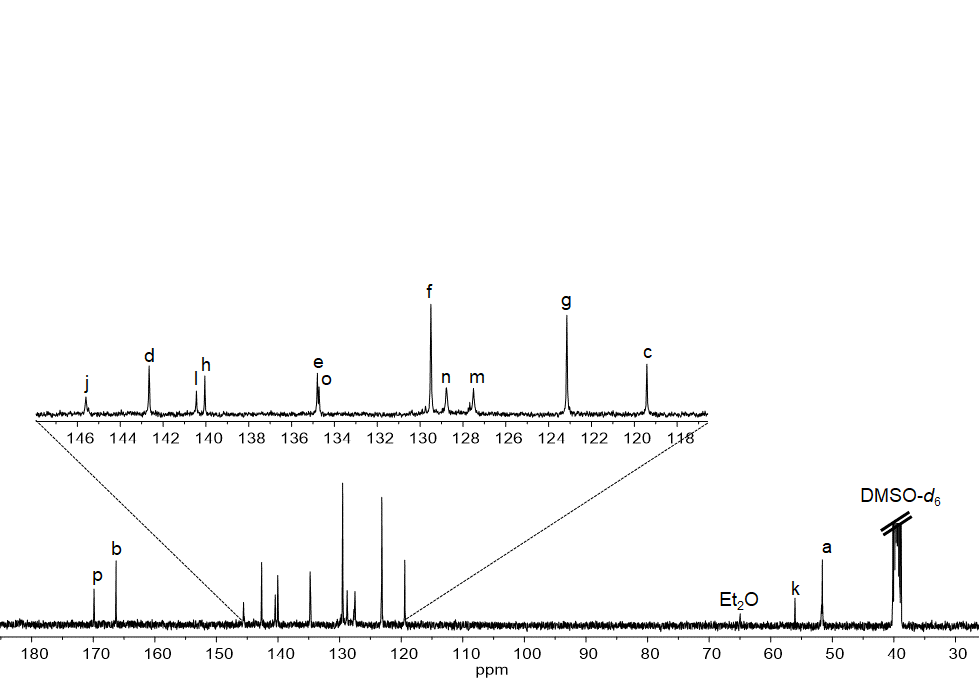


**Figure S22**. ^13^C{^1^H} NMR spectrum (100 MHz, 298 K, DMSO-*d*_6_) of [Ag_3_(**1**)_2_](BF_4_)_3_.


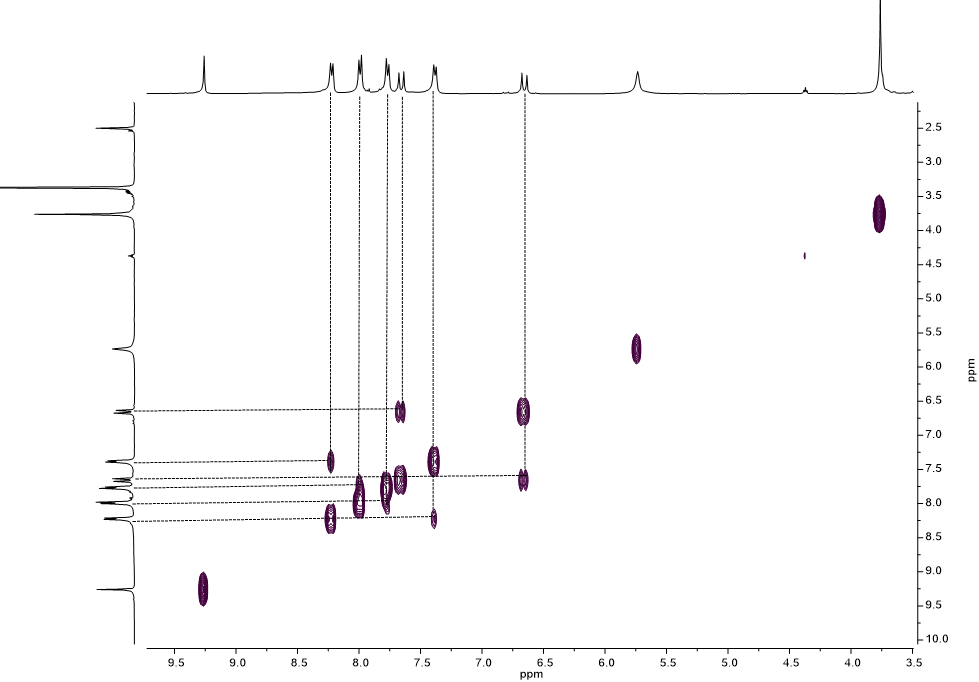


**Figure S23**. ^1^H-^1^H COSY spectrum (400 MHz, 298 K, DMSO-*d*_6_) of [Ag_3_(**1**)_2_](BF_4_)_3_.


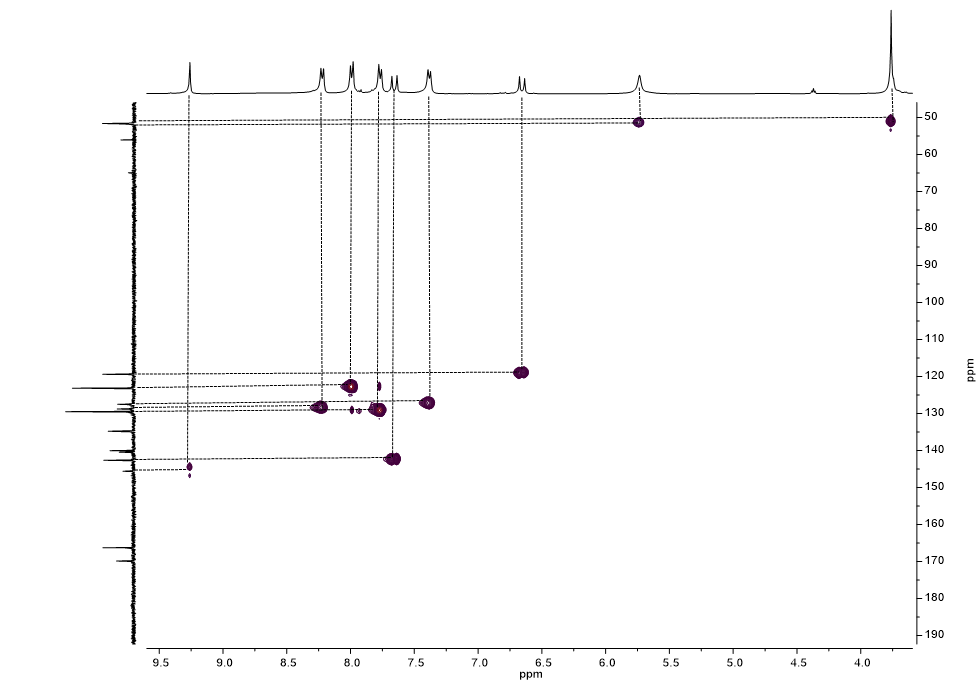


**Figure S24**. ^1^H-^13^C HSQC spectrum (400 MHz, 298 K, DMSO-*d*_6_) of [Ag_3_(**1**)_2_](BF_4_)_3_.


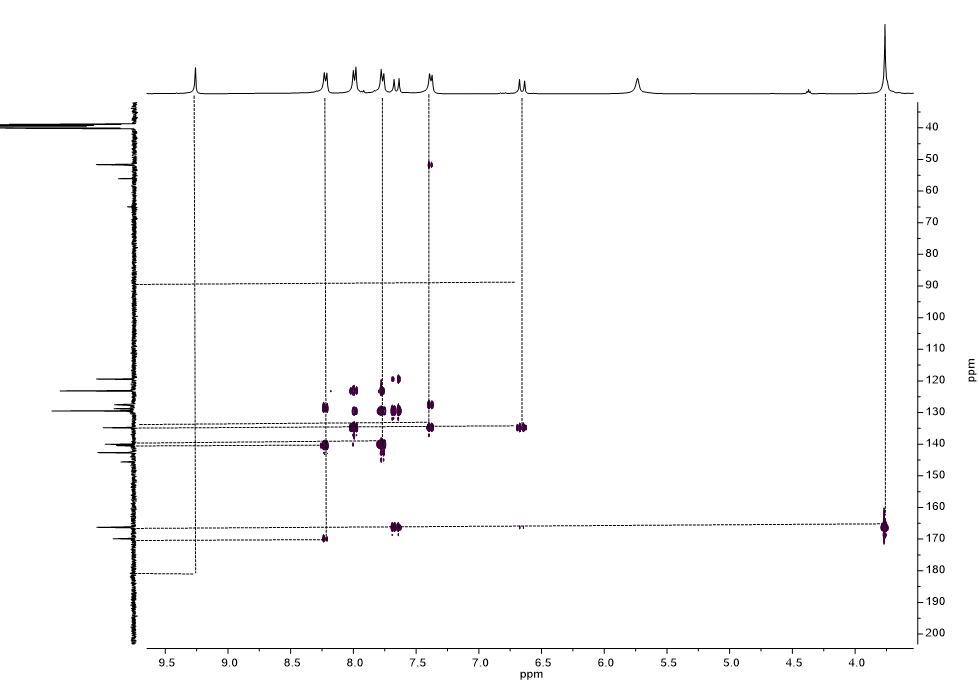


**Figure S25**. ^1^H-^13^C HMBC spectrum (400 MHz, 298 K, DMSO-*d*_6_) of [Ag_3_(**1**)_2_](BF_4_)_3_.


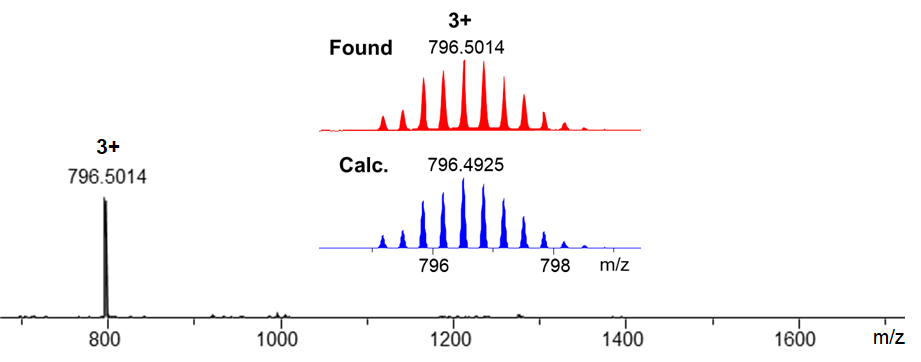


**Figure S26**. HR-ESI mass spectrum (positive ions) of [Ag_3_(**1**)_2_](BF_4_)_3_.


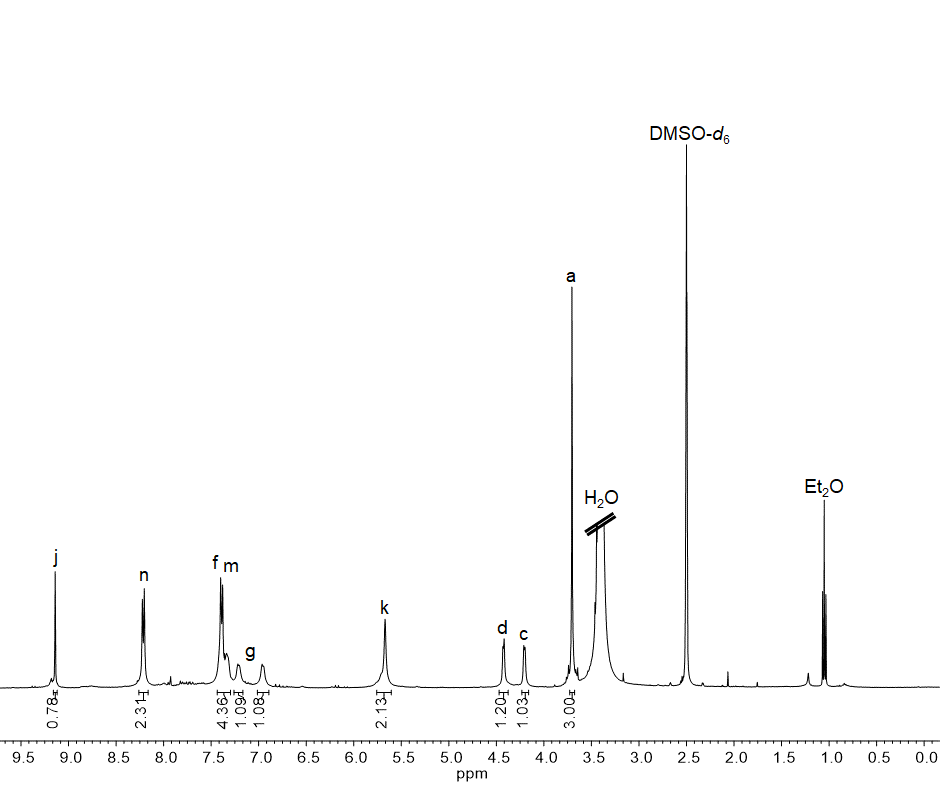


**Figure S27**. ^1^H NMR spectrum (400 MHz, 298 K, DMSO-*d*_6_) of [Ag_3_(**2**)](BF_4_)_3_.


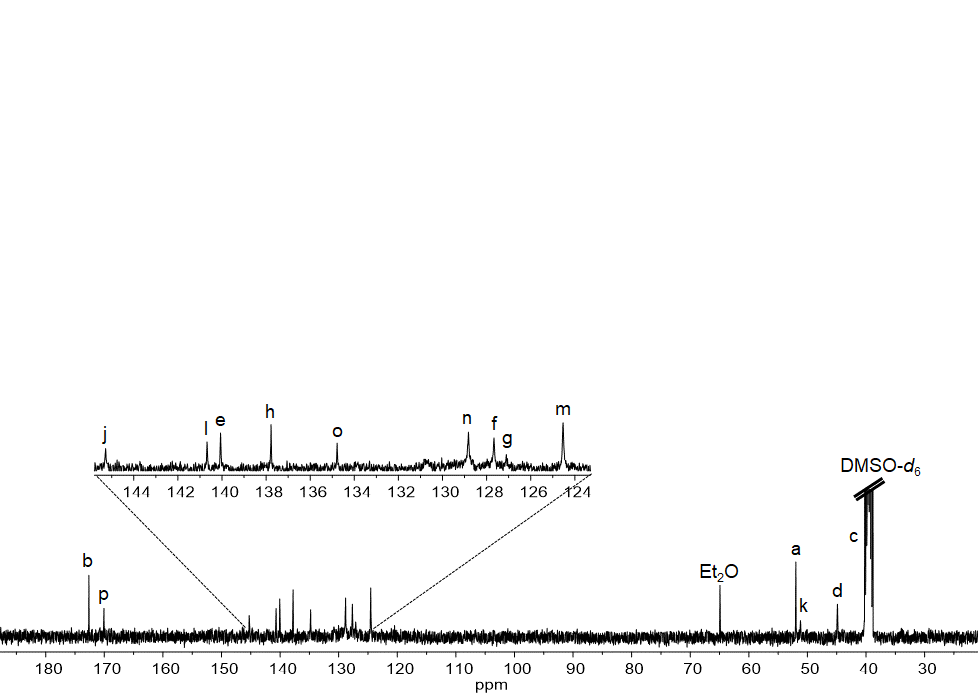


**Figure S28**. ^13^C{^1^H} NMR spectrum (100 MHz, 298 K, DMSO-*d*_6_) of [Ag_3_(**2**)](BF_4_)_3_.


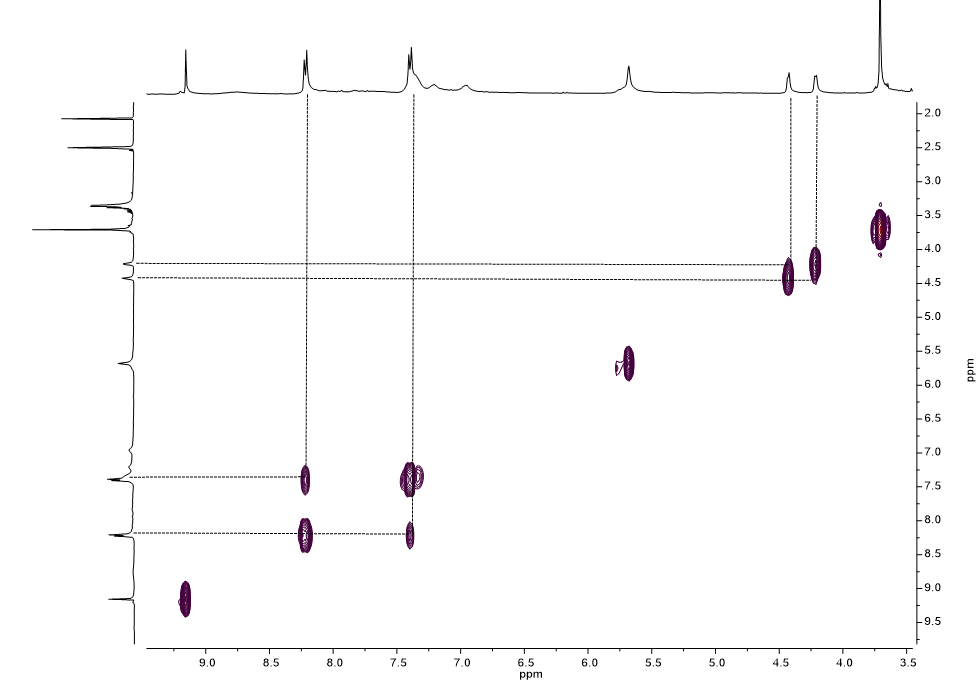


**Figure S29**. ^1^H-^1^H COSY spectrum (400 MHz, 298 K, DMSO-*d*_6_) of [Ag_3_(**2**)](BF_4_)_3_.


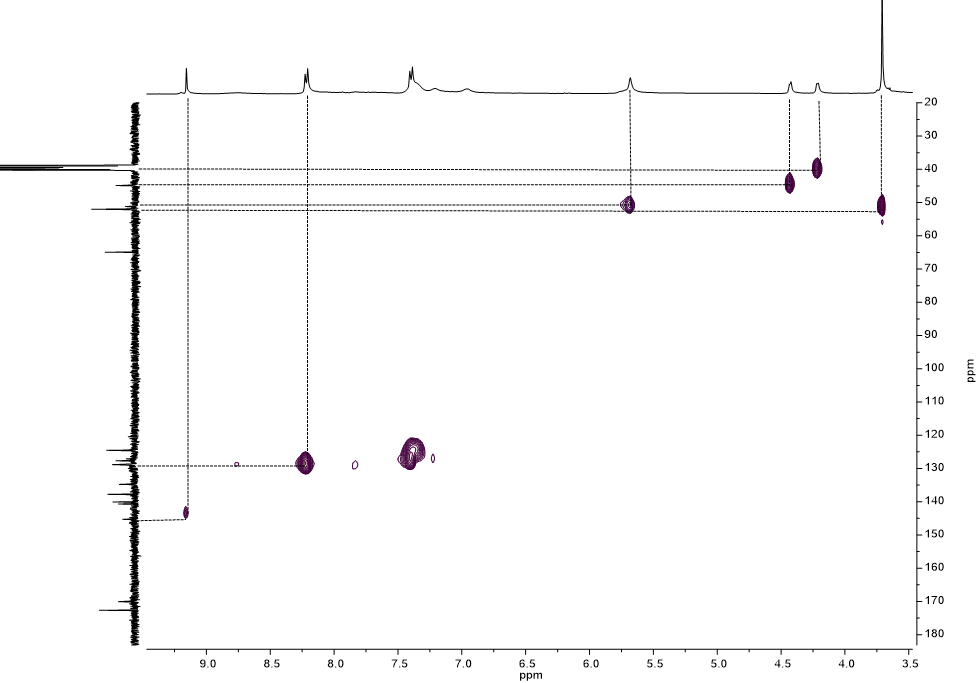


**Figure S30**. ^1^H-^13^C HSQC spectrum (400 MHz, 298 K, DMSO-*d*_6_) of [Ag_3_(**2**)](BF_4_)_3_.


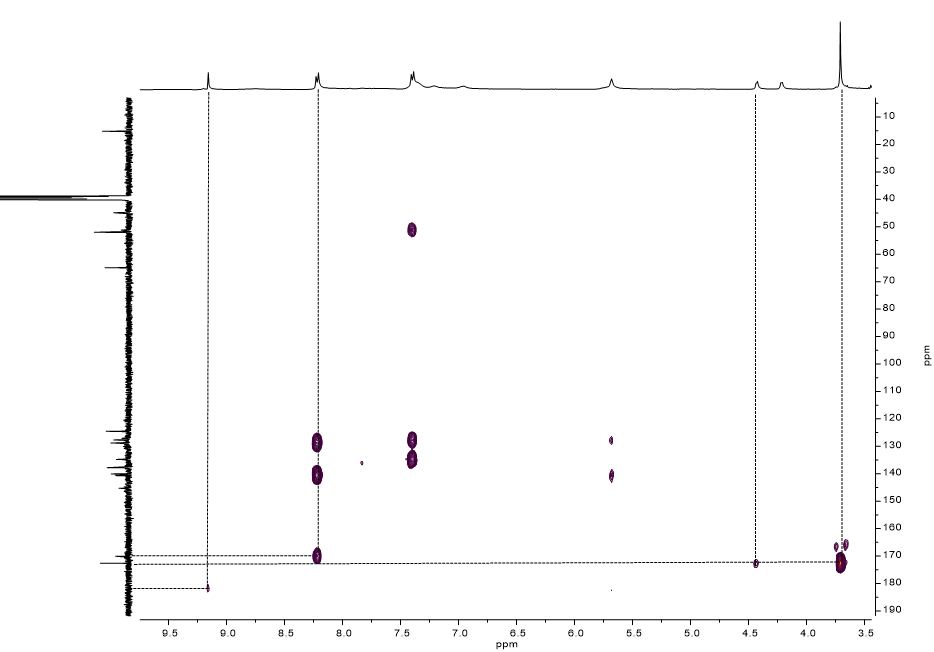


**Figure S31**. ^1^H-^13^C HMBC spectrum (400 MHz, 298 K, DMSO-*d*_6_) of [Ag_3_(**2**)](BF_4_)_3_.


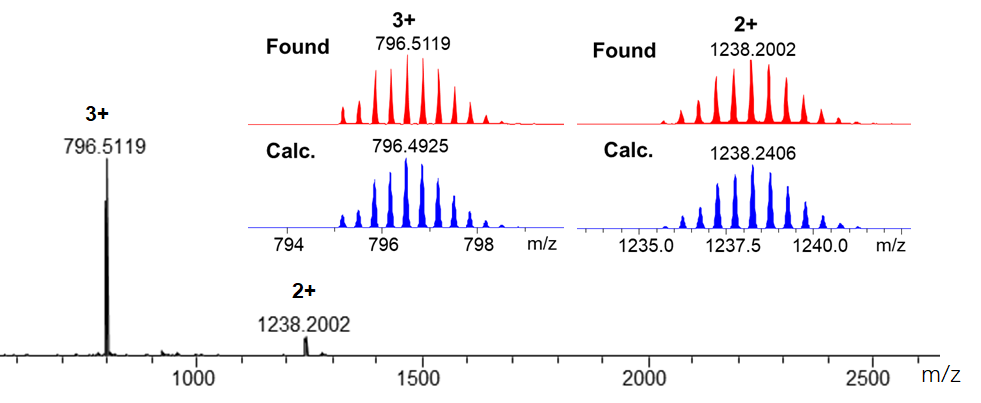


**Figure S32**. HR-ESI mass spectrum (positive ions) of [Ag_3_(**2**)](BF_4_)_3_.


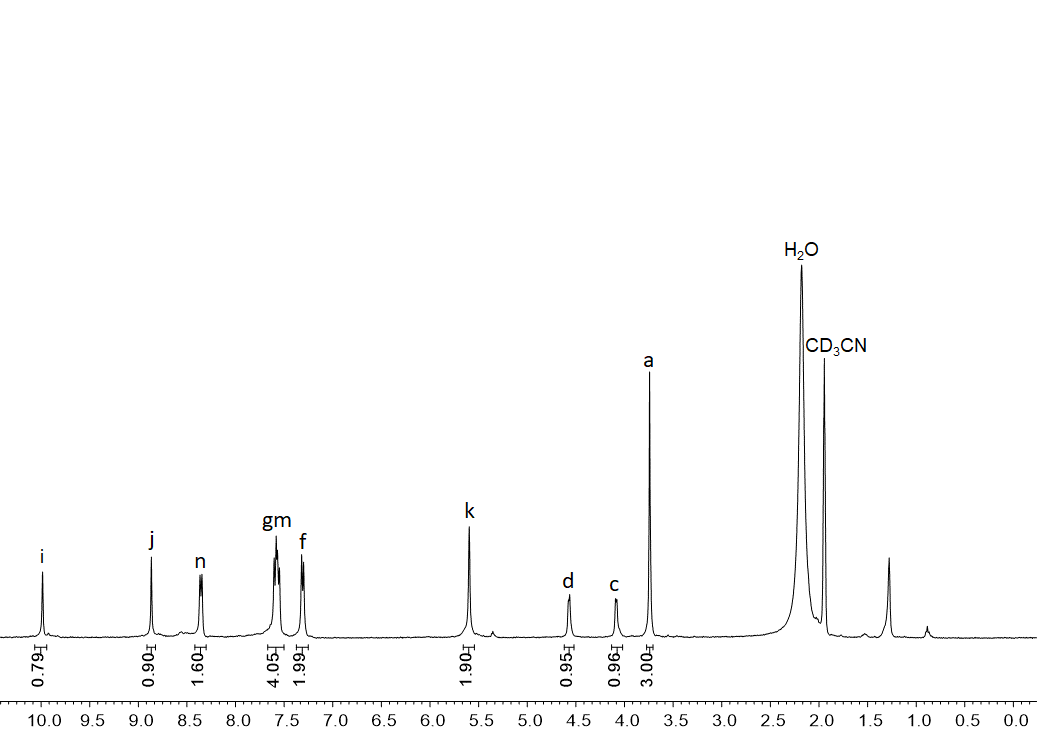


**Figure S33**.^1^H NMR spectrum (600 MHz, 298 K, CD_3_CN) of PIC-**T**.


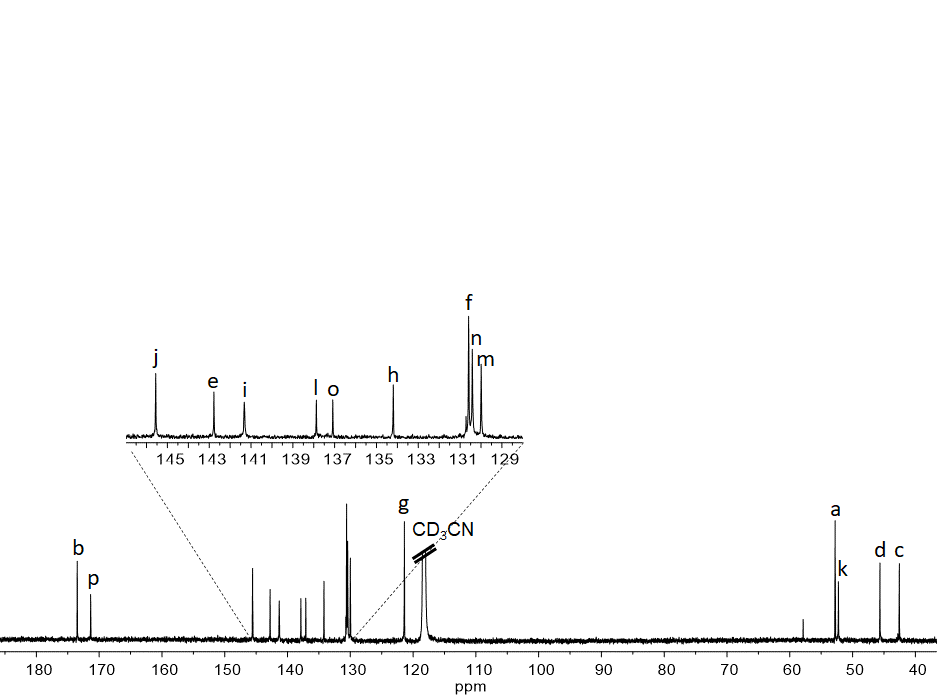


**Figure S34**. ^13^C{^1^H} spectrum (150 MHz, 298 K, CD_3_CN) of PIC-**T**.


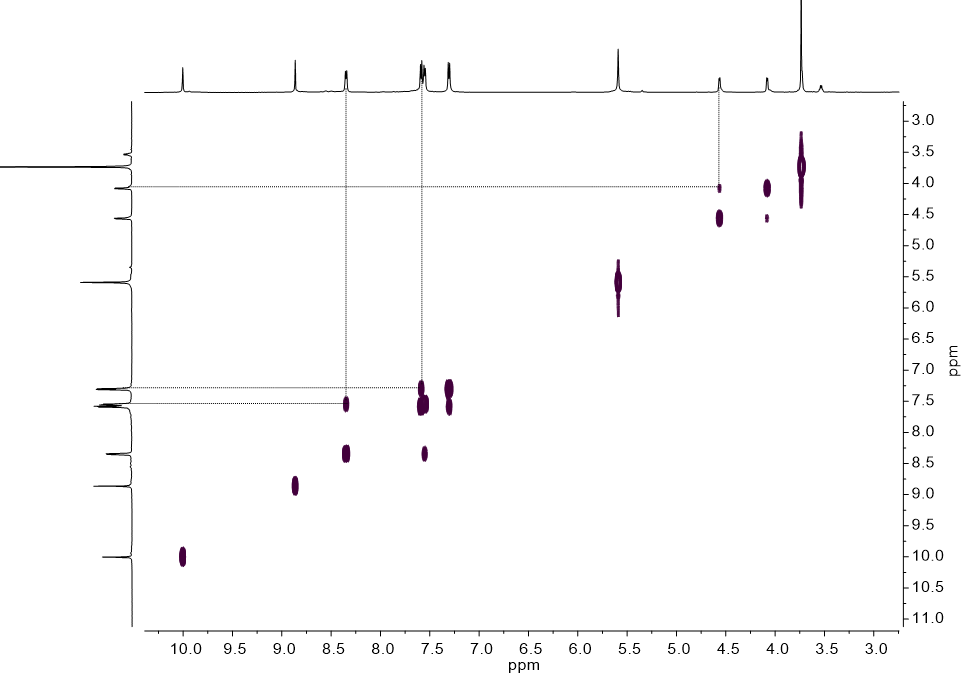


**Figure S35**. ^1^H-^1^H COSY spectrum (600 MHz, 298 K, CD_3_CN) of PIC-**T**.


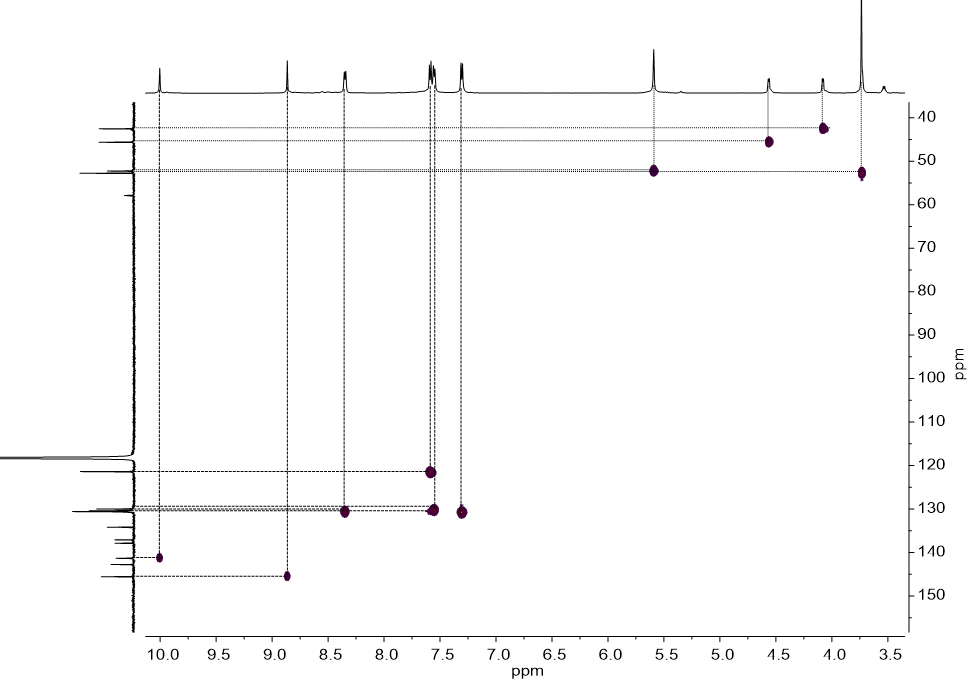


**Figure S36**. ^1^H-^13^C HSQC spectrum (600 MHz, 298 K, CD_3_CN) of PIC-**T**.


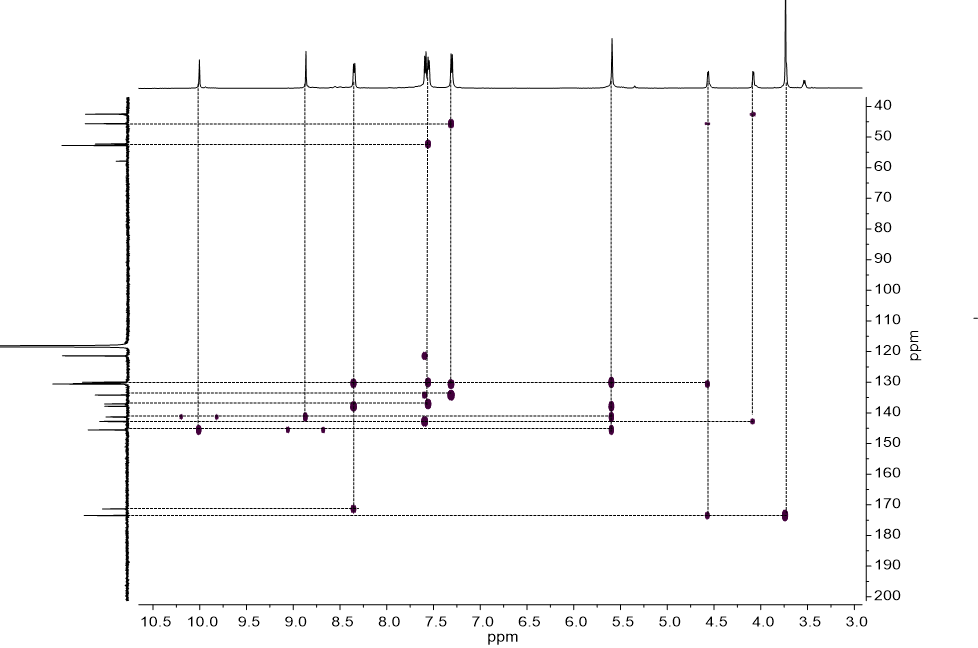


**Figure S37**. ^1^H-^13^C HMBC spectrum (600 MHz, 298 K, CD_3_CN) of PIC-**T**.


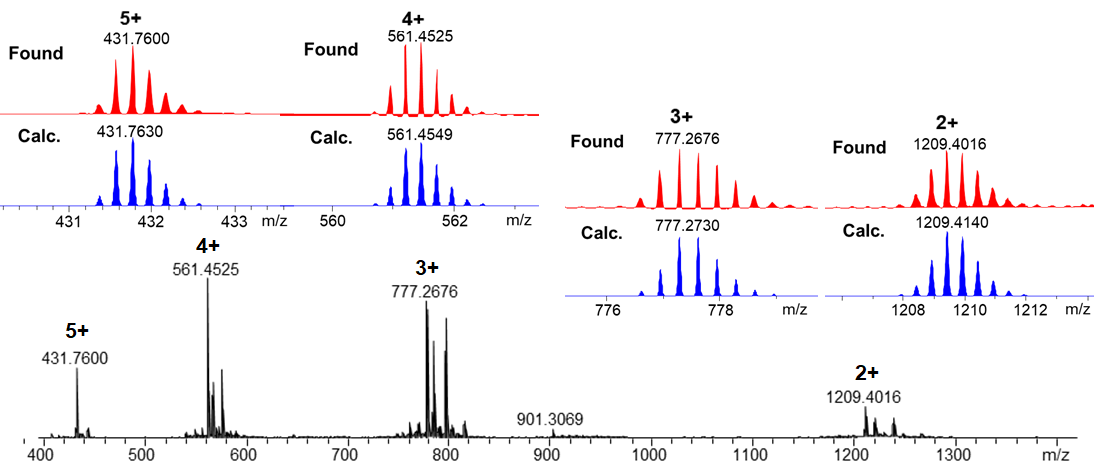


**Figure S38**. HR-ESI mass spectrum (positive ions) of PIC-**T**.


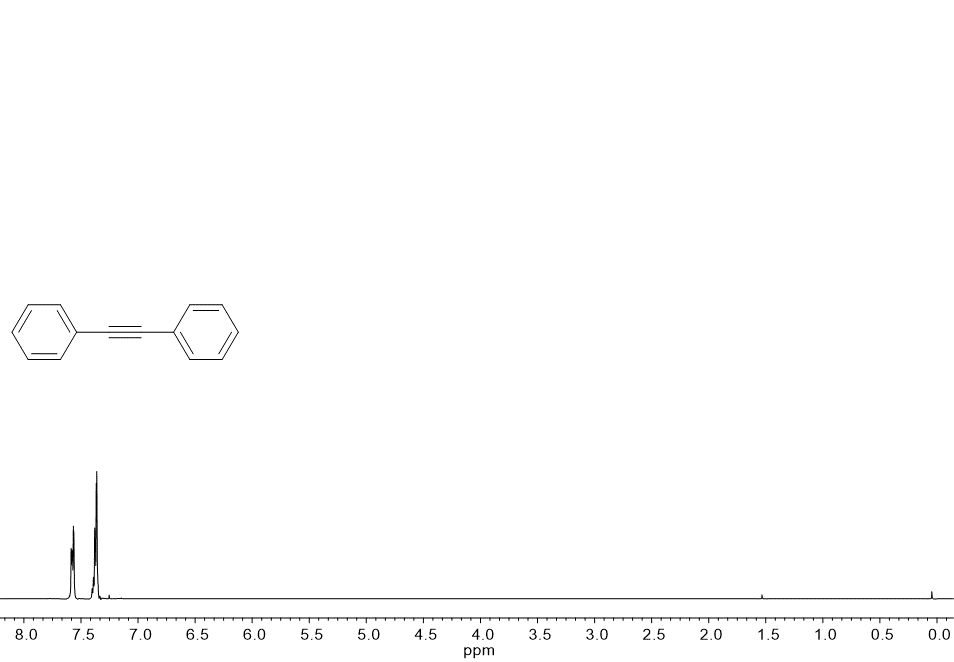


**Figure S39**. ^1^H NMR spectrum (400 MHz, 298 K, CDCl_3_) of **3a**.


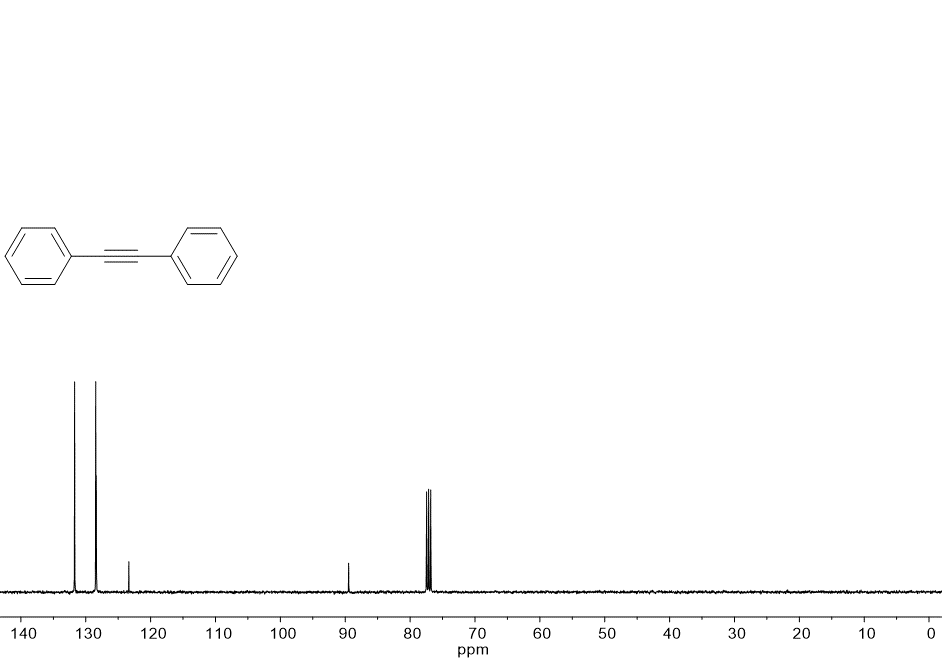


**Figure 40**. ^13^C{^1^H} NMR spectrum (100 MHz, 298 K, CDCl_3_) of **3a**.


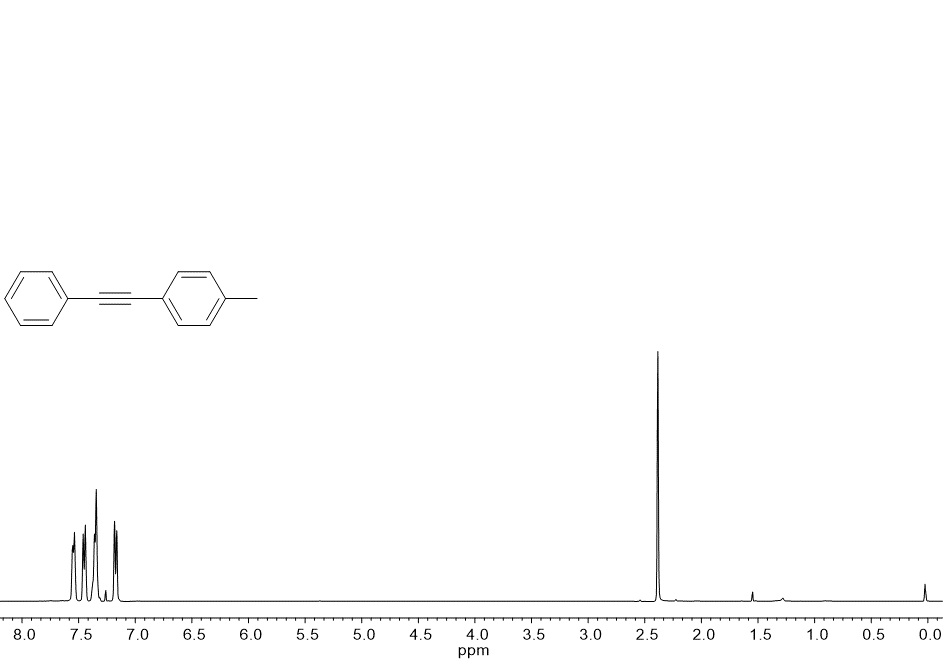


**Figure S41**. ^1^H NMR spectrum (400 MHz, 298 K, CDCl_3_) of **3b**.


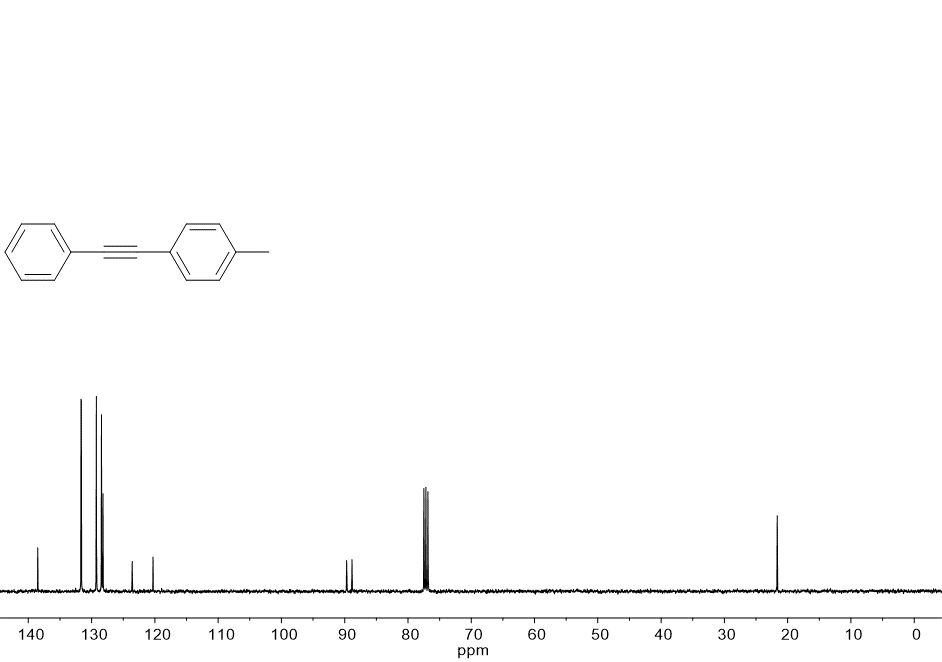


**Figure S42**. ^13^C{^1^H} NMR spectrum (100 MHz, 298 K, CDCl_3_) of **3b**.


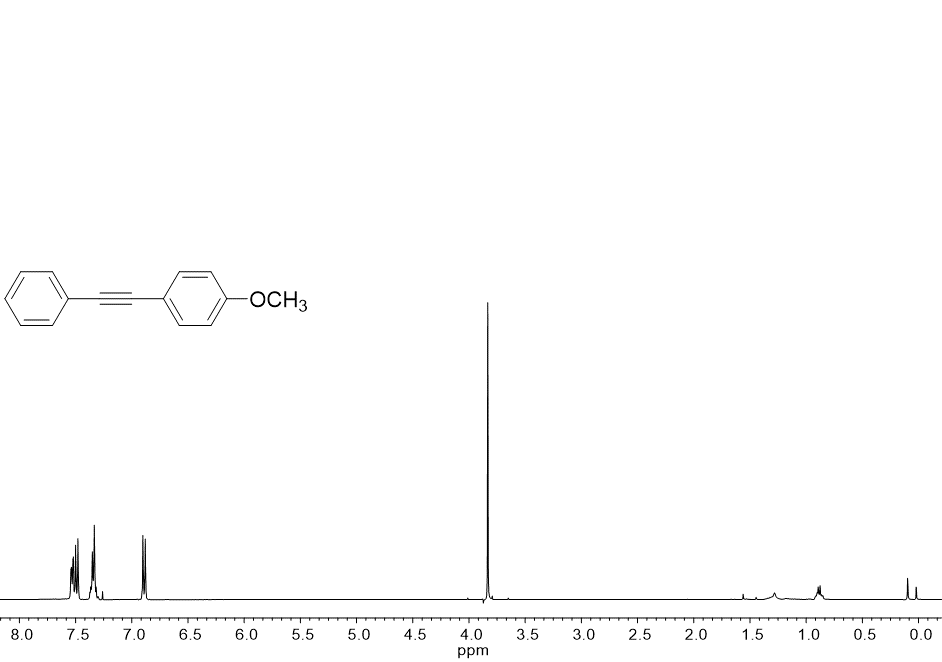


**Figure S43**. ^1^H NMR spectrum (400 MHz, 298 K, CDCl_3_) of **3c**.


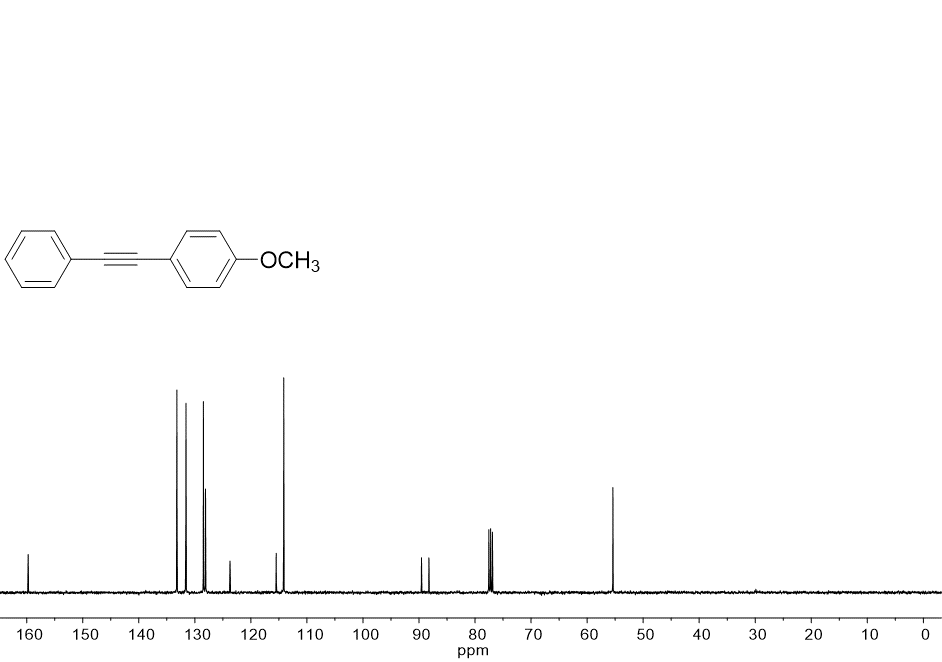


**Figure S44**. ^13^C{^1^H} NMR spectrum (100 MHz, 298 K, CDCl_3_) of **3c**.


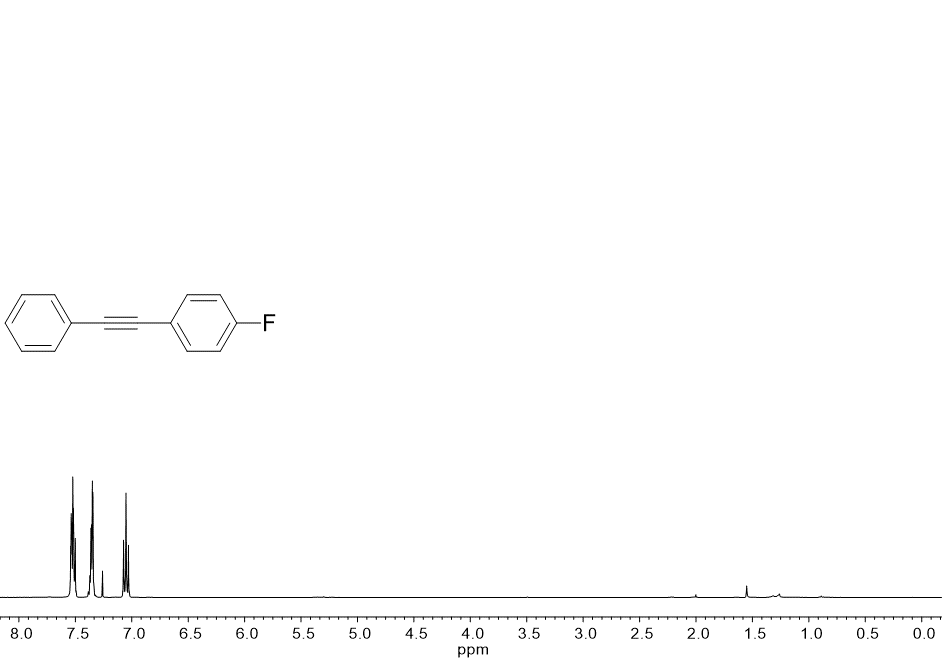


**Figure S45**. ^1^H NMR spectrum (400 MHz, 298 K, CDCl_3_) of **3d**.


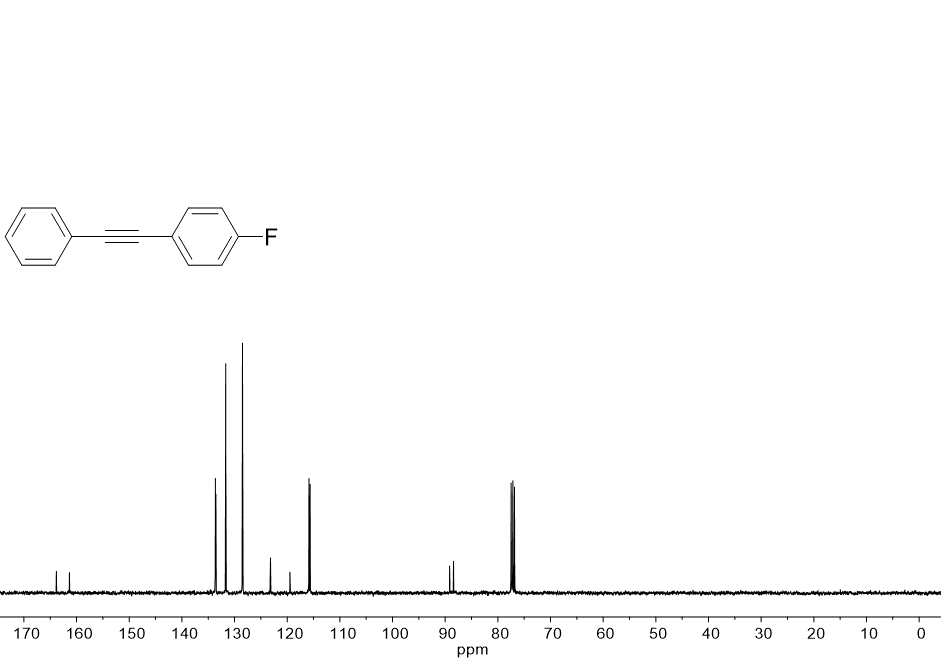


**Figure S46**. ^13^C{^1^H} NMR spectrum (100 MHz, 298 K, CDCl_3_) of **3d**.


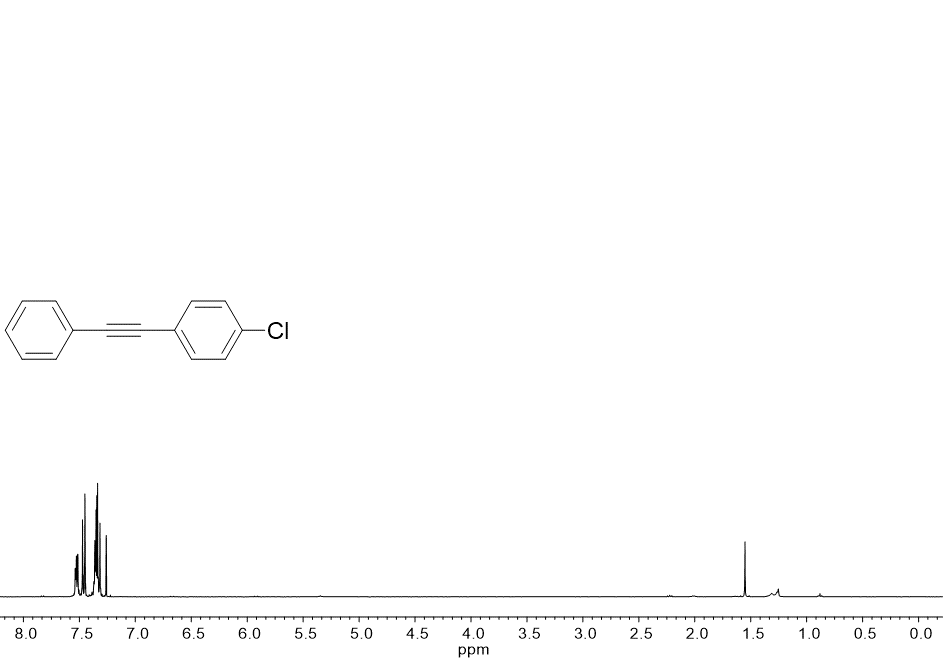


**Figure S47**. ^1^H NMR spectrum (400 MHz, 298 K, CDCl_3_) of **3e**.


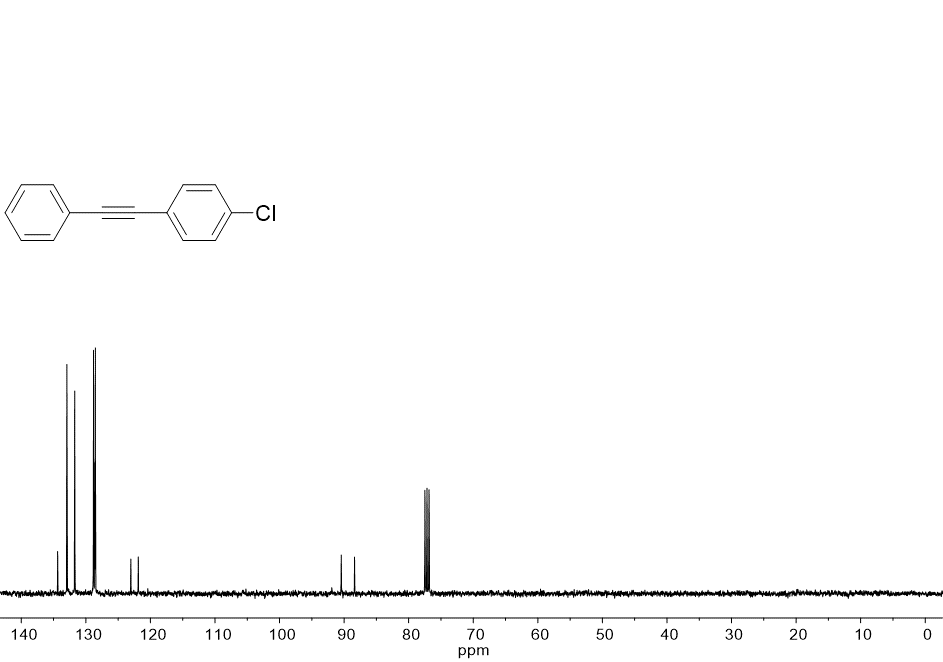


**Figure S48**. ^13^C{^1^H} NMR spectrum (100 MHz, 298 K, CDCl_3_) of **3e**.


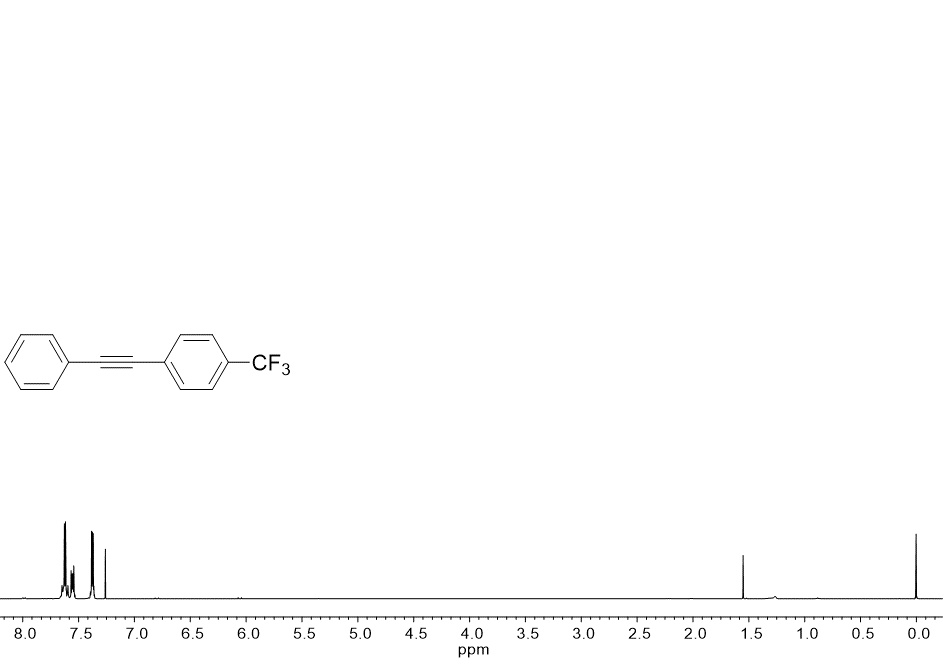


**Figure S49**. ^1^H NMR spectrum (400 MHz, 298 K, CDCl_3_) of **3f**.


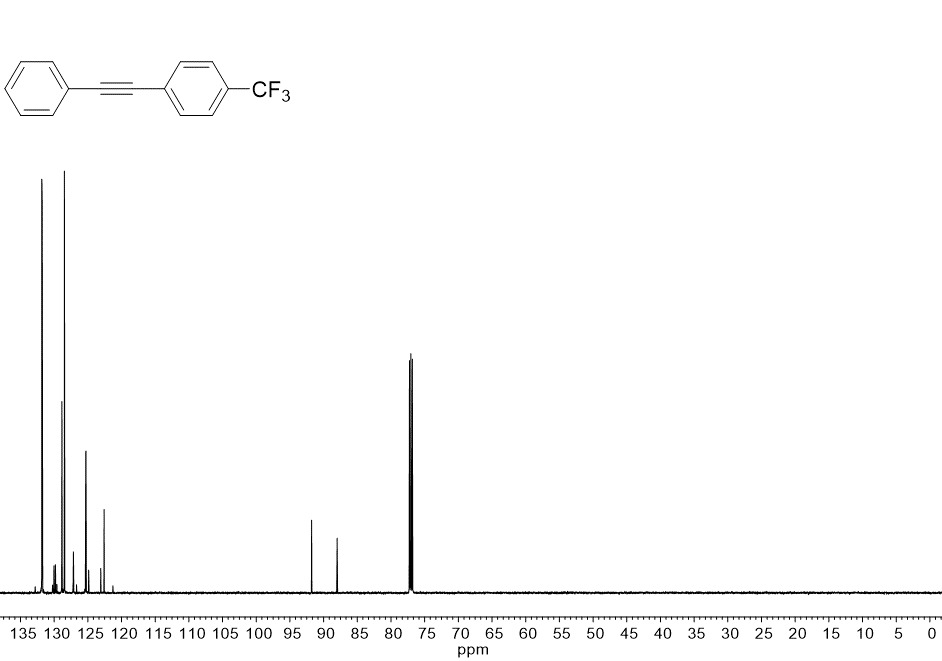


**Figure S50**. ^13^C{^1^H} NMR spectrum (150 MHz, 298 K, CDCl_3_) of **3f**.


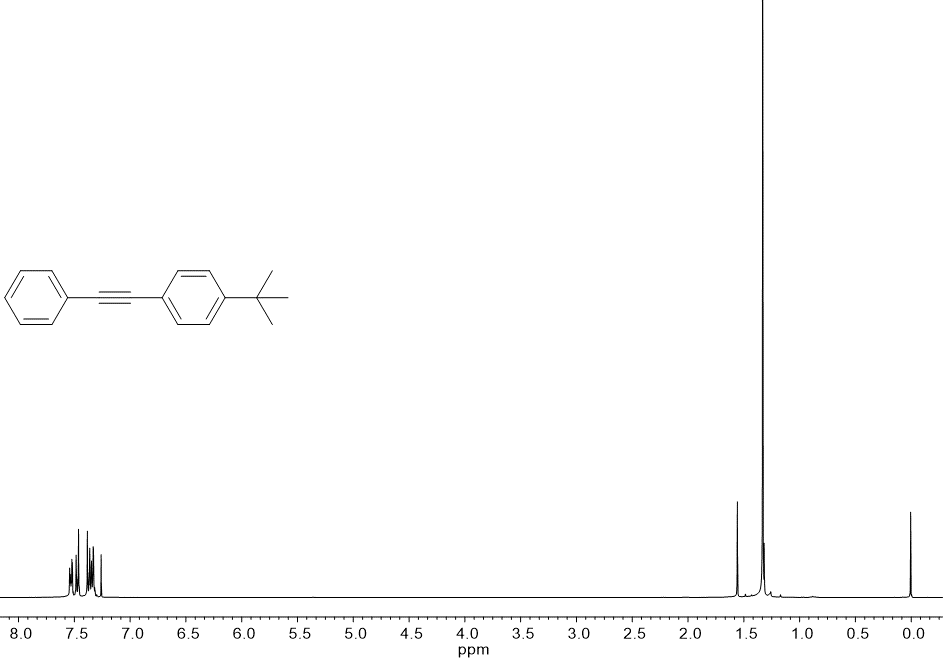


**Figure S51**. ^1^H NMR spectrum (400 MHz, 298 K, CDCl_3_) of **3g**.


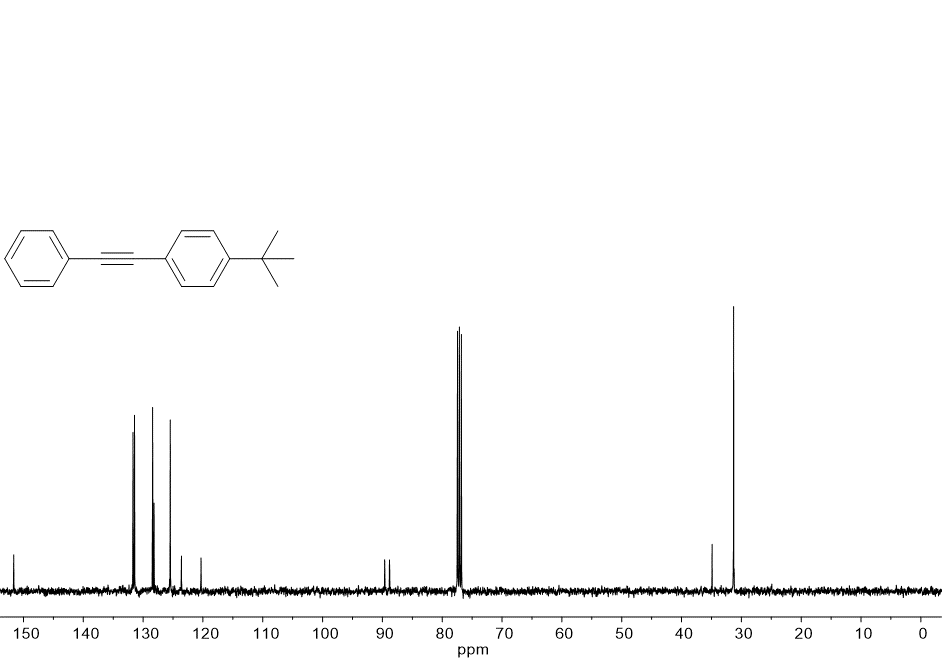


**Figure S52**. ^13^C{^1^H} NMR spectrum (100 MHz, 298 K, CDCl_3_) of **3g**.


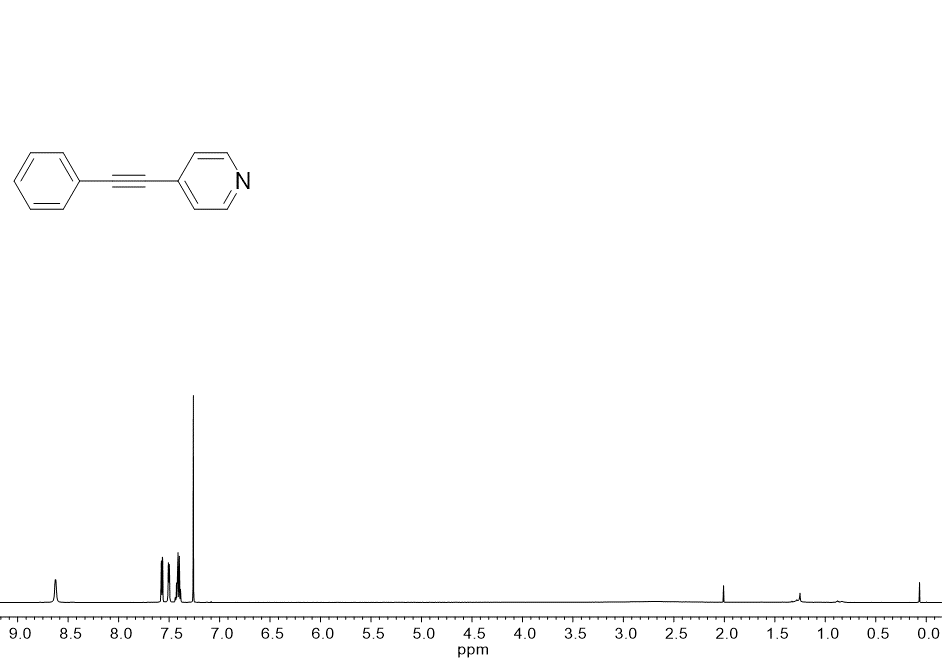


**Figure S53**. ^1^H NMR spectrum (400 MHz, 298 K, CDCl_3_) of **3h**.


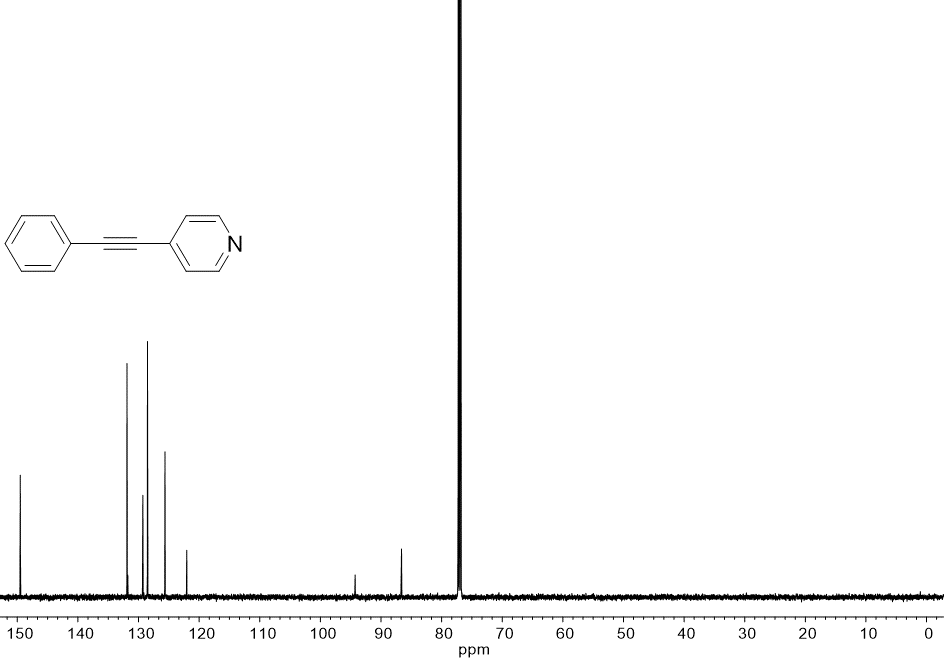


**Figure S54**. ^13^C{^1^H} NMR spectrum (100 MHz, 298 K, CDCl_3_) of **3h**.


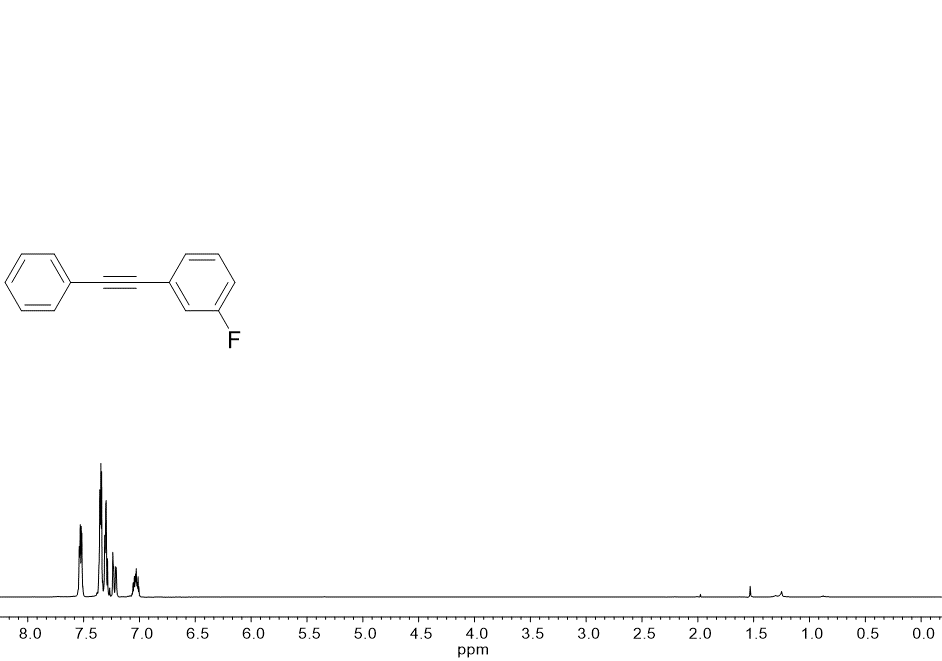


**Figure S55**. ^1^H NMR spectrum (400 MHz, 298 K, CDCl_3_) of **3i**.


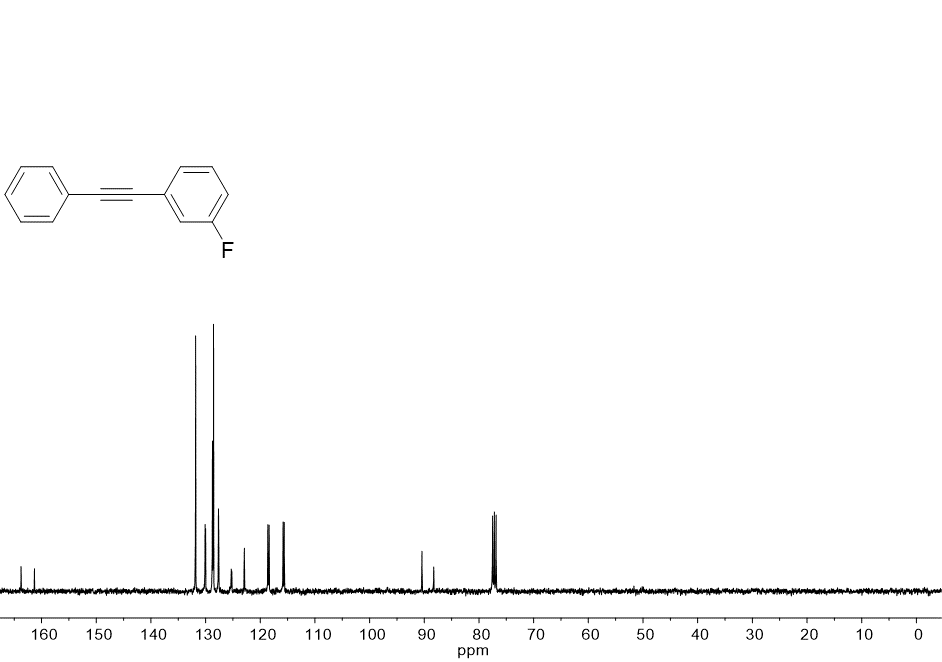


**Figure S56.** ^13^C{^1^H} NMR spectrum (100 MHz, 298 K, CDCl_3_) of **3i**.


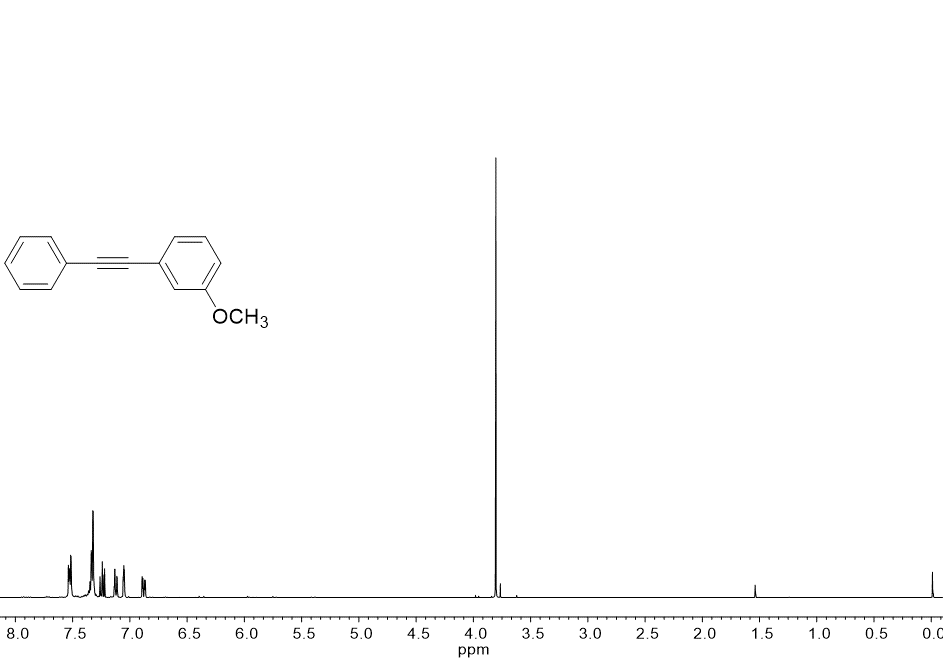


**Figure S57**. ^1^H NMR spectrum (400 MHz, 298 K, CDCl_3_) of **3j**.


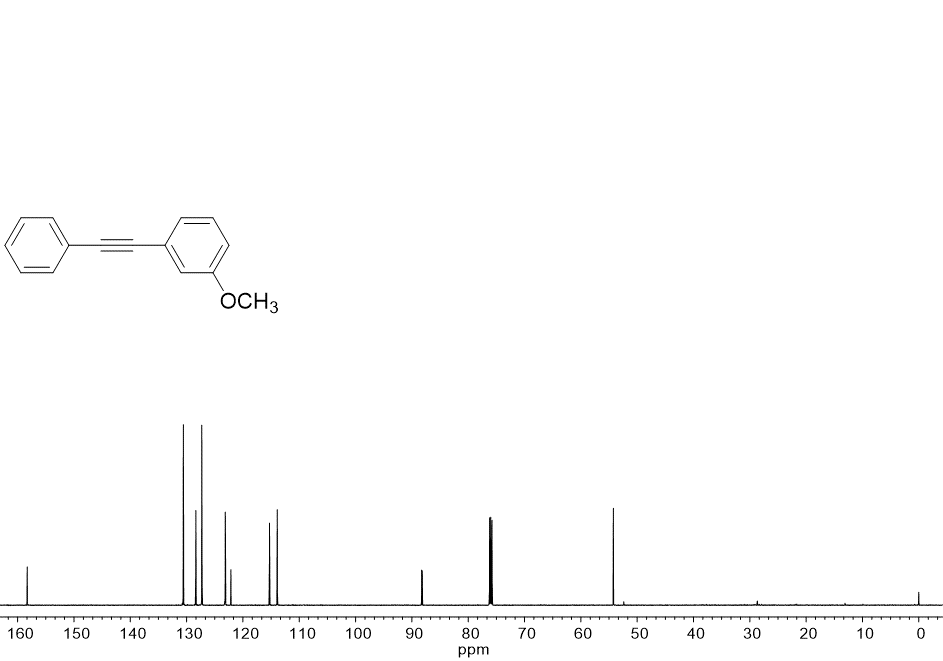


**Figure S58**. ^13^C{^1^H} NMR spectrum (100 MHz, 298 K, CDCl_3_) of **3j.**


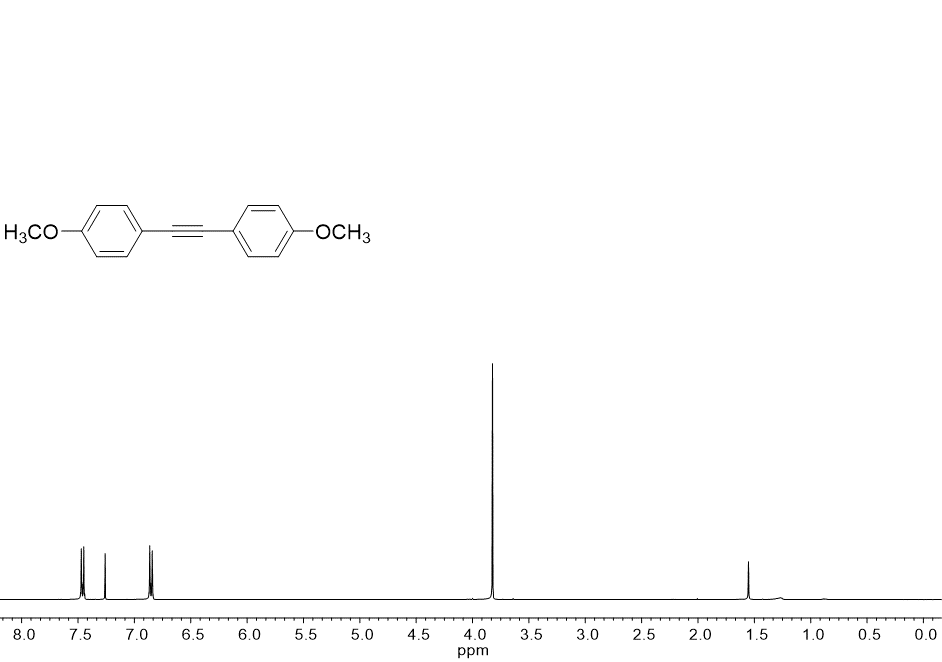


**Figure S59**. ^1^H NMR spectrum (400 MHz, 298 K, CDCl_3_) of **3k**.


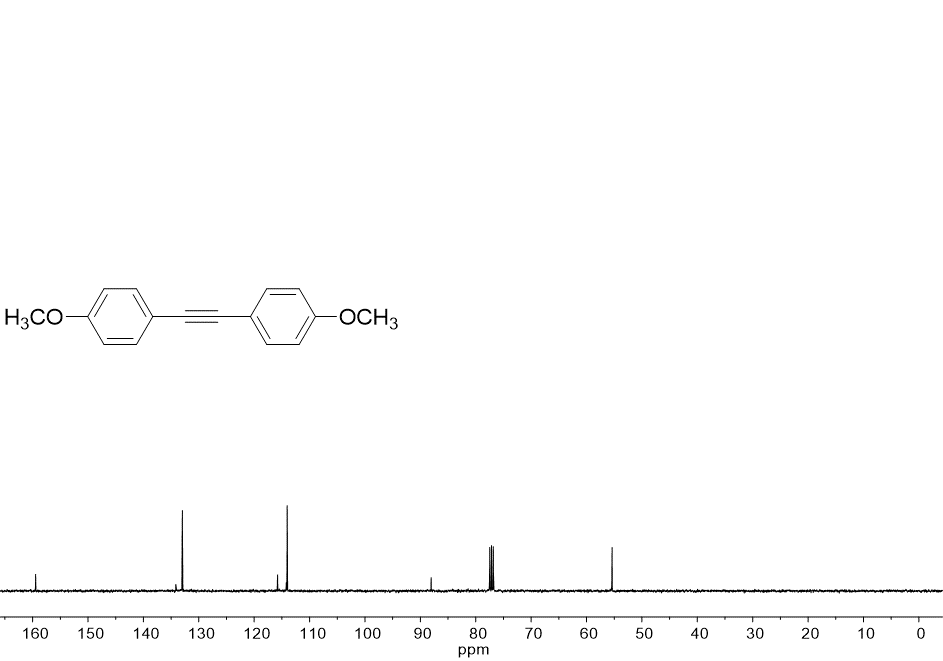


**Figure S60.** ^13^C{^1^H} NMR spectrum (100 MHz, 298 K, CDCl_3_) of **3k**.


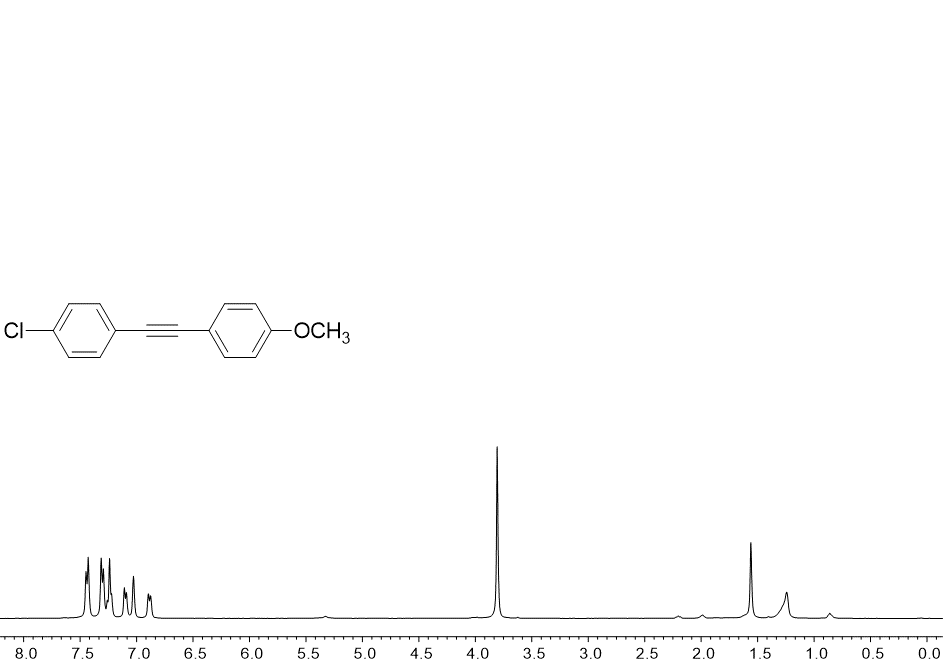


**Figure S61**. ^1^H NMR spectrum (400 MHz, 298 K, CDCl_3_) of **3l**.


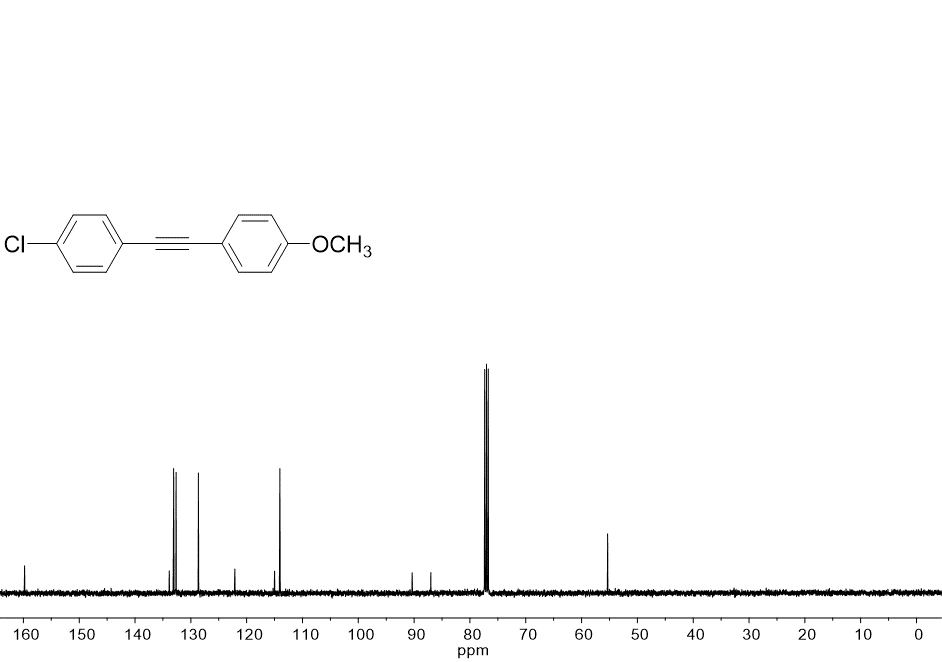


**Figure S62**. ^13^C{^1^H} NMR spectrum (100 MHz, 298 K, CDCl_3_) of **3l**.


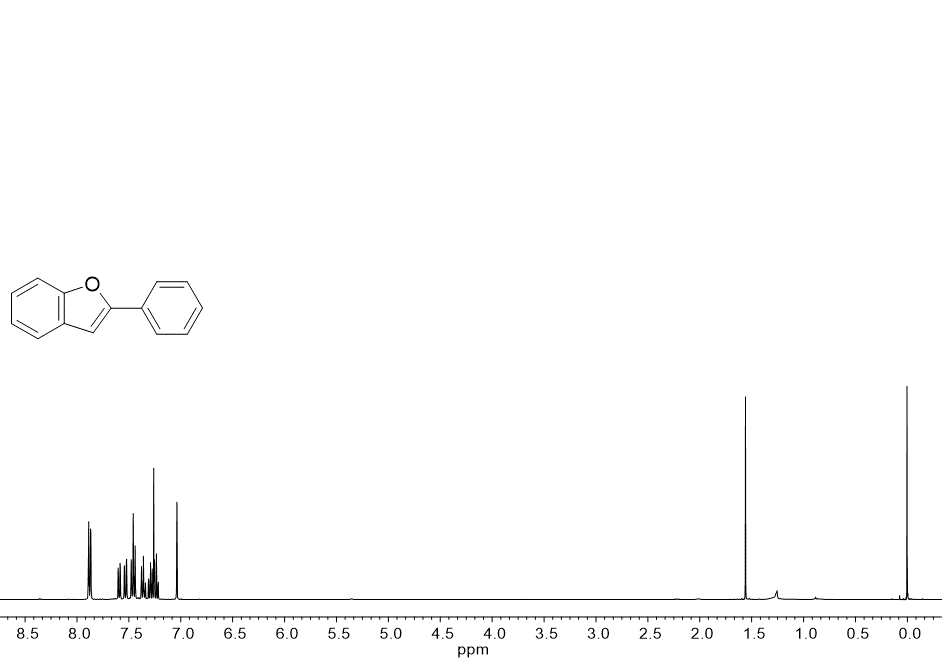


**Figure S63**. ^1^H NMR spectrum (400 MHz, 298 K, CDCl_3_) of **6a**.


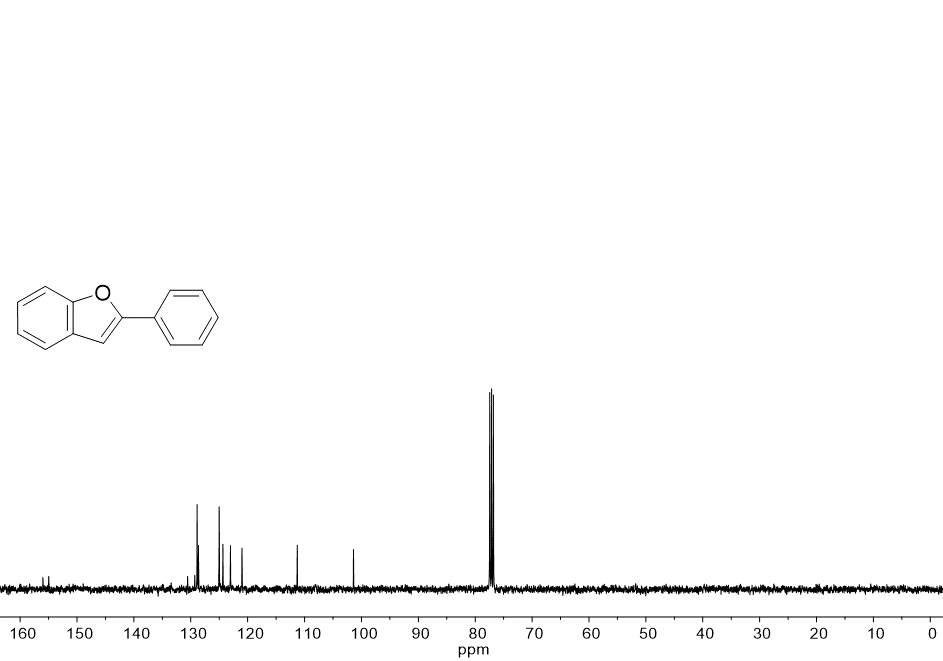


**Figure S64**. ^13^C{^1^H} NMR spectrum (100 MHz, 298 K, CDCl_3_) of **6a**.


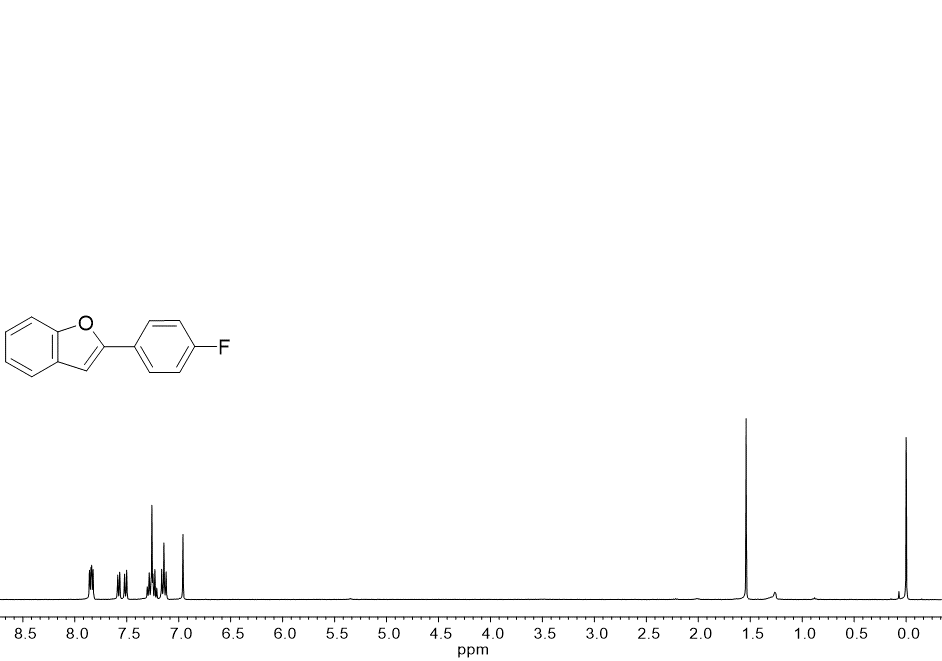


**Figure S65**. ^1^H NMR spectrum (400 MHz, 298 K, CDCl_3_) of **6b**.


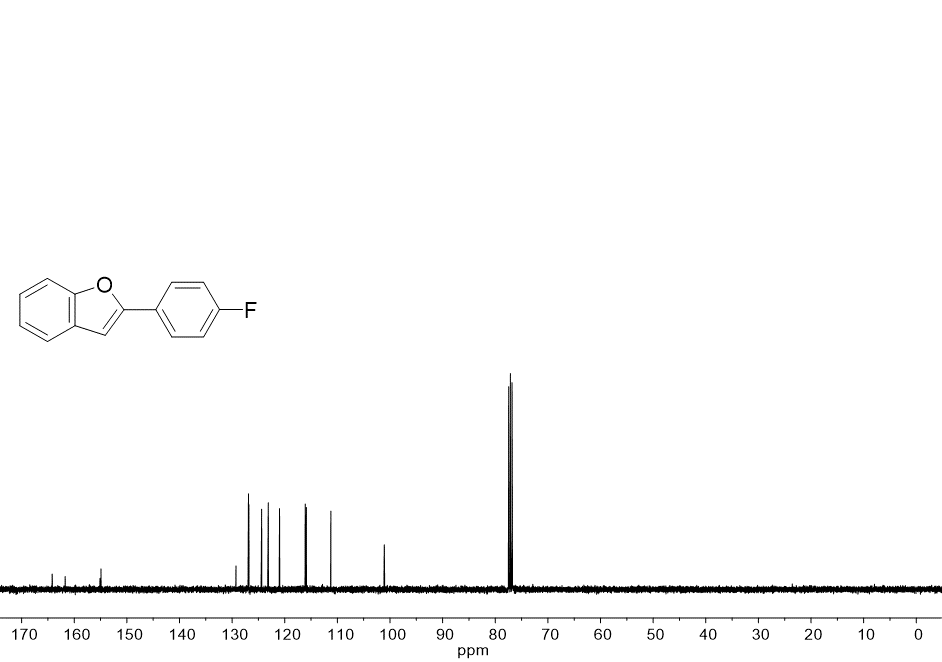


**Figure S66** ^13^C{^1^H} NMR spectrum (100 MHz, 298 K, CDCl_3_) of **6b**.


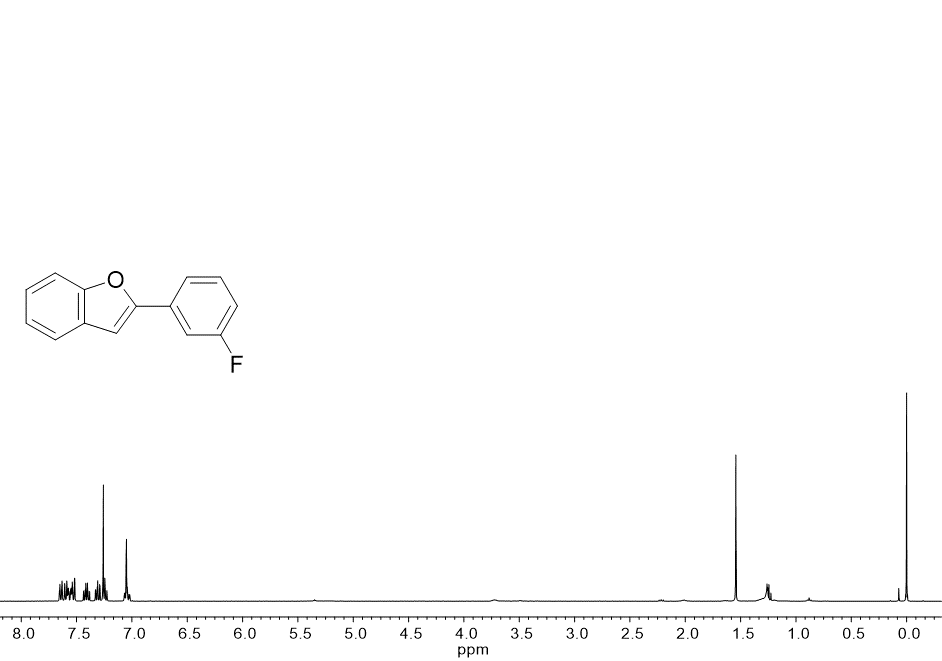


**Figure S67**. ^1^H NMR spectrum (400 MHz, 298 K, CDCl_3_) of **6c**.


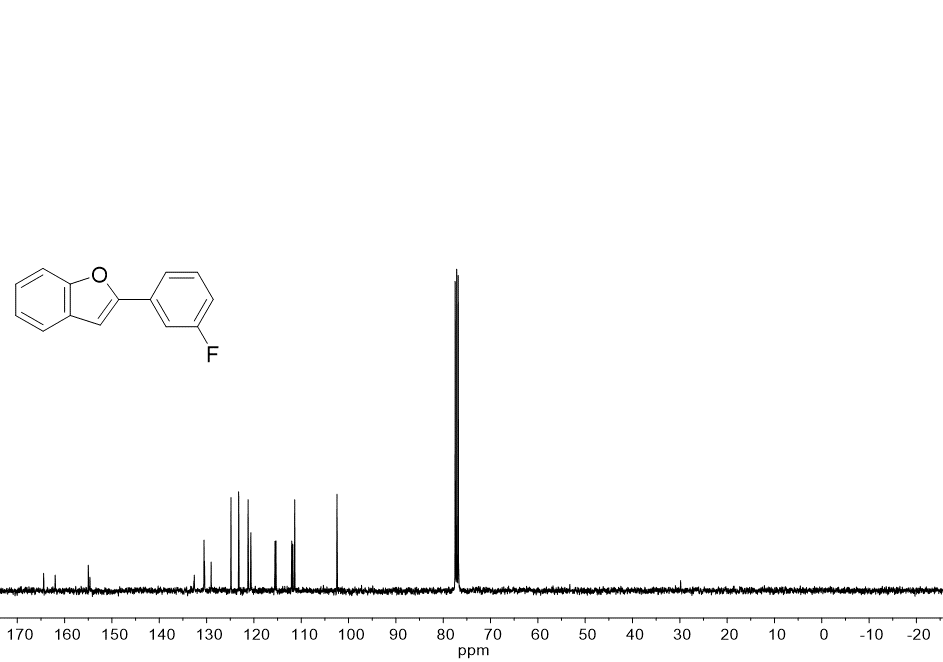


**Figure S68**. ^13^C{^1^H} NMR spectrum (100 MHz, 298 K, CDCl_3_) of **6c**.


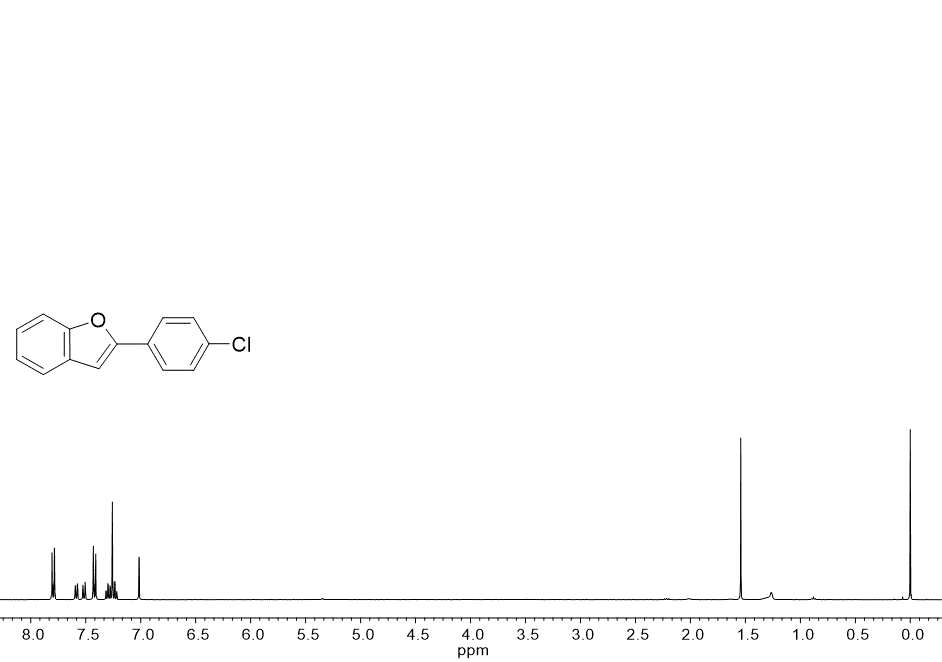


**Figure S69**. ^1^H NMR spectrum (400 MHz, 298 K, CDCl_3_) of **6d**.


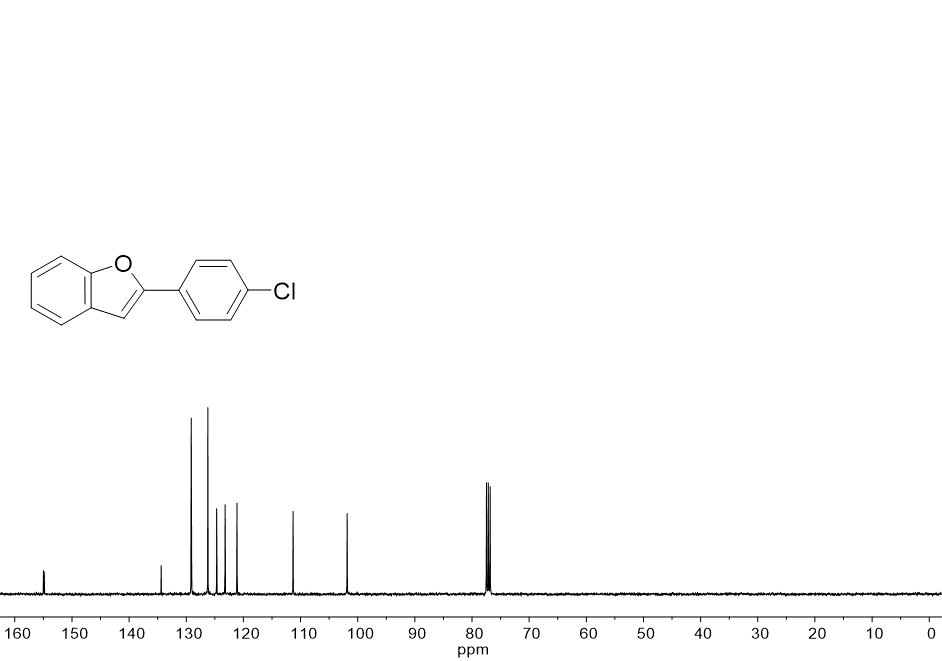


**Figure S70**. ^13^C{^1^H} NMR spectrum (100 MHz, 298 K, CDCl_3_) of **6d**.


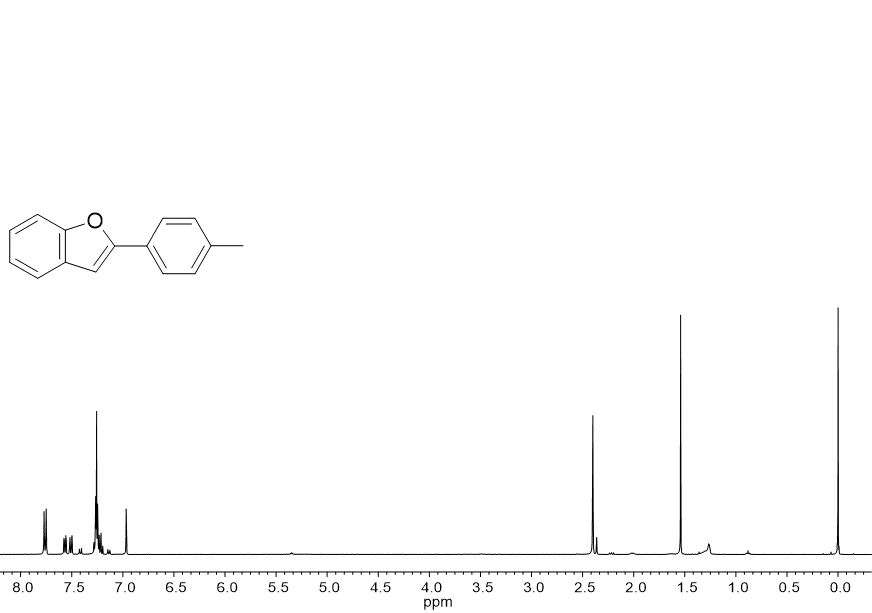


**Figure S71**. ^1^H NMR spectrum (400 MHz, 298 K, CDCl_3_) of **6e**.


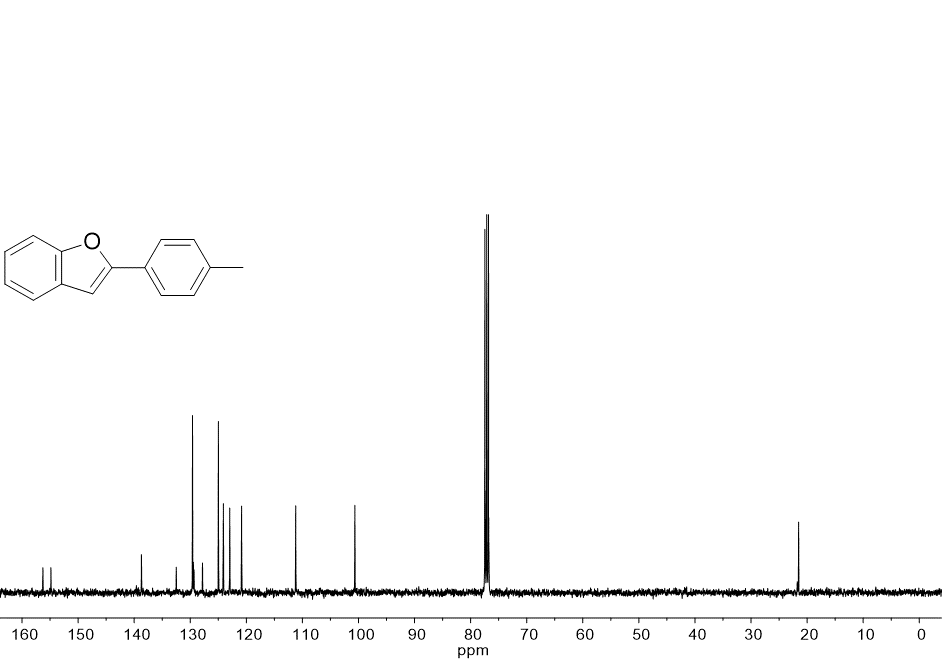


**Figure S72**. ^13^C{^1^H} NMR spectrum (100 MHz, 298 K, CDCl_3_) of **6e**.


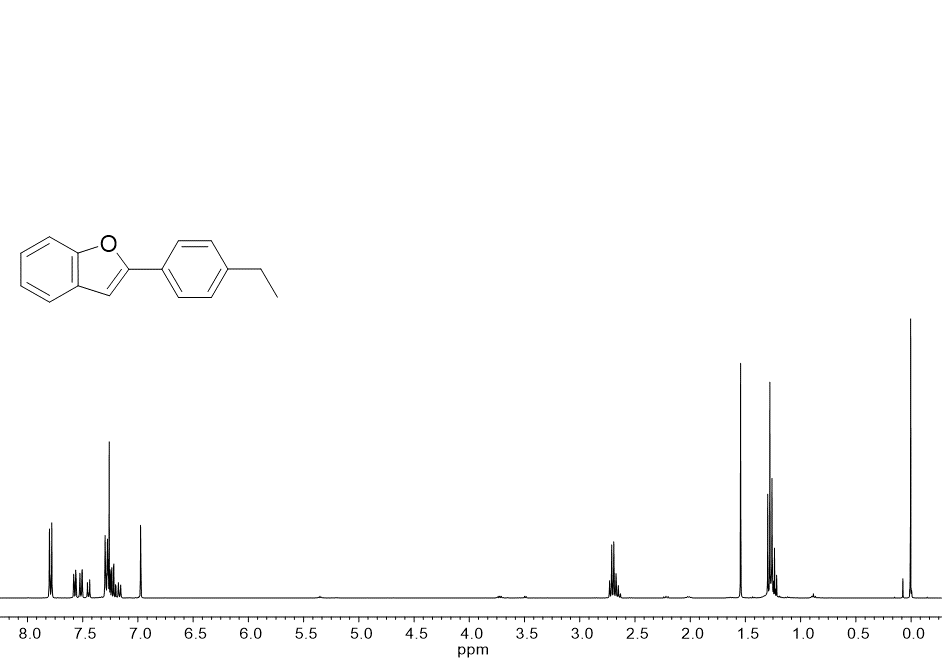


**Figure S73**. ^1^H NMR spectrum (400 MHz, 298 K, CDCl_3_) of **6f**.


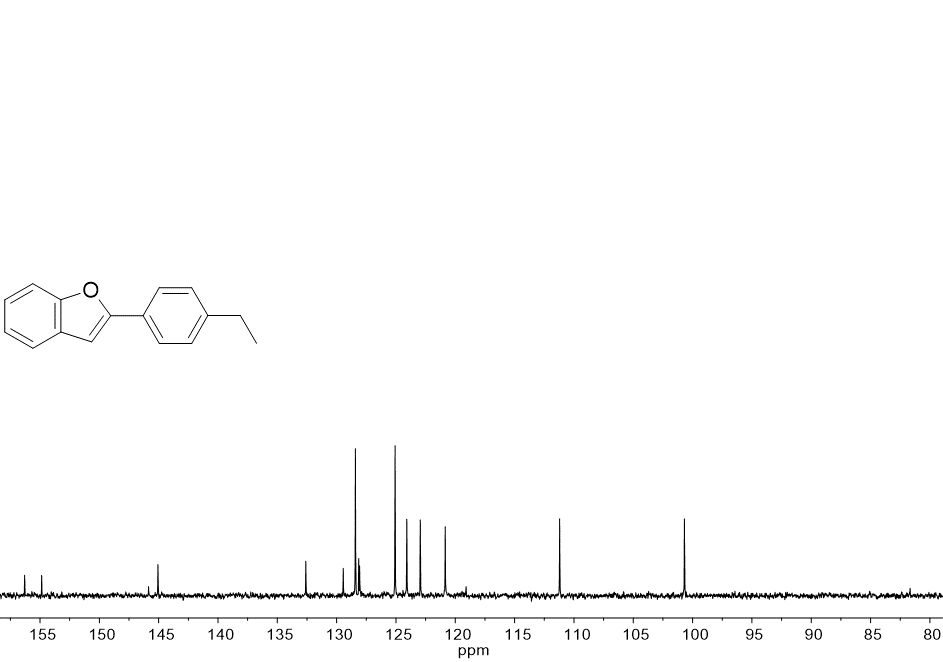


**Figure S74**. ^13^C{^1^H} NMR spectrum (100 MHz, 298 K, CDCl_3_) of **6f**.


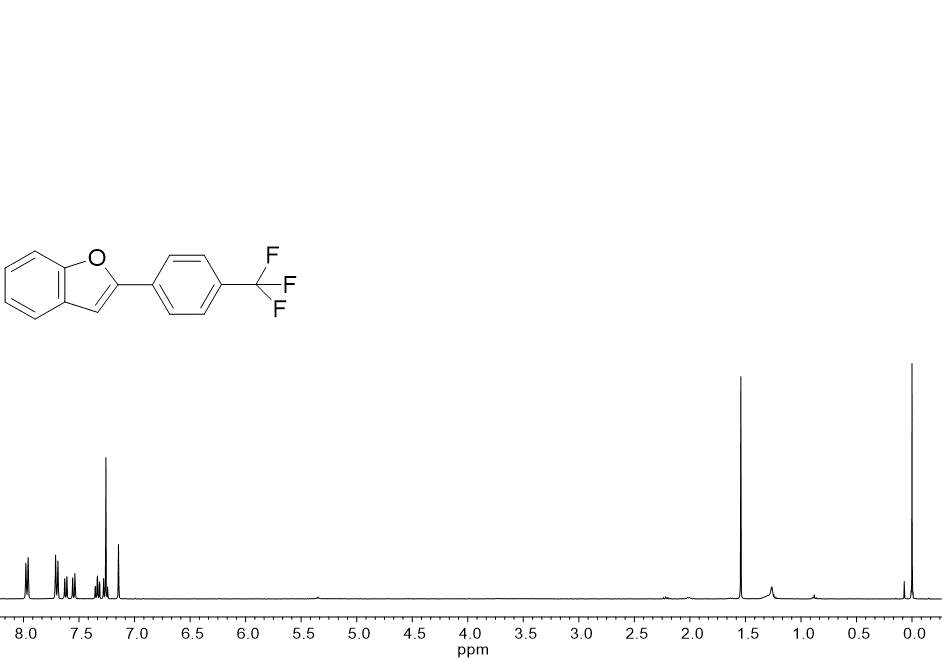


**Figure S75**. ^1^H NMR spectrum (400 MHz, 298 K, CDCl_3_) of **6g**.


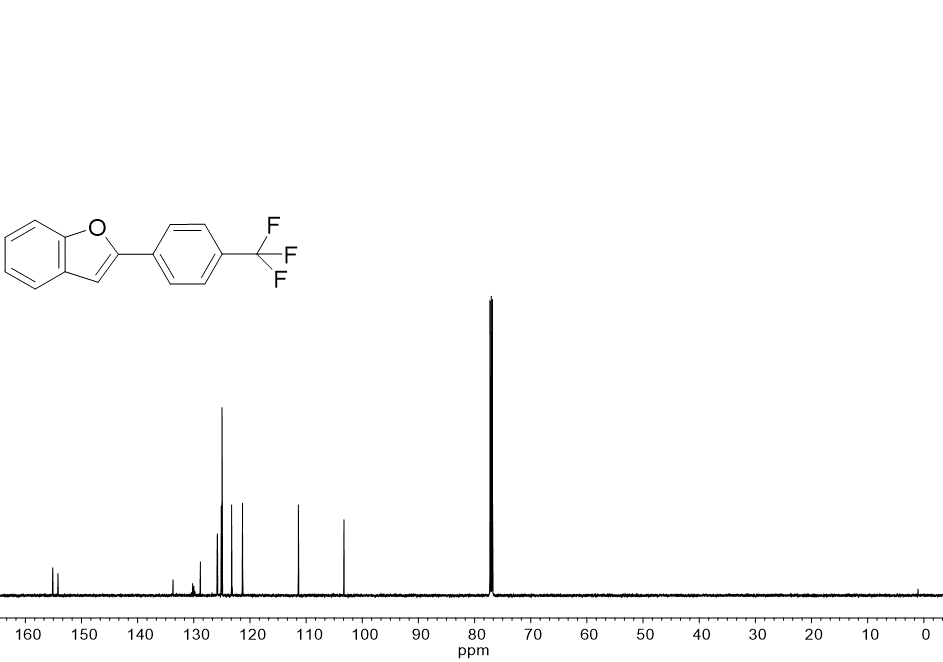


**Figure S76**. ^13^C{^1^H} NMR spectrum (150 MHz, 298 K, CDCl_3_) of **6g**.


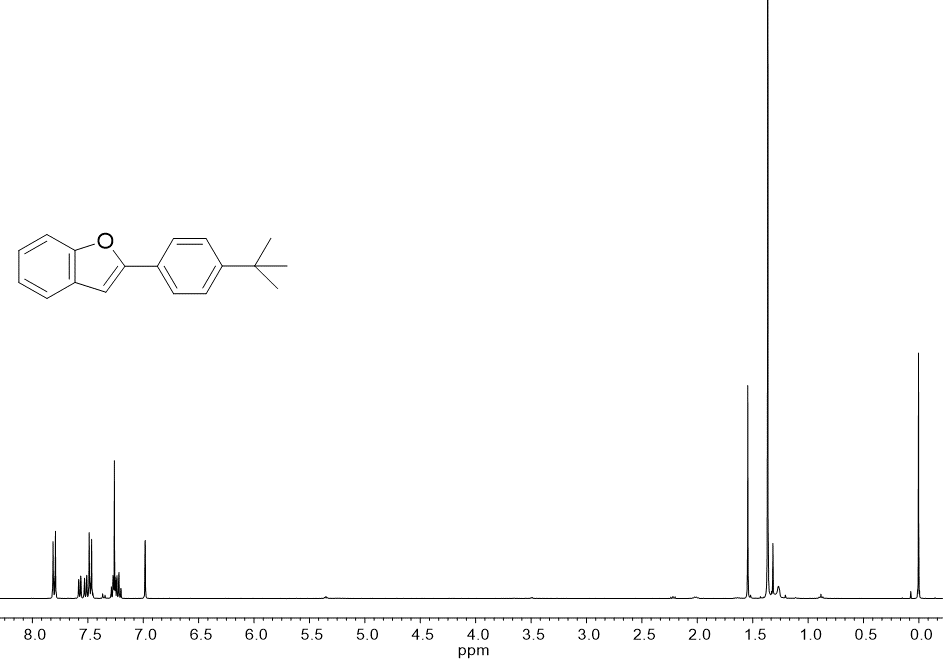


**Figure S77**. ^1^H NMR spectrum (400 MHz, 298 K, CDCl_3_) of **6h**.


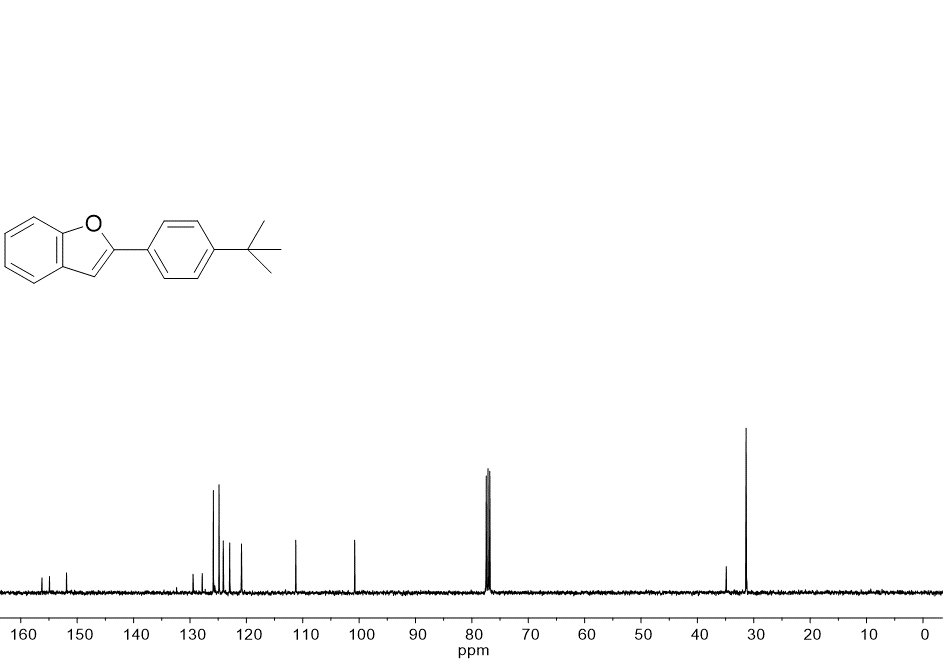


**Figure S78**. ^13^C{^1^H} NMR spectrum (100 MHz, 298 K, CDCl_3_) of **6h**.


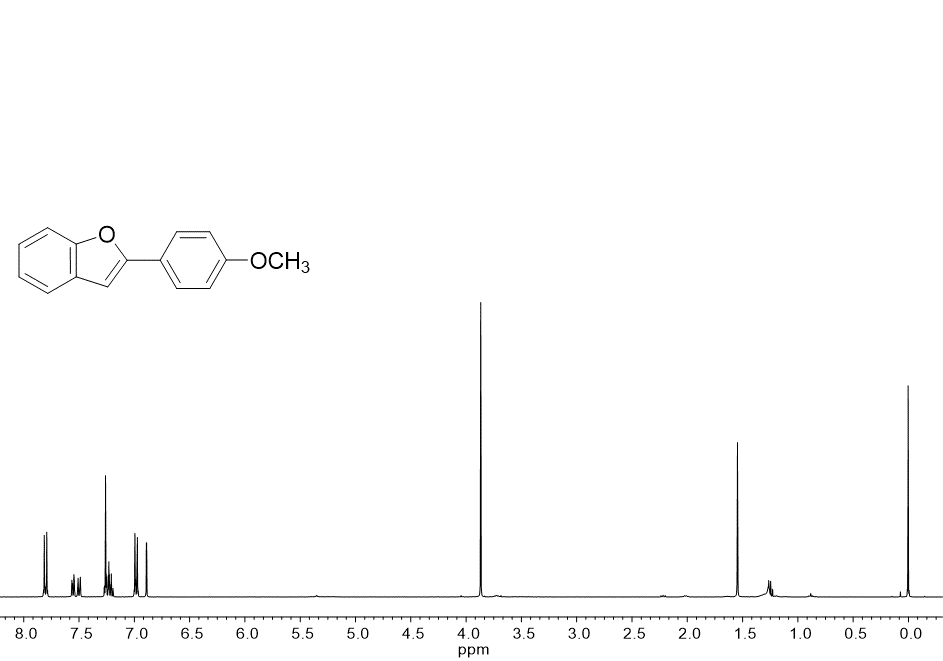


**Figure S79**. ^1^H NMR spectrum (400 MHz, 298 K, CDCl_3_) of **6i**.


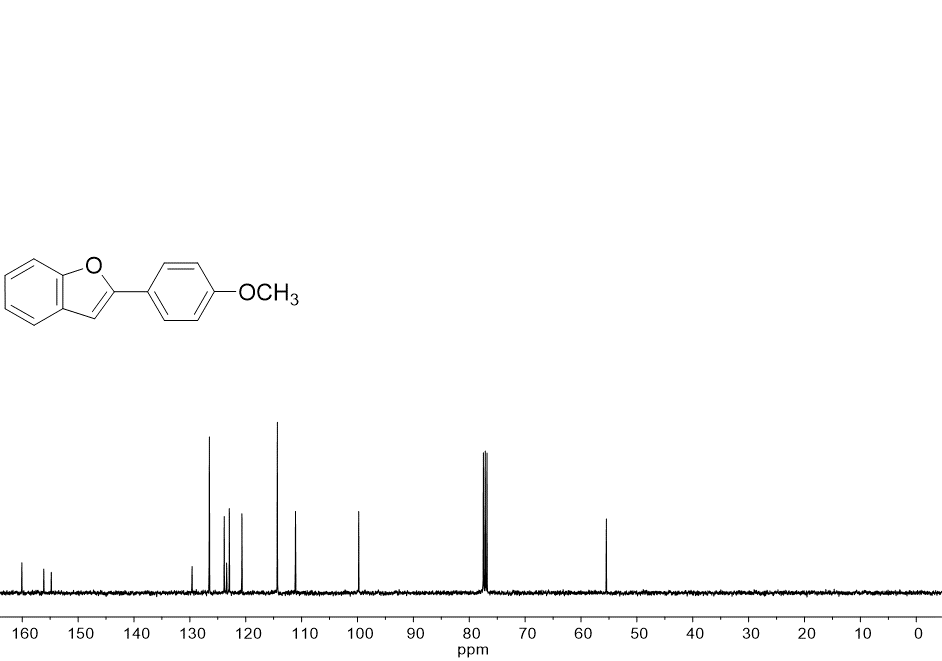


**Figure S80**. ^13^C{^1^H} NMR spectrum (100 MHz, 298 K, CDCl_3_) of **6i**.


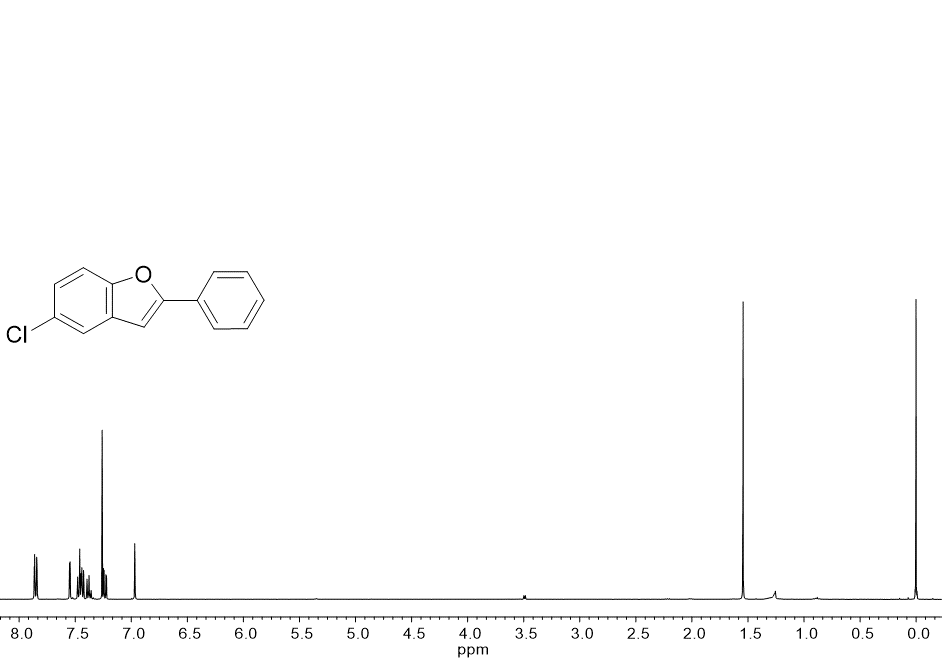


**Figure S81**. ^1^H NMR spectrum (400 MHz, 298 K, CDCl_3_) of **6j**.


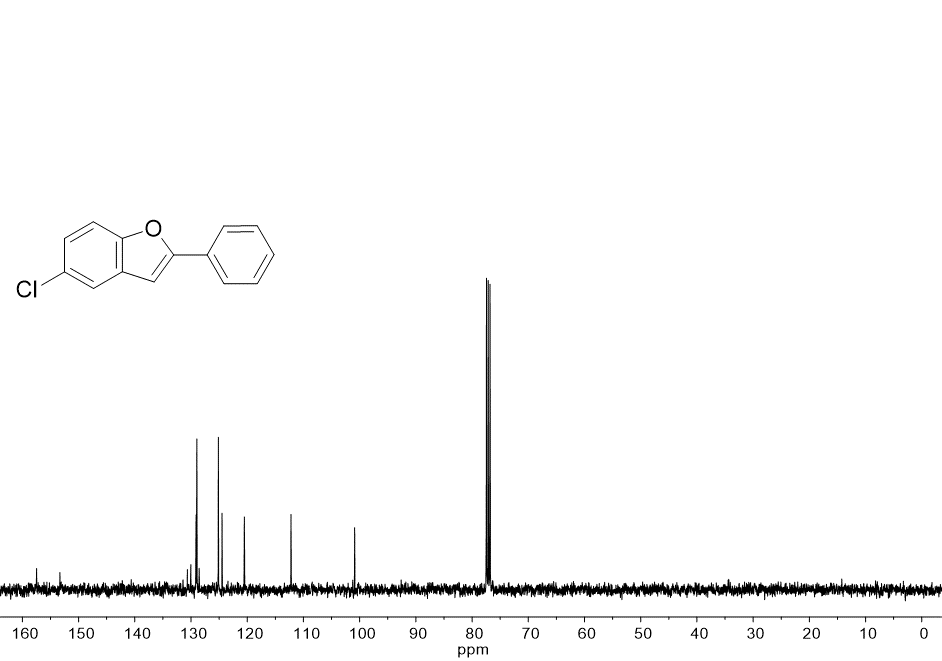


**Figure S82**. ^13^C{^1^H} NMR spectrum (100 MHz, 298 K, CDCl_3_) of **6j**.


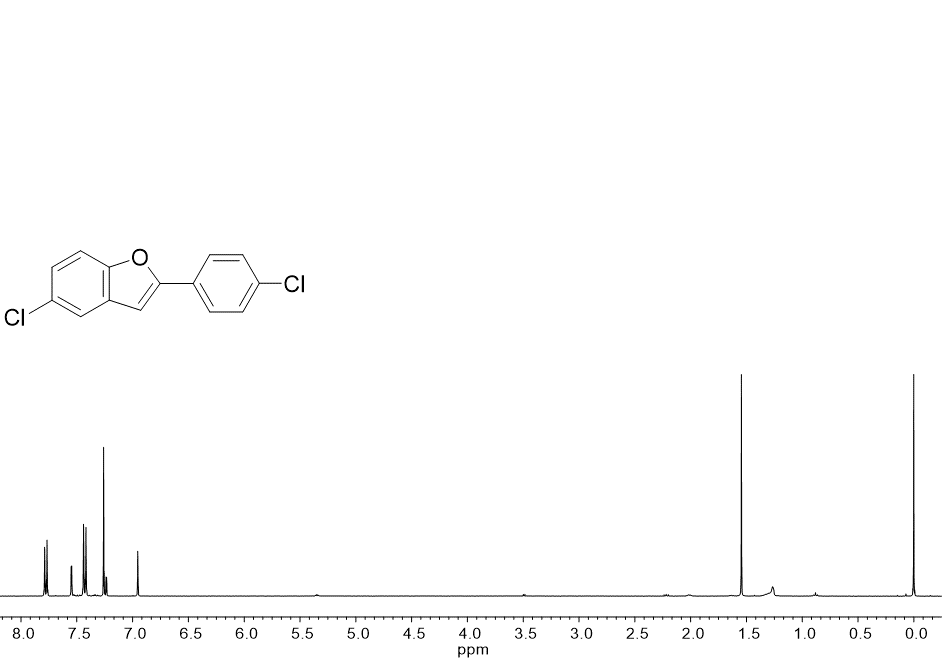


**Figure S83**. ^1^H NMR spectrum (400 MHz, 298 K, CDCl_3_) of **6k**.


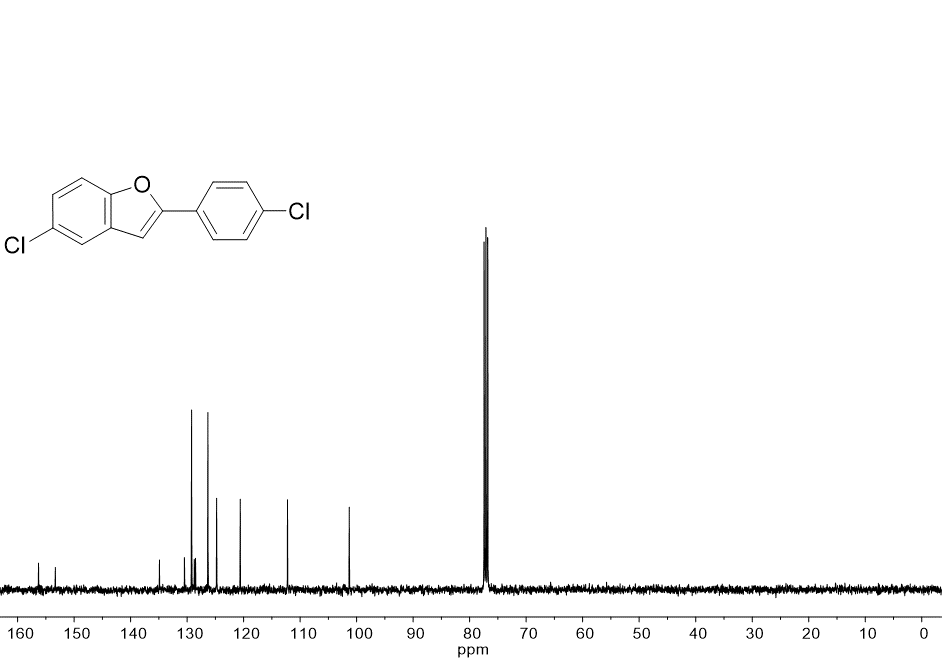


**Figure S84**. ^13^C{^1^H} NMR spectrum (100 MHz, 298 K, CDCl_3_) of **6k**.


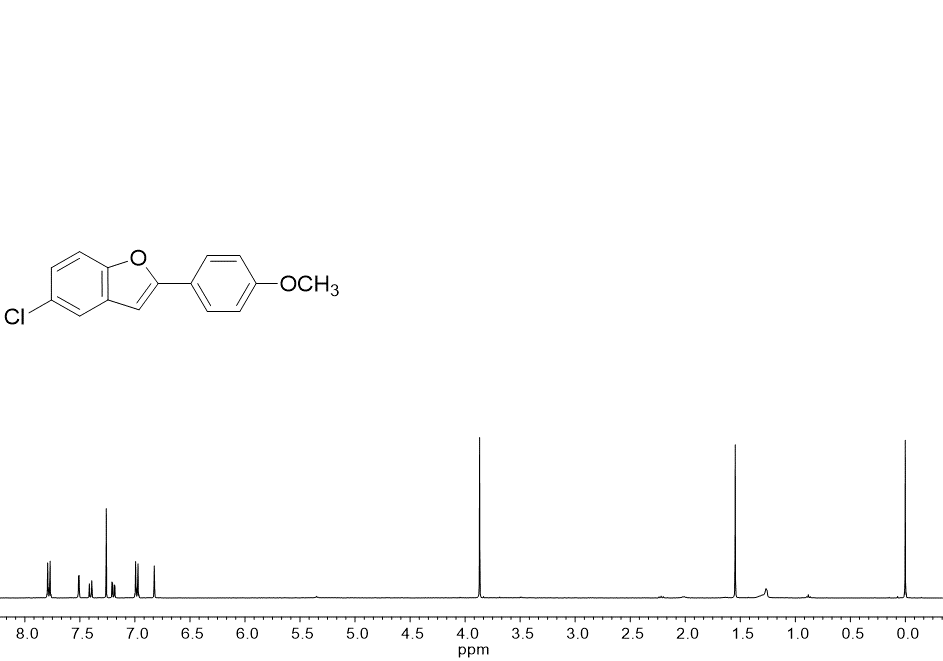


**Figure S85**. ^1^H NMR spectrum (400 MHz, 298 K, CDCl_3_) of **6l**.


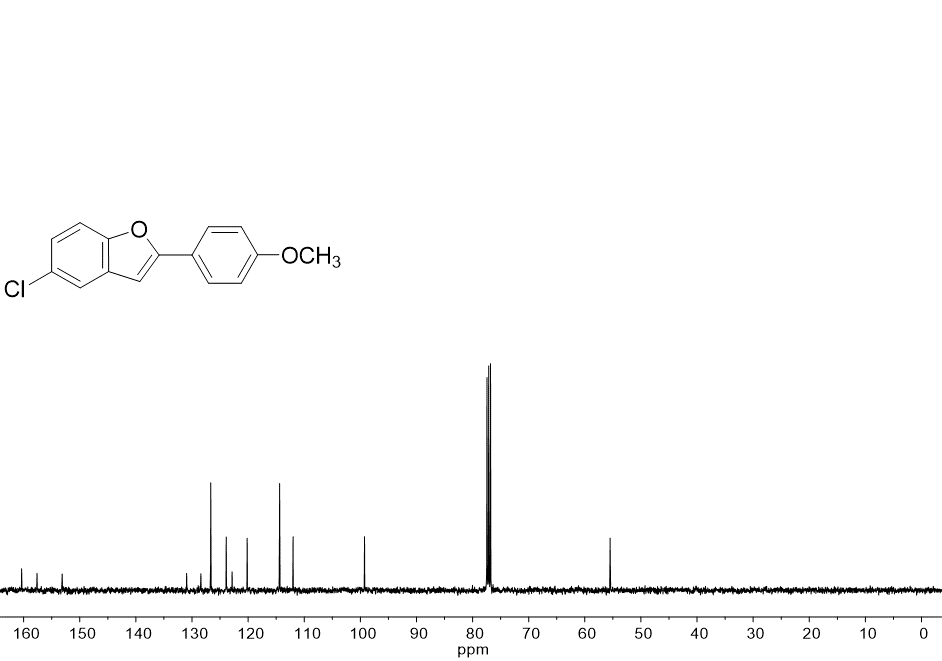


**Figure S86**. ^13^C{^1^H} NMR spectrum (100 MHz, 298 K, CDCl_3_) of **6l**.


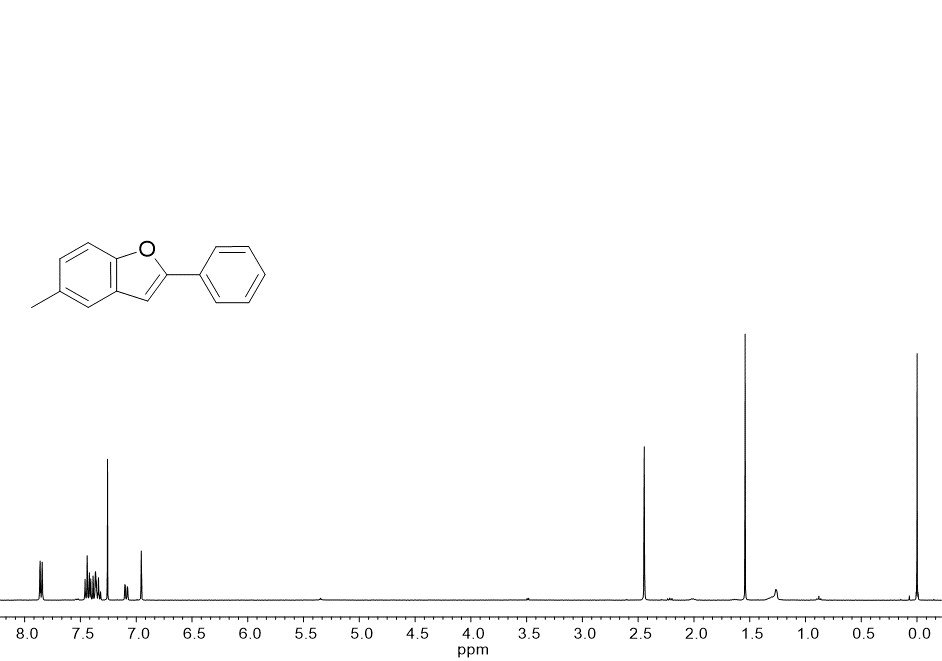


**Figure S87**. ^1^H NMR spectrum (400 MHz, 298 K, CDCl_3_) of **6m**.


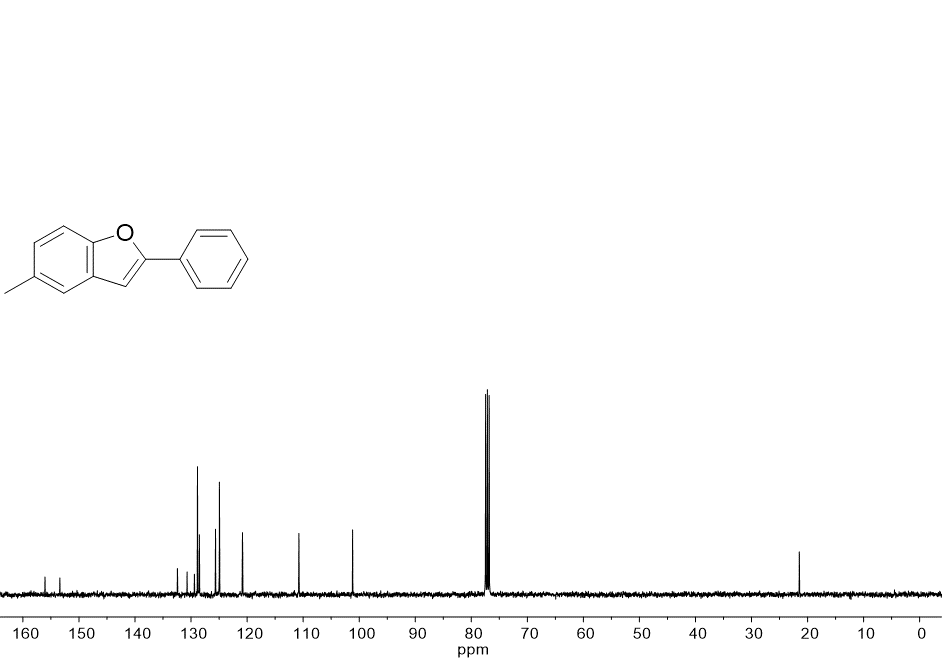


**Figure S88**. ^13^C{^1^H} NMR spectrum (100 MHz, 298 K, CDCl_3_) of **6m**.


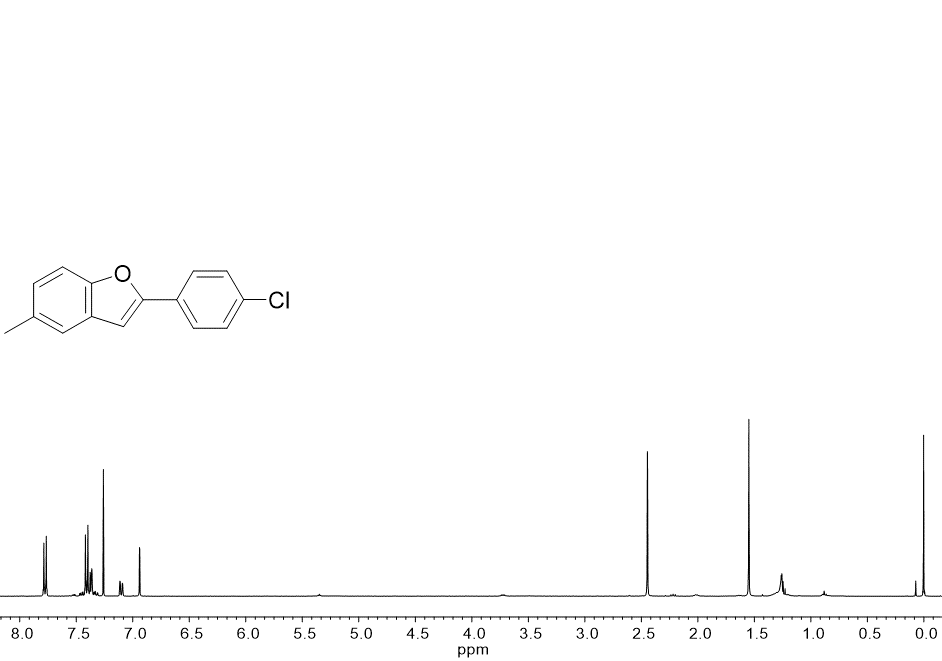


**Figure S89**. ^1^H NMR (spectrum 400 MHz, 298 K, CDCl_3_) of **6n**.


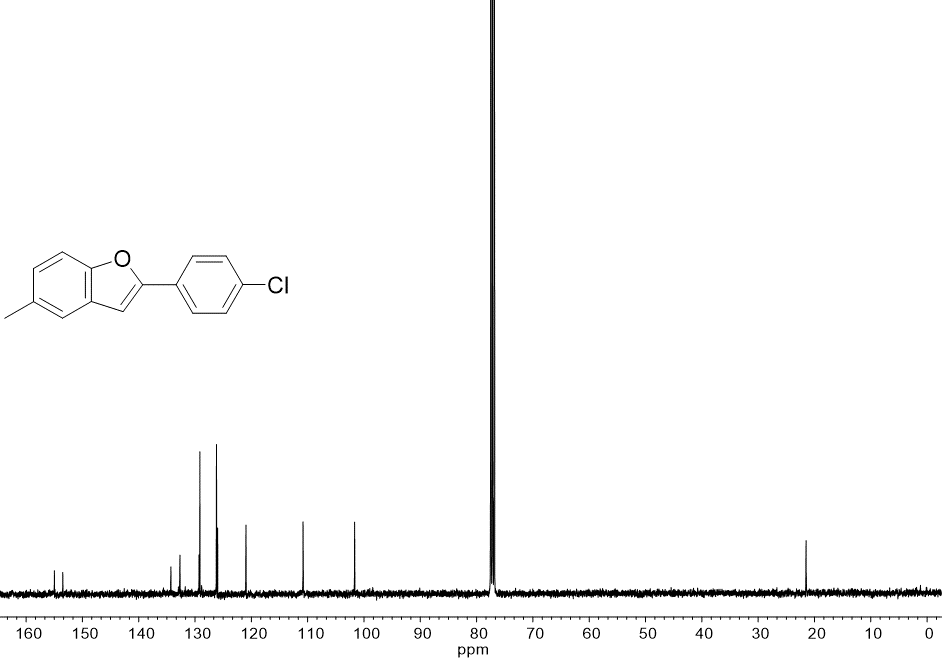


**Figure S90**. ^13^C{^1^H} NMR spectrum (100 MHz, 298 K, CDCl_3_) of **6n**.


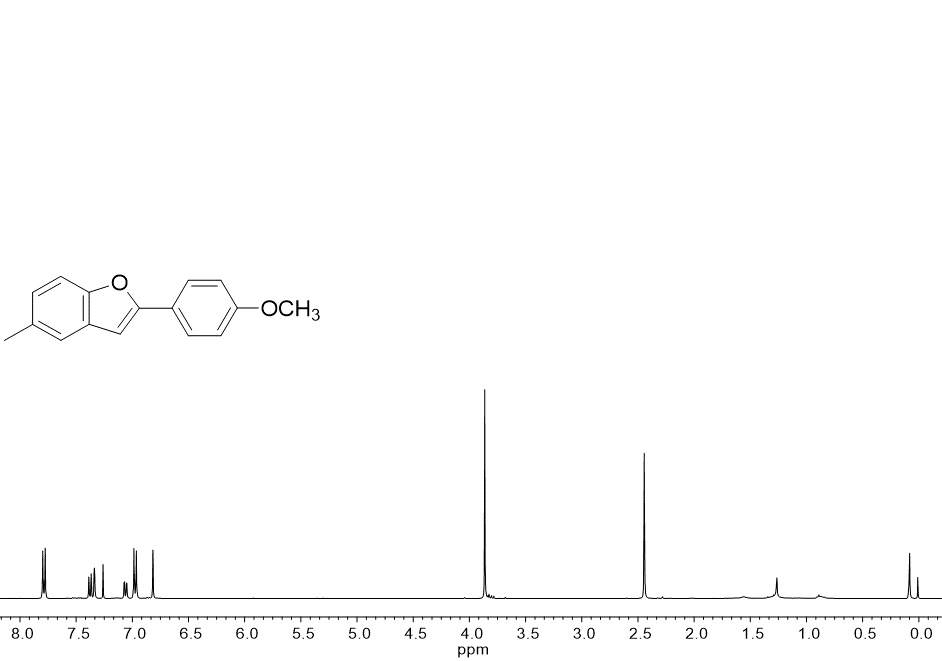


**Figure S91**. ^1^H NMR (spectrum 400 MHz, 298 K, CDCl_3_) of **6o**.


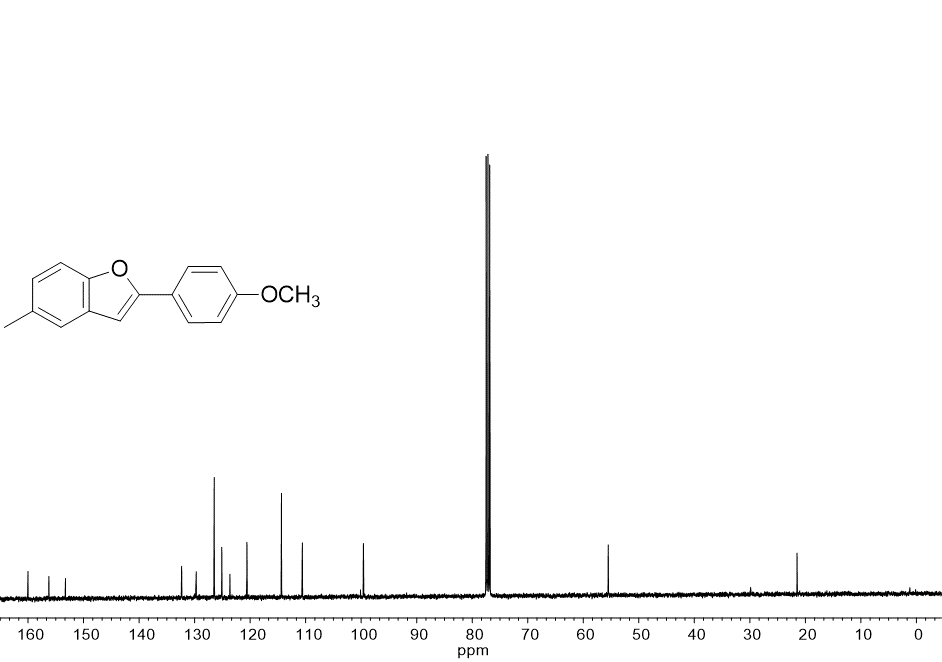


**Figure S92**. ^13^C{^1^H} NMR spectrum (100 MHz, 298 K, CDCl_3_) of **6o**.

# 8. References

1. Wang W, Cui L and Sun P *et al.* Reusable N-Heterocyclic Carbene Complex Catalysts and Beyond: A Perspective on Recycling Strategies. *Chem Rev* 2018; **118**: 9843−929.

2. Biffis A, Centomo P and Zotto AD *et al.* Pd Metal Catalysts for Cross-Couplings and Related Reactions in the 21st Century: A Critical Review. *Chem Rev* 2018; **118**: 2249−95.

3. Truong T and Daugulis, O. Transition-Metal-Free Alkynylation of Aryl Chlorides. *Org Lett* 2011; **13**: 4172–5.

4. Ruengsangtongkul S, Chaisan N and Thongsornkleeb C *et al.* Rate Enhancement in CAN-Promoted Pd(PPh_3_)_2_Cl_2_-Catalyzed Oxidative Cyclization: Synthesis of 2-Ketofuran-4-carboxylate Esters. *Org Lett* 2019; **21**: 2514–7.

5. Sagadevan A and Hwang KC. Photo-Induced Sonogashira C-C Coupling Reaction Catalyzed by Simple Copper(I) Chloride Salt at Room Temperature. *Adv Synth Catal* 2012; **354**: 3421–7.

6. He J, Yang K and Zhao J *et al.* LiHMDS-Promoted Palladium-Catalyzed Sonogashira Cross-Coupling of Aryl Fluorides with Terminal Alkynes. *Org Lett* 2019; **21**: 9714–8.

7. Duan Y, Zhang S and Yang Y. Synthesis of Benzofurans from Terminal Alkynes and Iodophenols Catalyzed by Recyclable Palladium Nanoparticles Supported on N, O-dual Doped Hierarchical Porous Carbon Under Copper- and Ligand-Free Conditions. *Catal Today* 2019; **330**: 101–8.

8. Singh FV and Mangaonkar SR. Hypervalent Iodine(III)-Catalyzed Synthesis of 2-Arylbenzofurans. *Synthesis* 2018; **50**: 4940–8.

9. Qin DD, Chen W and Tang X *et al*. Accessing 2-Arylbenzofurans by Cu^I^_2_(pip)_2_-Catalyzed Tandem Coupling/Cyclization Reaction: Mechanistic Studies and Application to the Synthesis of Stemofuran A and Moracin M. *Asian J Org Chem* 2016; **5**: 1345–52.

10. Xu Z, Xu Y and Lu HY *et al*. Efficient and C2-selective arylation of indoles, benzofurans, and benzothiophenes with iodobenzenes in water at room temperature. *Tetrahedron* 2015; **71**: 2616–21.

11. Bhadra M, Sasmal HS and Basu A *et al*. Predesigned Metal-Anchored Building Block for In Situ Generation of Pd Nanoparticles in Porous Covalent Organic Framework: Application in Heterogeneous Tandem Catalysis. *ACS Appl Mater Inter* 2017; **9**: 13785–92.

12. Rao MLN and Dasgupta PA. Concise Route to Functionalized Benzofurans Directly from Gem-dibromoalkenes and Phenols. *RSC Adv* 2015; **5**: 65462–70.

13. Chen ZP, Zhou Y and Shui MZ *et al*. CuI catalyzed domino coupling–cyclization of 2-iodo-phenols and 1-alkynes to the synthesis of 2-substituted benzo[*b*]furans/furo-pyridines. *Tetrahedron Lett* 2019; **60**: 133–6.

14. Gaussian 09, Revision D.01, Frisch MJ, Trucks GW and Schlegel HB *Gaussian Inc* Wallingford CT, 2009.

15. Becke AD. Density-Functional Thermochemistry. III. The Role of Exact Exchange. *J Chem Phys* 1993; **98**: 5648–52.

16. Binkley JS, Pople JA and Hehre WJ. Self-Consistent Molecular Orbital Methods. 21. Small Split-Valence Basis Sets for First-Row Elements. *J Am Chem Soc* 1980; **102**: 939–47.

17. McCaffrey R, Long H and Jin Y *et al*. Template Synthesis of Gold Nanoparticles with an Organic Molecular Cage. *J Am Chem Soc* 2014; **136**: 1782–5.

18. Mondal B and Mukherjee PS. Cage Encapsulated Gold Nanoparticles as Heterogeneous Photocatalyst for Facile and Selective Reduction of Nitroarenes to Azo Compounds. *J Am Chem Soc* 2018; **140**: 12592–601.

19. Sabater S, Mata JA and Peris E. Catalyst Enhancement and Recyclability by Immobilization of Metal Complexes onto Graphene Surface by Noncovalent Interactions. *ACS Catal* 2014; **4**: 2038−47.

20. Zhao M, Wu Y and Cao JP. Carbon-Based Material-Supported Palladium Nanocatalysts in Coupling Reactions: Discussion on their Stability and Heterogeneity. *Appl Organometal Chem* 2020; **34**: e5539.
